# Author Correction: Engineering the stambomycin modular polyketide synthase yields 37-membered mini-stambomycins

**DOI:** 10.1038/s41467-022-33524-1

**Published:** 2022-10-06

**Authors:** Li Su, Laurence Hôtel, Cédric Paris, Clara Chepkirui, Alexander O. Brachmann, Jörn Piel, Christophe Jacob, Bertrand Aigle, Kira J. Weissman

**Affiliations:** 1 Université de Lorraine, CNRS, IMoPA, F-54000 Nancy, France; 2 Université de Lorraine, INRAE, DynAMic, F-54000 Nancy, France; 3 Université de Lorraine, LIBio, F-54000 Nancy, France; 4 Institute of Microbiology, Eidgenössische Technische Hochschule (ETH) Zurich, 8093 Zurich, Switzerland; 5 Present Address: Max-Planck-Institute for Terrestrial Microbiology, Department of Natural Products in Organismic Interactions, 35043 Marburg, Germany

**Keywords:** Multienzyme complexes, Synthetic biology, Metabolic engineering, Applied microbiology

**Correction to**: *Nature Communications* 10.1038/s41467-022-27955-z, published online 26 January 2022

In the original version of this Article, the compounds **12**, **13**, **14**, and **16** in Figure 5 were incorrectly numbered as compounds **10**, **11**, **12**, and **14**, respectively. These have been corrected in both the PDF and HTML versions of the Article.

