# Supplementary material for: Engineering the stambomycin modular polyketide synthase yields 37-membered mini-stambomycins
Source: Nat Commun. 2022 Jan 26;13:515. doi: 10.1038/s41467-022-27955-z (PMC8792006; doi:10.1038/s41467-022-27955-z)
Supplement: Supplementary file 1 — Supplementary Information [file 41467_2022_27955_MOESM1_ESM.pdf]

Engineering the stambomycin modular polyketide synthase yields 37-membered mini-stambomycins

Su *et al.*

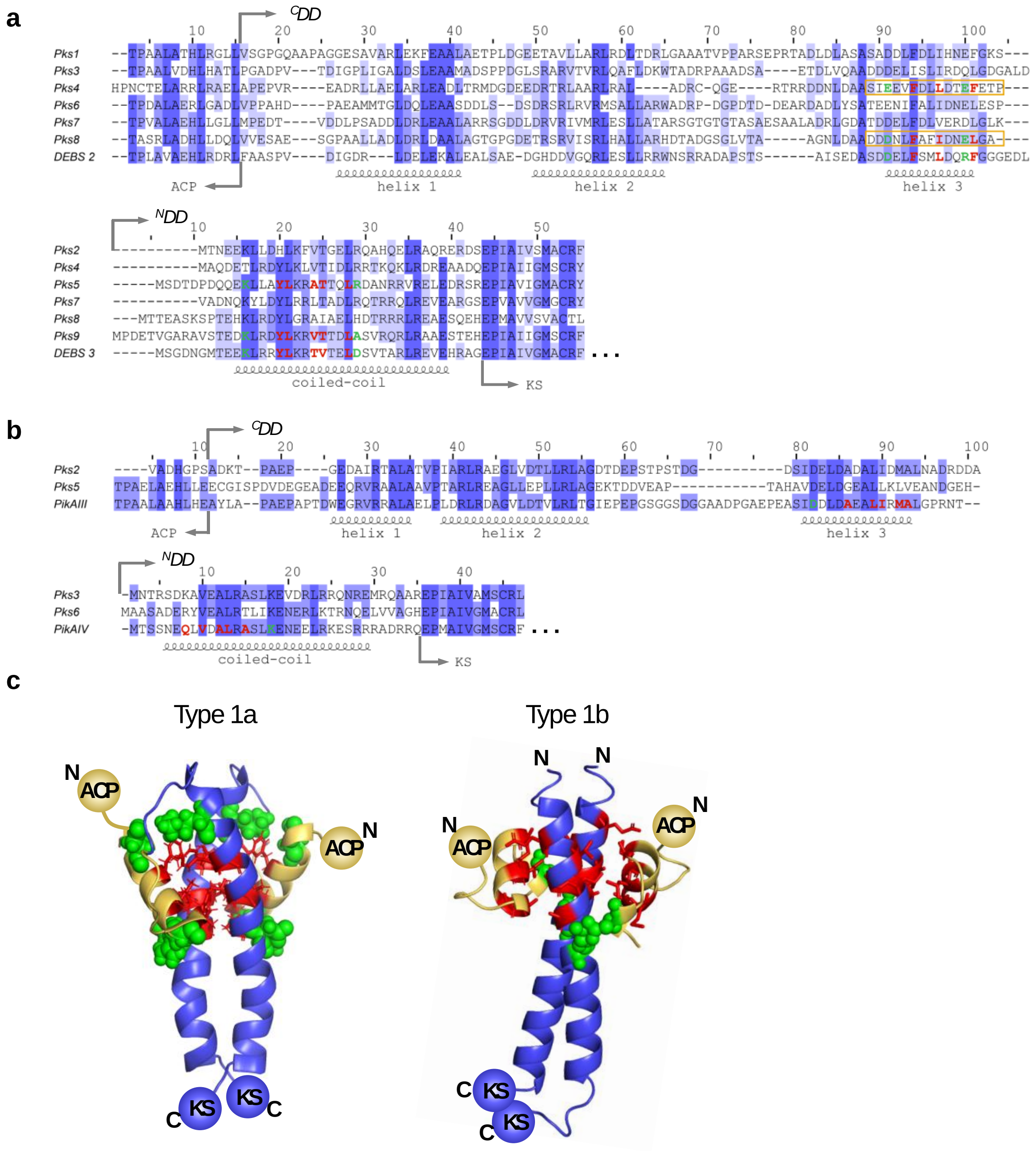

Supplementary Fig. 1. Analysis of putative DDs in the stambomycin PKS. a,b Multiple sequence alignment of stambomycin PKS docking domains. Shown are the regions of α-helical secondary structure as determined by NMR for representative type 1a (DEBS 2–3[1]; PDB: 1PZQ [http://doi.org/10.2210/pdb1PZQ/pdb], 1PZR [http://doi.org/10.2210/pdb1PZR/pdb]) (**a**) and type 1b (PikAIII–IV[2]; PDB: 3F5H [http://doi.org/10.2210/pdb3F5H/pdb]) docking domain complexes (**b**). Conserved residues among the docking domains are highlighted in shades of blue based on the percentage identities. Red and green residues represent, respectively, the amino acids involved in the principal hydrophobic and electrostatic interactions at the docking domain interfaces. The key positions on helix α3 of $^{C}DD_4$ (E, F, L, E, F) were mutated to match those of $^{C}DD_8$ (D, F, I, E, L). The residues shown within the yellow rectangle correspond to the helix swap region. **c** Structures of the type 1a (PDB: 1PZR[1]) and type 1b (PDB: 3F5H[2]) docking complexes showing the key residue positions. In each case, the ^{C}DD is represented in gold and the ^{N}DD in blue, hydrophobic residues as red sticks, and amino acids involved in electrostatic interactions as green spheres. Abbreviations: DEBS, 6-deoxyerythronolide B synthase; Pik, pikromycin synthase; ACP, acyl carrier protein; KS, ketosynthase.

a

KS	Sequence alignment with the putative specificity motifs highlighted
DEBS KS3	-------TATGVFLG**VAKFGY**GEDTA--AAEDVEGYS**V**TGVAPAVASGRISYTM-36aa-VGGAAVMATPG**V**FVDFSRQRALAADGRSKAFGA-------
KS3	-------SRVGVFVG**TNGQDY**AGLFPA-TGSGLEGHV**A**TGSAASVLSGRISYTY-36aa-AGGVTVMAAPT**A**FLEFAHQRGLAADGRCKAFSA-------
**KS4(Pks1/2)**	-------ARVGVFAA**TNGQDY**ATALAADPEAA-DGYL**A**TGAVASVLSGRISYVF-36aa-AGGVTVMATPA**A**FVEFSRQRGLAADGRCKAFGD-------
KS10	-------RSVGMFVG**TNGQDY**PVVLAGSADEGLDAHA**A**TGNAAAVLSGRVSYAF-36aa-AGGVSVMSTEA**A**FTEFARQGGLADDGRCKAFSA-------
**KS15(Pks5/6)**	-------SPVGVYVG**AGTSGY**GIGVPV-AEEA-AGYA**L**TGTATSVLCGRVAYSF-36aa-AGGVTVMATPA**A**FVEFSRQRGLARDGRCKAFAA-------
KS23	-------SDTGVFAG**TNGQDY**LALLLGSAAAT-EGHL**G**TGNSASVLSGRISYTF-36aa-AGGVTAMPLPG**T**FIEFSTQGALSSDGRCKAFSA-------
**KS6(Pks2/3)**	-------SDTGVFVG**ASPSGY**DTVG--RLPESTVGYQ**L**TGSAGSVLSGRLSYVL-36aa-VGGVAVITTPA**A**FTEFGKQGGMASDGRCKSFSA-------
KS18	-------TPTGVFIG**ASPSGY**GVGV--AVPGS-EGHS**L**TGMSGSVLSGRVAYVF-36aa-VGGVAVMTSPG**V**FSEFARQDGLASDGRCRAFSA-------
KS24	-------TRTGVFVG**ASAGGY**AMAG-VLPEGS-ESHA**M**TGTSNSVLSGRVSYVF-36aa-AGGVTVMHSPV**V**FAEFGRQGGLASDGRCRSFAA-------
KS7	-------TRTGVLIG**AASSGY**GLGT--DLFTTAEGHV**L**AGGSNSVISGRVAYSF-36aa-AGGVTVMAAPS**I**FAEFNRQGGLAGDGRCKAFAG-------
KS9	-------RPVGVFAG**ASSSGY**GGAG--DDLEGAGGYL**L**AGTANSVISGRVAYTF-36aa-AGGVTVMVSPA**A**FAEFDRQDGLASDGRCKSFAG-------
**KS11(Pks3/4)**	-------TRTGVFAG**GNDQGY**LRLLANEP--GSVGHQ**L**TGGATAVISGRVAYTL-36aa-AGGVTVMATPG**V**FTEFTRQRGLAADGRCKSFSV-------
KS16	-------SDTAVFAG**TNGQDY**PILLAGDPDIS-EGHQ**G**AGNAAAVLSGRIAYTF-36aa-AGGVTVMSTPT**A**FVEFSRQLGLAADGRCKAFAA-------
**KS19(Pks7/8)**	-------SRTGVFIG**TTGQGY**GINMQAGMA-GTEGYQ**L**TGSATAVLSGRIAYTL-36aa-AGGVTVMPHPI**A**FHEFNRQGGLASDGRCKAFSA-------
**KS14(Pks4/5)**	-------GRTGVFAG**VMYHDY**PSVV--DPEAL-DGYL**G**TANAGSVLSGRIAYTF-36aa-VGGVTVMSGPM**M**FAGFGLEDGSAADGRCKAFAA-------
**KS21(Pks8/9)**	-------SRTGVFTG**VMYNEY**ASRIMSVPD-EVAGHL**G**TGNSGSVVSGRVAYTL-36aa-AGGVAIMVTPN**T**FFDFALSGGLASDGRCKAFSA-------
KS2	-------SDTGVFAG**VMYHDY**AHAAEALPE--TEGYR**A**TGGAGSVVSGRIAYTY-36aa-VGGVTVMSAPG**V**FVDFSKQRGLAPDGRCKSFSA-------
KS20	-------SSTGVFAG**LMYHDY**GATVTVLPE-GVEGYL**G**TGTAGSVLAGRVSYTF-36aa-AGGVTVLSTPG**V**FVDFSRQRGLASDGRCRSFSA-------
KS5	-------EPIGVFAG**AASSYY**GLDK--ALPEDVAGYQ**L**TGGATSVLSGRVAYSF-36aa-AGGVTVMAGFG**V**FSEFSRLDGLAGDARCKAFAE-------
KS12	-------SSTGVYVG**TATSGY**GLGRFAVPD-GSRPHV**L**TGTATSVVSGRLAYTF-36aa-AGGATVMAVPG**I**FTDAAQGGALAPGGRCRAFSS-------
**KS17(Pks6/7)**	-------SRTGVFIG**SSSSAY**GTGLRTLPQ-GVEGHL**L**TGSAPSVVSGRVAYAL-36aa-AGGVTVMTSPG**I**FTEFSKQRGLAADGRCKPFSA-------
KS1	-------SRTGVFVG**ATAQDY**GPRMHE-PAESSEGYL**L**TGTTASVASGRIAYAF-36aa-AGGVTLMATPG**V**LVEFARQDGLSPDGRCKAFGA-------
KS8	-------SSTGVFIG**AAHAQY**GYGM--RLPDNALGHL**M**TGTTTSVASGRLAYTF-36aa-AGGVTVLATPG**V**ITEFDRQRGLASDGRCKAFSA-------
KS13	-------SPTGVFVG**TSFVGY**GIGAQQ-PGNEAEGFF**L**AGTGTAAASGRISYTF-36aa-AGGAAVLATPA**S**FTEFSRQRGLAADGRCKPFAA-------
KS22	-------SRTAVYAG**TGGODY**LAVLAGDPVAG-EGYL**V**TGGSPSVLSGRVAYAF-36aa-VGGVNVISLPS**V**FAEFSKQRGQAADGRCKAFSA-------

Incoming substrate

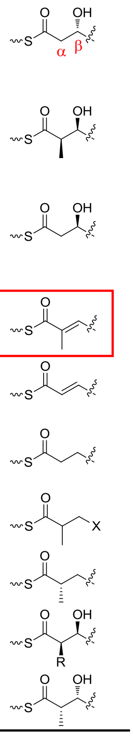

b

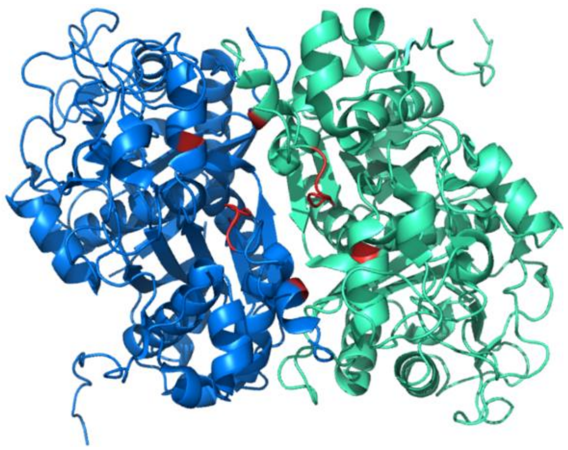

Supplementary Fig. 2. Analysis of the incoming substrates and the putative specificity motifs of the stambomycin PKS KS domains. **a** Sequence alignment showing the residues in the stambomycin PKS KS domains corresponding to those identified in erythromycin PKS (DEBS) KS_3 as mediating substrate specificity[3]. In this figure, stambomycin KS domains which accept substrates of identical structure at the α- and β-positions have been grouped together. The brackets indicate intersubunit junctions where the corresponding KS domains are present, e.g. for KS_4 (Pks1/2). **b** Locations within the KS domains of the putative substrate specificity motifs (VAKFGY-V-V, shown in red within the dimer structure of DEBS KS_3–AT_3 didomain[3] (PDB: 2QO3, from which the AT domains have been omitted)). Abbreviations: KS, ketosynthase; AT, acyl transferase.

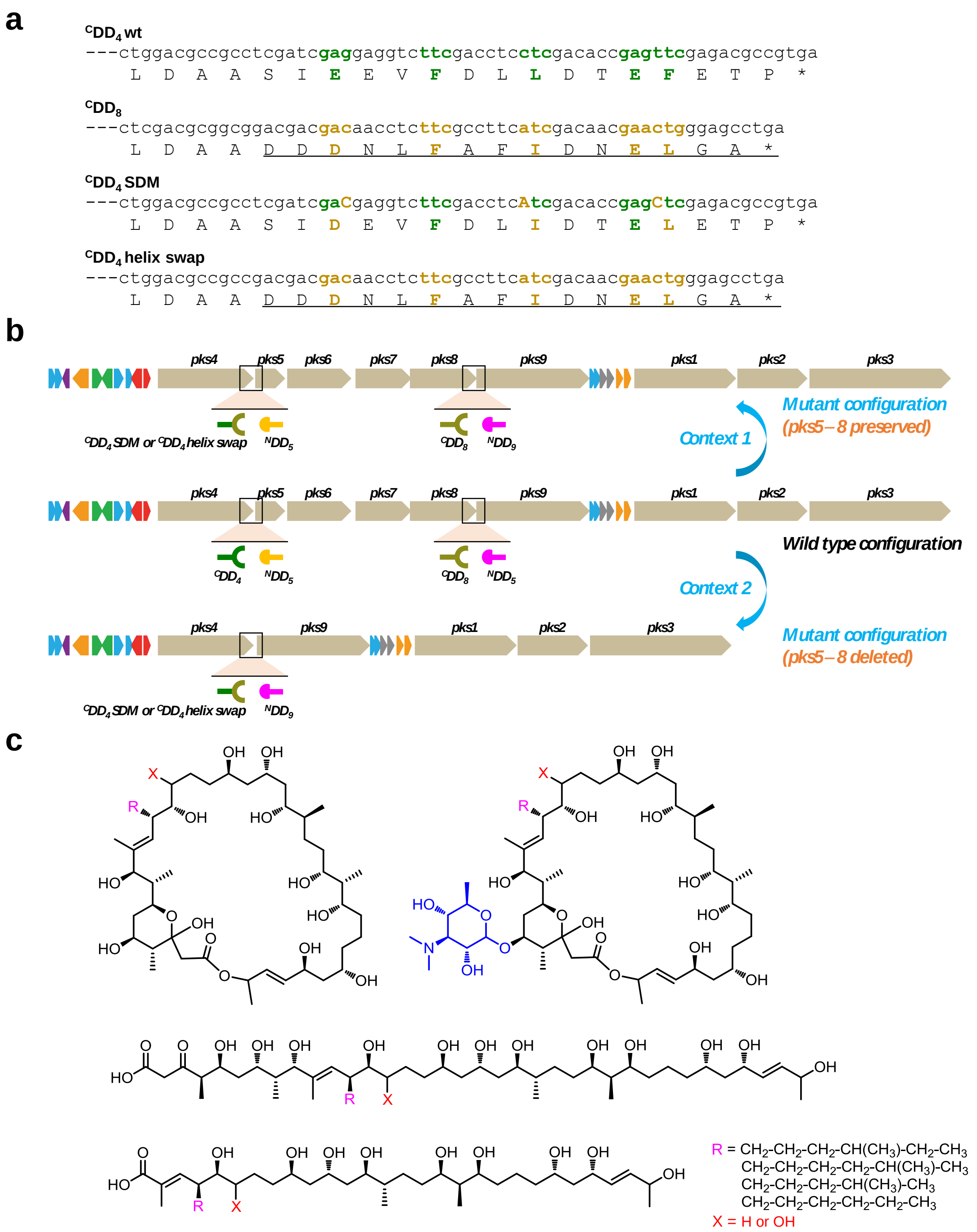

Supplementary Fig. 3. Overview of the interface engineering strategy and the predicted metabolites. a DD modification strategy. Potentially critical residues mediating interaction specificity are colored in dark green in $^{C}DD_4$ wt and orange in $^{C}DD_8$, respectively. As indicated, $^{C}DD_4$ SDM was generated by site-directed mutagenesis (SDM) within $^{C}DD_4$, and $^{C}DD_4$ helix swap by exchange of the full helix α3. **b** Two genetic contexts for the modifications. In the first, the intervening subunits (Pks5–Pks8) were deleted in order to remove competition from subunit 5 for subunit 4, and subunit 8 for subunit 9, while in the second, the intervening genes were preserved. **c** A selection of metabolites potentially generated by a PKS incorporating a novel interface between Pks4 and Pks9. Position X corresponds to the site of potential hydroxylation by cytochrome P450 SamR0478. All indicated stereochemistries are based on prediction only. Abbreviation: ^{C}DD, C-terminal docking domain.

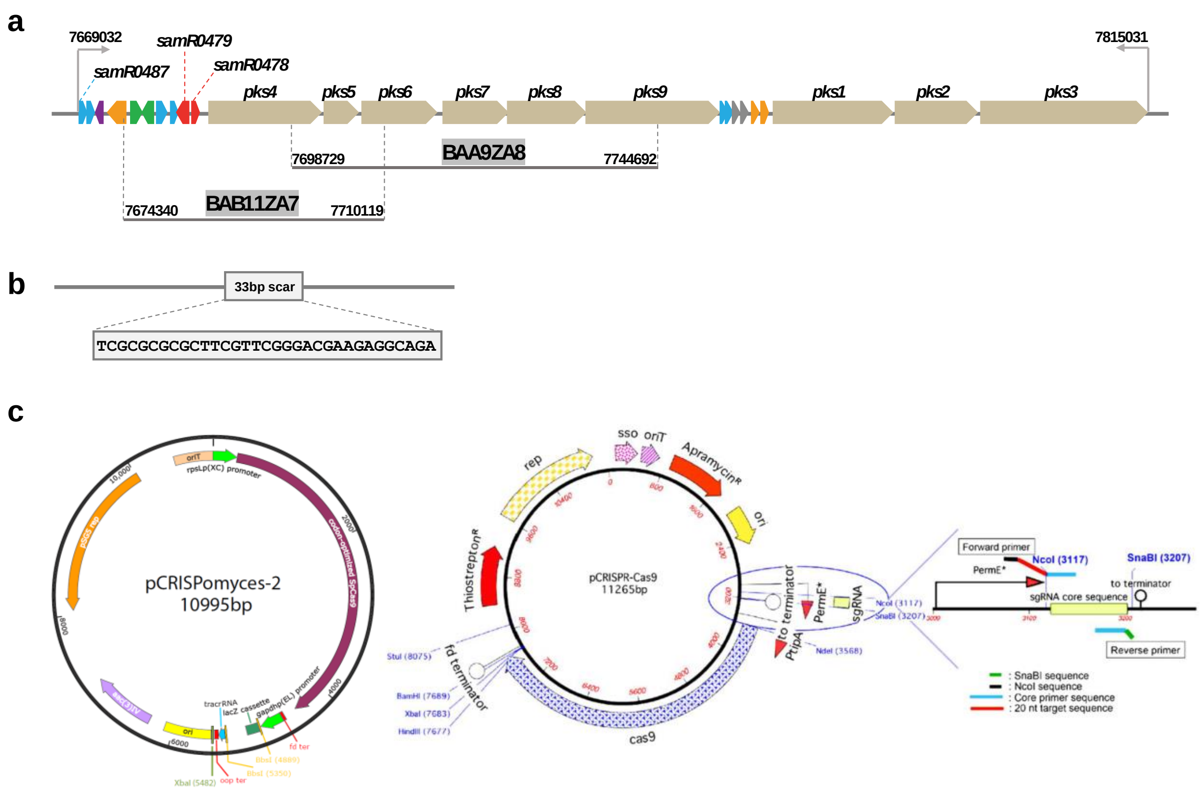

Supplementary Fig. 4. Illustration of key elements used to generate docking domain engineering constructs K7N1–6 and CPN1–5.
a Scheme showing the organization of the cosmids used in this study. **b** For PCR targeting, plasmids pIJ776[4] (http://streptomyces.org.uk/redirect/cassettes/index.html) and pSPM88T[5] were used as templates for amplifying the kanamycin resistance gene and the cassette of "*aatL+aac3(IV)+oriT+aatR*", respectively. pOSK1111[5] was used for cassette excision of "*aatL+aac3(IV)+oriT+aatR*". The final scar sequence left following PCR targeting is shown. **c** Delivery plasmids pCRISPomyces-2[6] (https://www.addgene.org/61737/) and pCRISPR-Cas9[7] (reprinted with permission from Tong, Y., Charusanti, P., Zhang, L., Weber, T. & Lee, S. Y. CRISPR-Cas9 based engineering of actinomycetal genomes. *ACS Synth. Biol.* **4**, 1020–1029 (2015). Copyright 2015, American Chemical Society) were employed to conduct CRISPR-Cas9-mediated genetic manipulation.

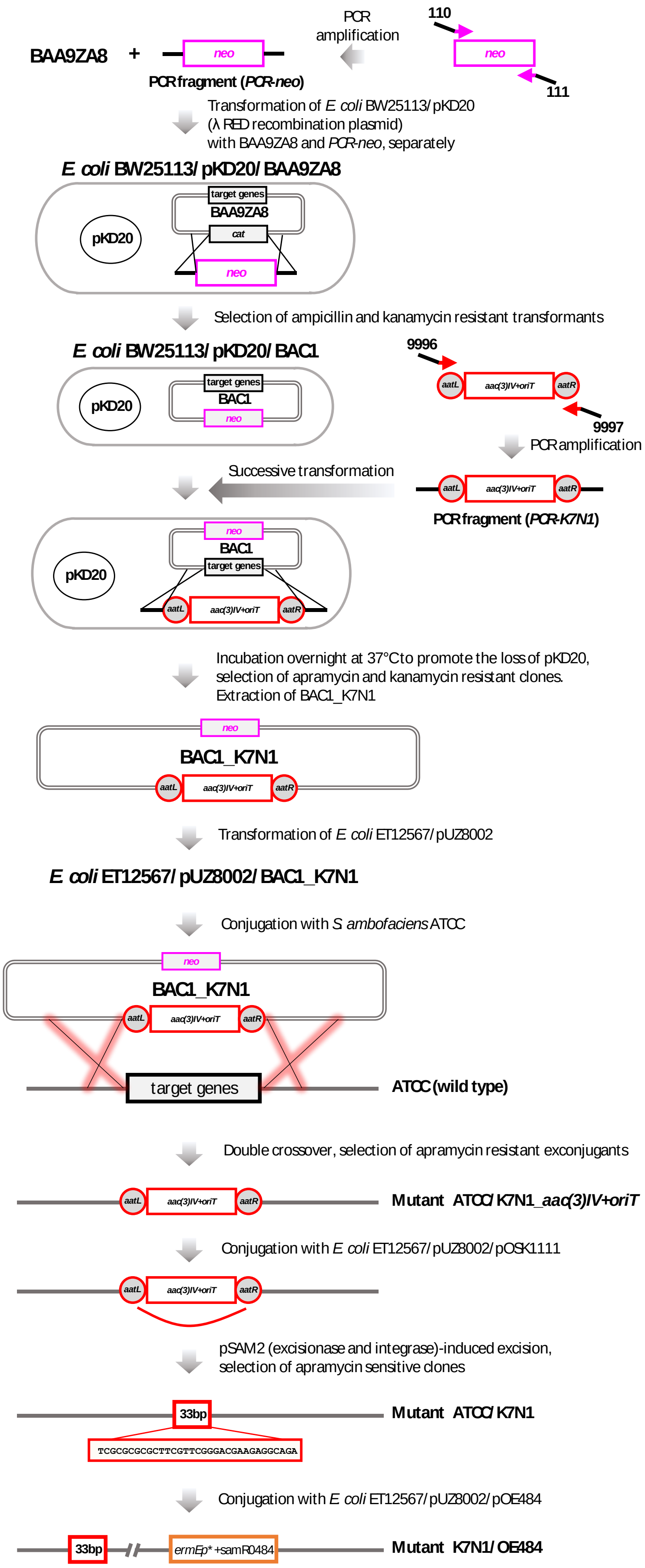
BAA9ZA8
+
neo
PCR fragment (PCR-neo)
PCR amplification
110
neo
111
Transformation of E. coli BW25113/pKD20
(λ RED recombination plasmid)
with BAA9ZA8 and PCR-neo, separately
E. coli BW25113/pKD20/BAA9ZA8
pKD20
target genes
BAA9ZA8
cat
neo
Selection of ampicillin and kanamycin resistant transformants
E. coli BW25113/pKD20/BAC1
pKD20
target genes
BAC1
neo
9996
aatL
aac(3)IV+oriT
aatR
9997
PCR amplification
Successive transformation
aatL
aac(3)IV+oriT
aatR
PCR fragment (PCR-K7N1)
pKD20
neo
BAC1
target genes
aatL
aac(3)IV+oriT
aatR
Incubation overnight at 37°C to promote the loss of pKD20,
selection of apramycin and kanamycin resistant clones.
Extraction of BAC1_K7N1
neo
BAC1_K7N1
aatL
aac(3)IV+oriT
aatR
Transformation of E. coli ET12567/pUZ8002
E. coli ET12567/pUZ8002/BAC1_K7N1
Conjugation with S. ambofaciens ATCC
neo
BAC1_K7N1
aatL
aac(3)IV+oriT
aatR
target genes
ATCC (wild type)
Double crossover, selection of apramycin resistant exconjugants
aatL
aac(3)IV+oriT
aatR
Mutant ATCC/K7N1_aac(3)IV+oriT
Conjugation with E. coli ET12567/pUZ8002/pOSK1111
aatL
aac(3)IV+oriT
aatR
pSAM2 (excisionase and integrase)-induced excision,
selection of apramycin sensitive clones
33bp
Mutant ATCC/K7N1
TCGCGCGCGCTTCGTTCGGGACGAAGAGGCAGA
Conjugation with E. coli ET12567/pUZ8002/pOE484
33bp
ermEp* +samR0484
Mutant K7N1/OE484

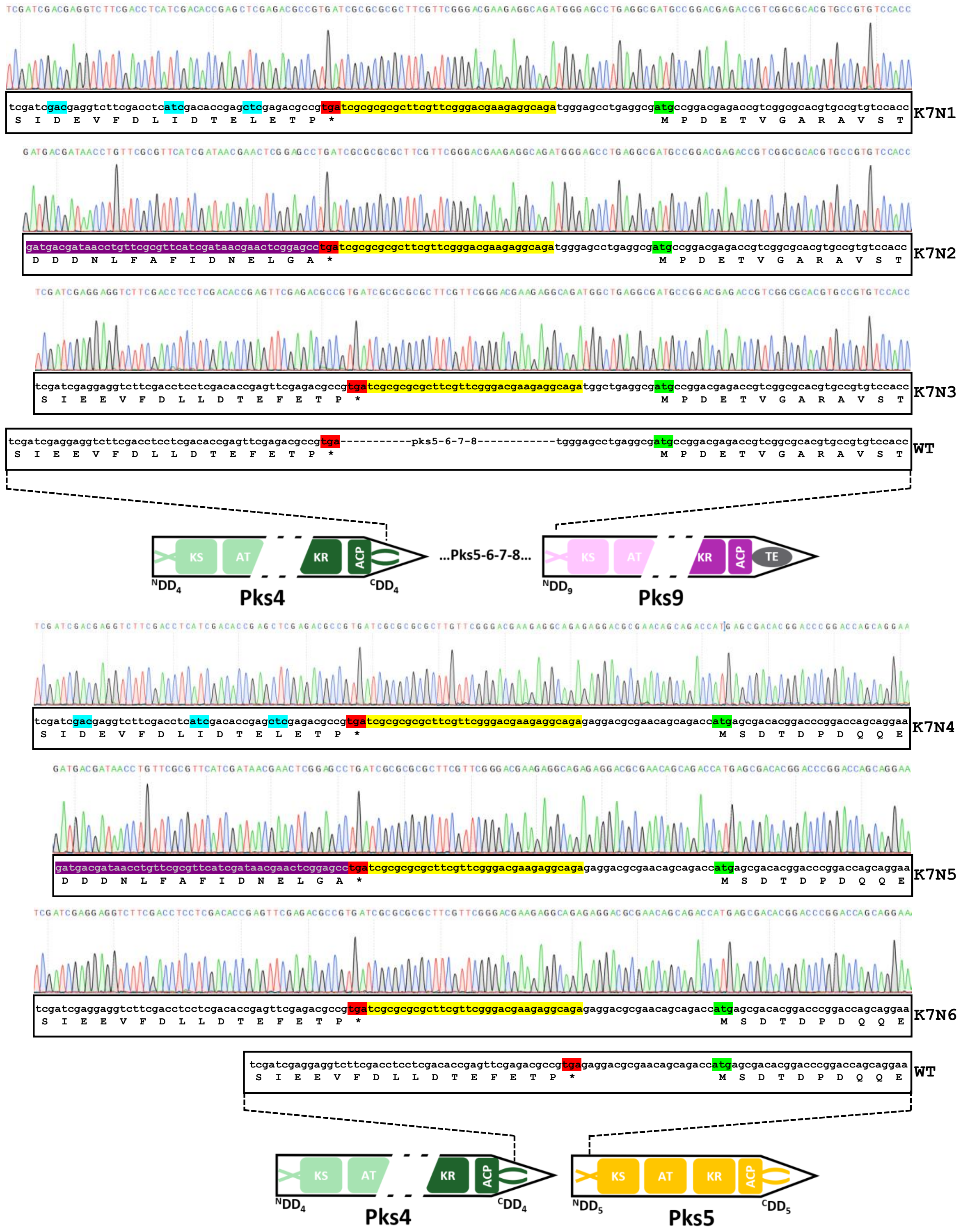

Supplementary Fig. 5. Schematic representation of construction of K7N1–N6 (represented by K7N1) by PCR-targeting, as well as verification of all constructs by sequencing. The sequences colored in turquoise and purple correspond to the SDM (site-directed mutagenesis) and helix-swapped regions of the Pks4 C-terminal docking domain. The locations of the start (green highlighted atg) and stop (red highlighted tga) codons are also indicated. The residual 33 bp scar left following PCR-targeting is highlighted in yellow. Abbreviations: KS, ketosynthase; AT, acyl transferase; ACP, acyl carrier protein; KR, ketoreductase; TE, thioesterase; ^{C}DD, C-terminal docking domain; ^{N}DD, N-terminal docking domain.

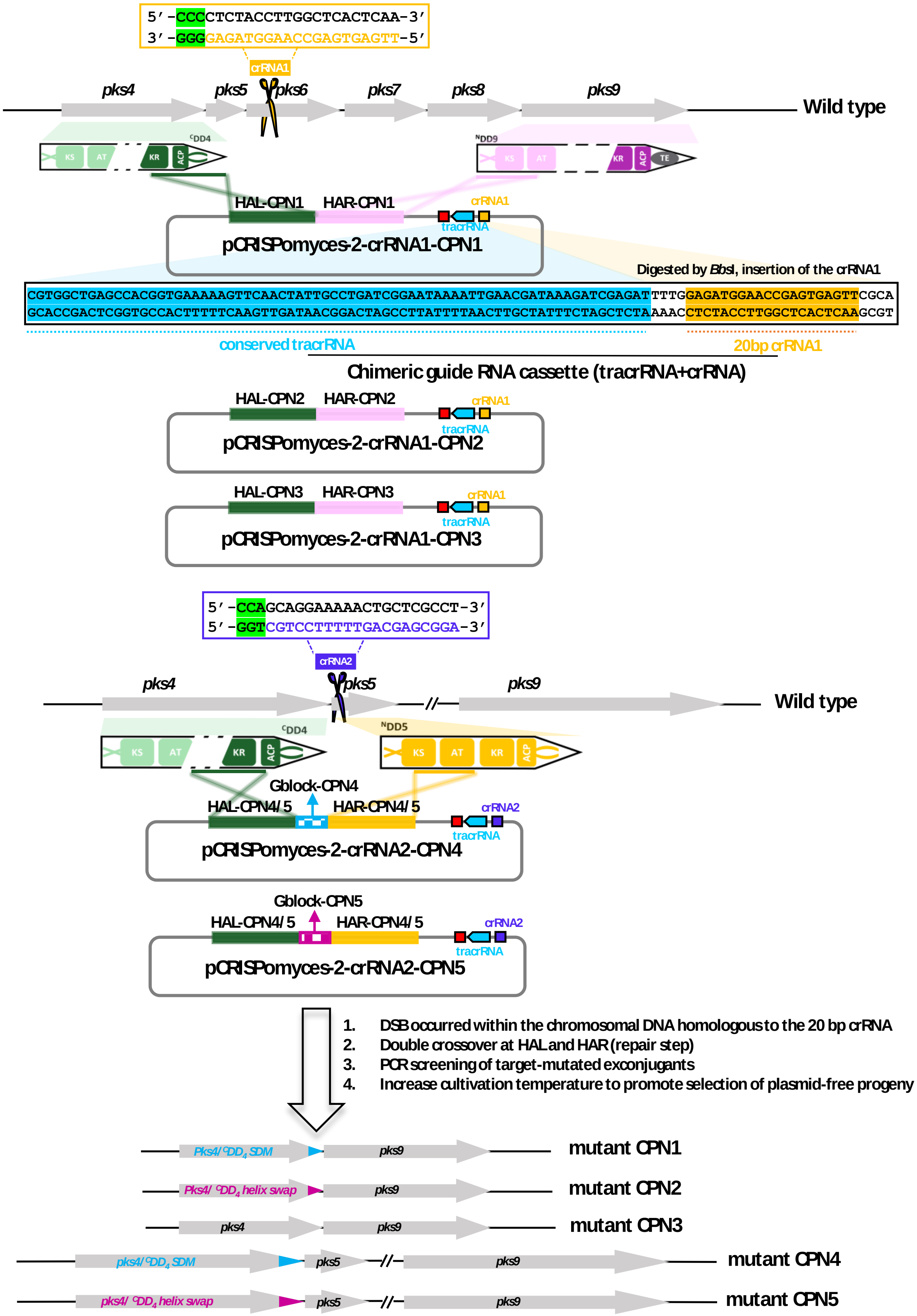

5′-CCCCTCTACCTTGGCTCACTCAA-3′
3′-GGGGAGATGGAACCGAGTGAGTT-5′
crRNA1
pks4
pks5
pks6
pks7
pks8
pks9
Wild type
CDD4
KS
AT
KR
ACP
NDD9
TE
HAL-CPN1
HAR-CPN1
crRNA1
tracrRNA
pCRISPomyces-2-crRNA1-CPN1
Digested by BbsI, insertion of the crRNA1
CGTGGCTGAGCCACGGTGAAAAAGTTCAACTATTGCCTGATCGGAATAAAATTGAACGATAAAGATCGAGATTTTGGAGATGGAACCGAGTGAGTTCGCA
GCACCGACTCGGTGCCACTTTTTCAAGTTGATAACGGACTAGCCTTATTTTAACTTGCTATTTCTAGCTCTAAAACCTCTACCTTGGCTCACTCAAGCGT
conserved tracrRNA
20bp crRNA1
Chimeric guide RNA cassette (tracrRNA+crRNA)
HAL-CPN2
HAR-CPN2
pCRISPomyces-2-crRNA1-CPN2
HAL-CPN3
HAR-CPN3
pCRISPomyces-2-crRNA1-CPN3
5′-CCAGCAGGAAAAACTGCTCGCCT-3′
5′-GGTCGTCCTTTTTGACGAGCGGA-3′
crRNA2
pks4
pks5
pks9
Wild type
CDD4
NDD5
Gblock-CPN4
HAL-CPN4/5
HAR-CPN4/5
crRNA2
tracrRNA
pCRISPomyces-2-crRNA2-CPN4
Gblock-CPN5
HAL-CPN4/5
HAR-CPN4/5
pCRISPomyces-2-crRNA2-CPN5
1. DSB occurred within the chromosomal DNA homologous to the 20 bp crRNA
2. Double crossover at HAL and HAR (repair step)
3. PCR screening of target-mutated exconjugants
4. Increase cultivation temperature to promote selection of plasmid-free progeny
Pks4/CDD4 SDM
pks9
mutant CPN1
Pks4/CDD4 helix swap
pks9
mutant CPN2
pks4
pks9
mutant CPN3
pks4/CDD4 SDM
pks5
pks9
mutant CPN4
pks4/CDD4 helix swap
pks5
pks9
mutant CPN5

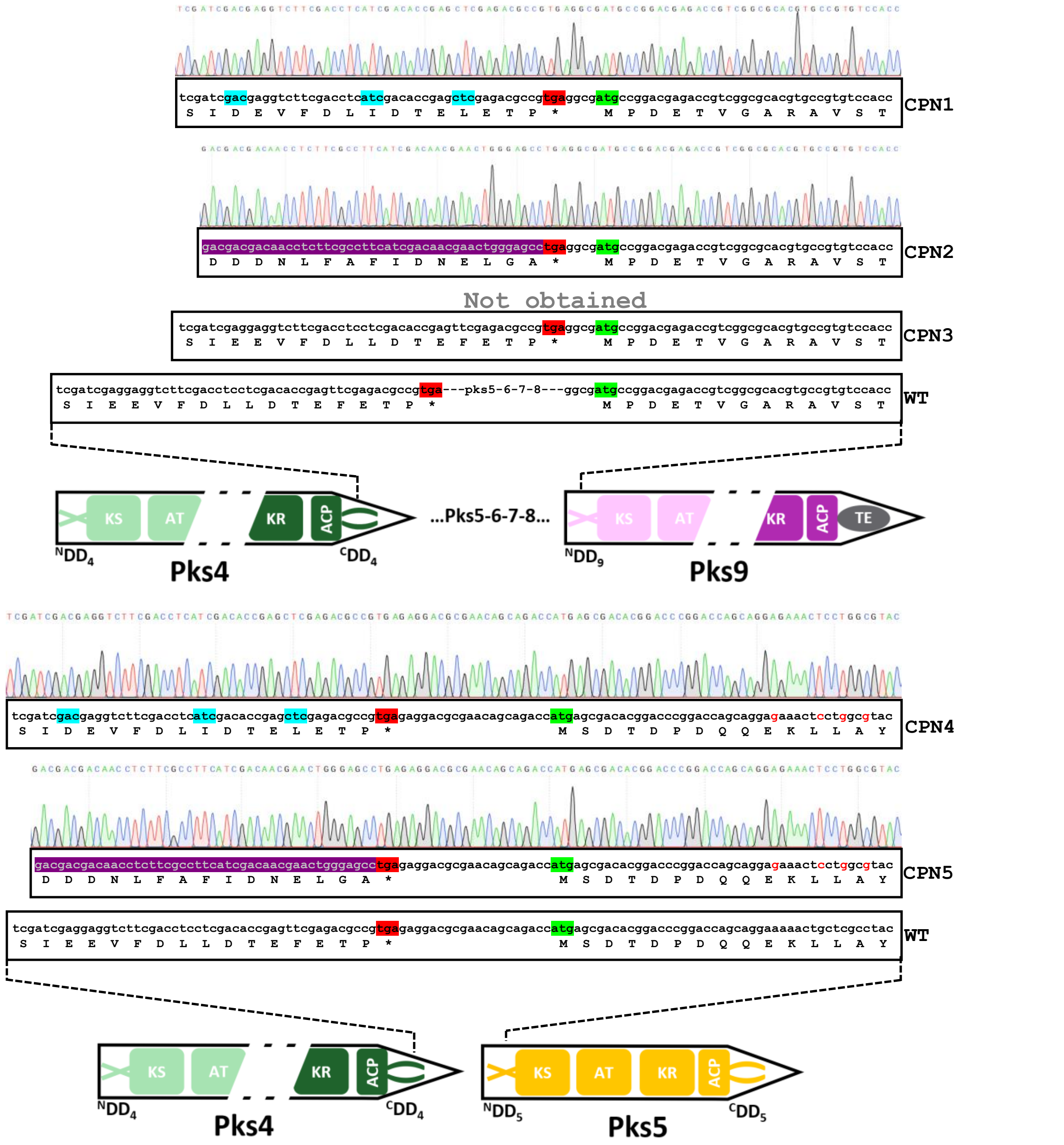

Supplementary Fig. 6. Schematic representation of CRISPR-Cas9-mediated mutant construction of CPN1–CPN5, and verification of all constructs by sequencing. The significance of the turquoise, purple, green and red highlighted sequences is as in panel **d**. The additional regions in red font (in CPN4 and CPN5) indicate the nucleotides mutated within the designed crRNA2 in order to avoid unwanted cleavage by Cas9 within the genome of the newly-obtained mutant. Abbreviations: KS, ketosynthase; AT, acyl transferase; ACP, acyl carrier protein; KR, ketoreductase; TE, thioesterase; ^{C}DD, C-terminal docking domain; ^{N}DD, N-terminal docking domain.

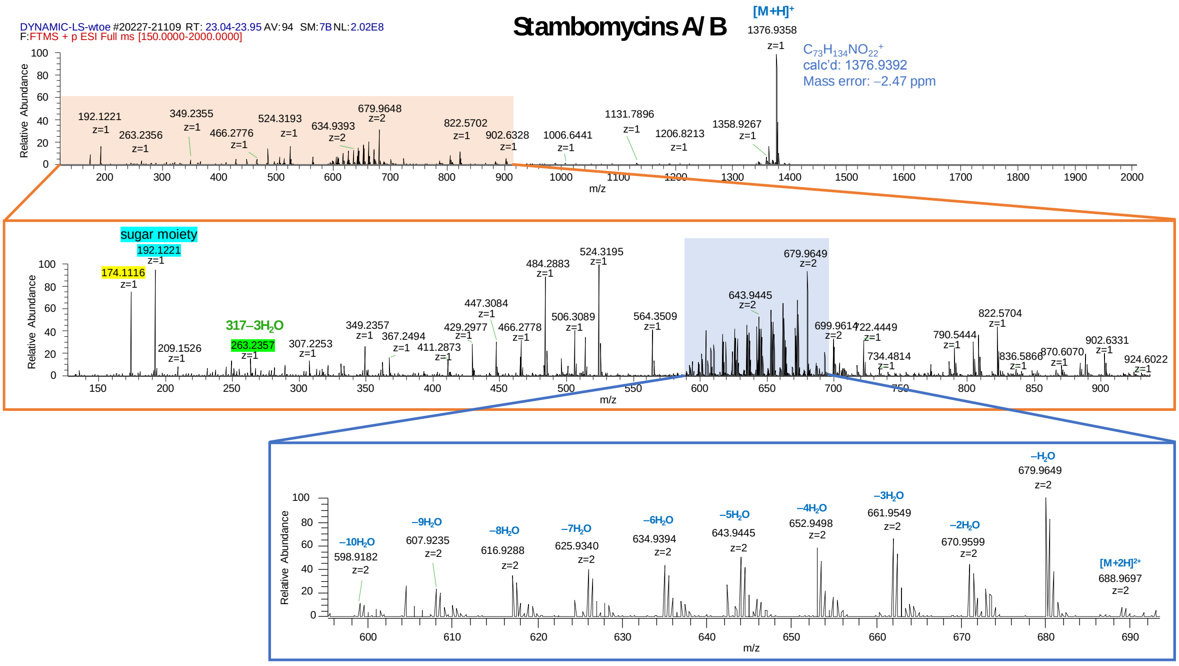
Stambomycins A/ B
DYNAMIC-LS-wtoe #20227-21109 RT: 23.04-23.95 AV: 94 SM: 7B NL: 2.02E8
F: FTMS + p ESI Full ms [150.0000-2000.0000]
[M+H]+
1376.9358
z=1
C73H134NO22+
calc'd: 1376.9392
Mass error: –2.47 ppm
sugar moiety
192.1221
174.1116
317–3H2O
263.2357
679.9648
z=2
Relative Abundance
m/z
–H2O
–2H2O
–3H2O
–4H2O
–5H2O
–6H2O
–7H2O
–8H2O
–9H2O
–10H2O
[M+2H]2+
688.9697
z=2

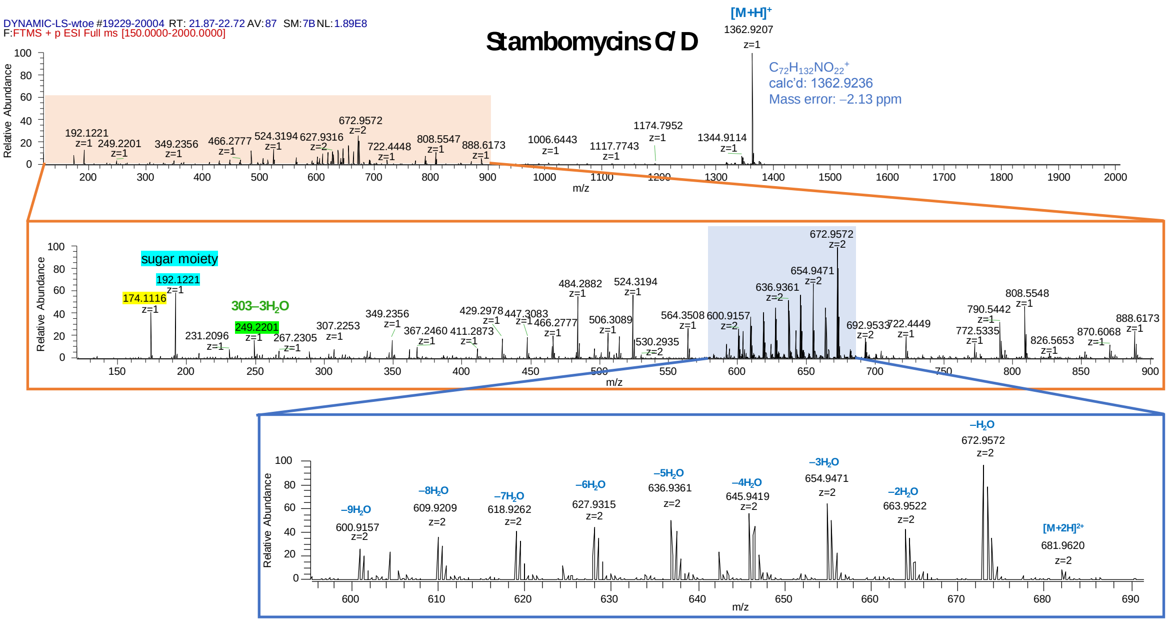
Stambomycins C/ D
DYNAMIC-LS-wtoe #19229-20004 RT: 21.87-22.72 AV: 87 SM: 7B NL: 1.89E8
F: FTMS + p ESI Full ms [150.0000-2000.0000]
[M+H]+
1362.9207
z=1
C72H132NO22+
calc'd: 1362.9236
Mass error: –2.13 ppm
sugar moiety
192.1221
174.1116
303–3H2O
249.2201
672.9572
z=2
Relative Abundance
m/z
–H2O
–2H2O
–3H2O
–4H2O
–5H2O
–6H2O
–7H2O
–8H2O
–9H2O
[M+2H]2+
681.9620
z=2

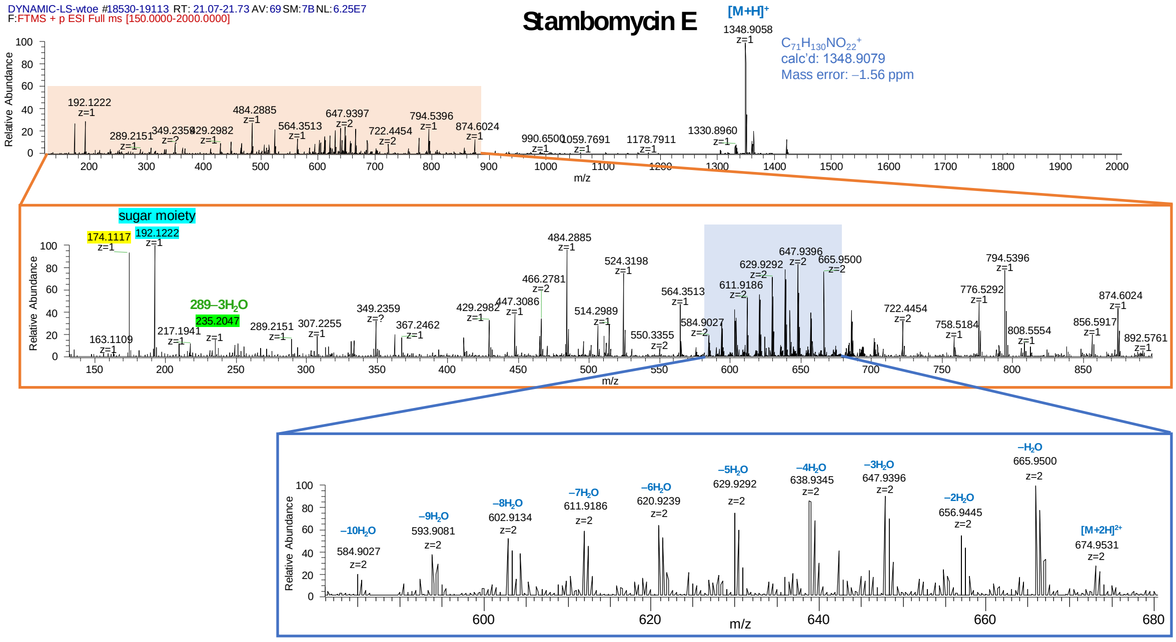

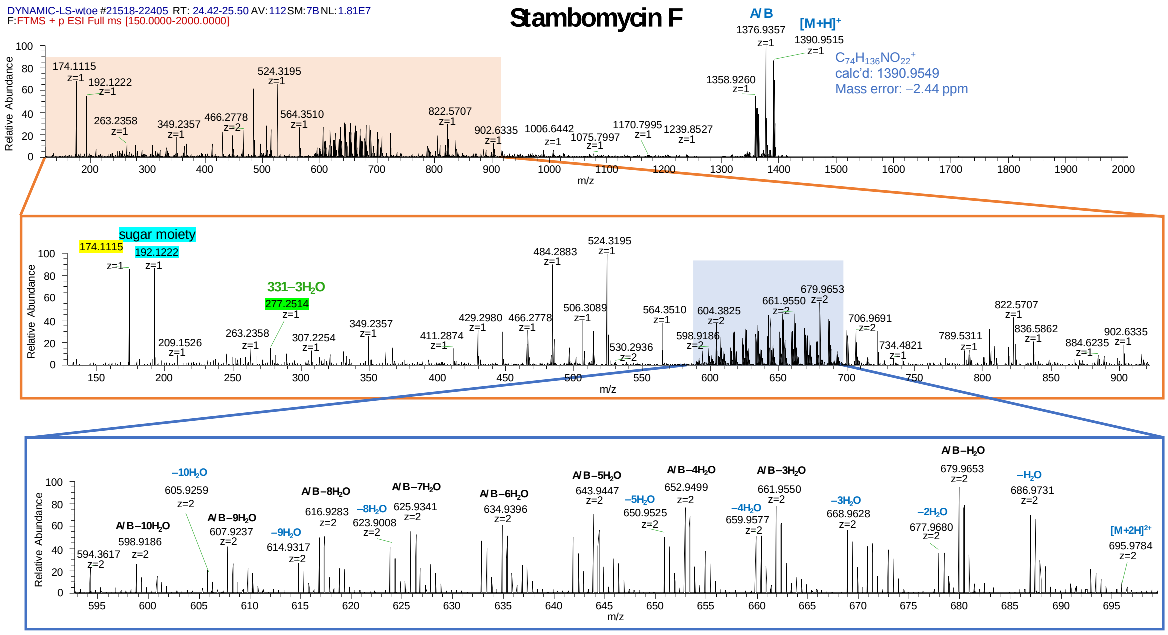

Proposed origin of the peaks indicated in green which report on the nature of the R group:

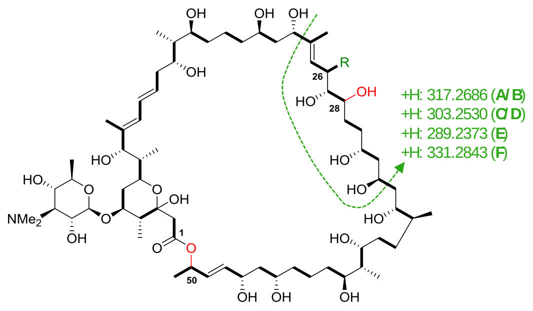

Supplementary Fig. 7. MS analysis of stambomycins 1 (represented by data from analysis of ATCC/OE484).

MS/MS fragments of stambomycins **1A/B**

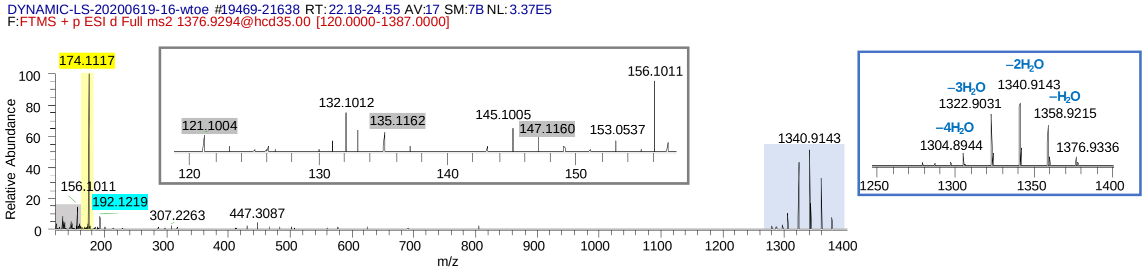

MS/MS fragments of stambomycins **1C/D**

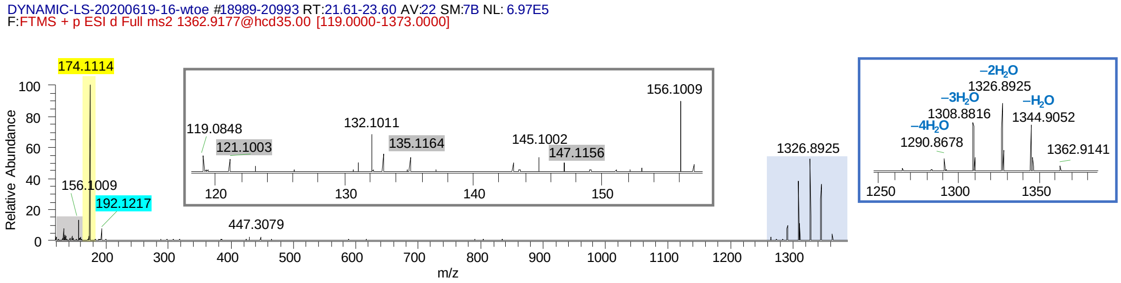

MS/MS fragments of stambomycin **1E**

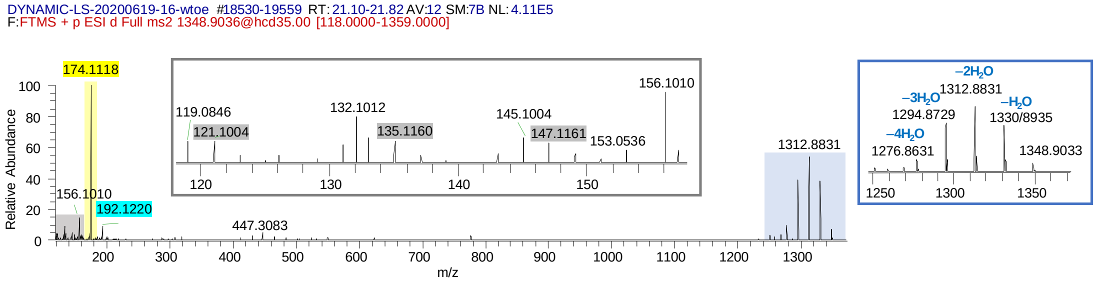

MS/MS fragments of stambomycin **1F**

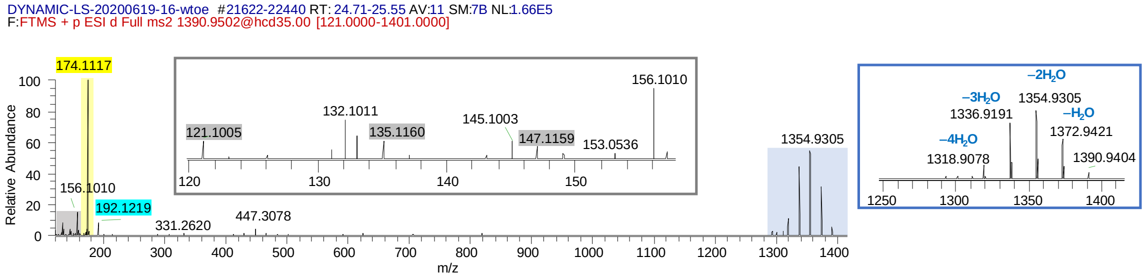

Supplementary Fig. 8. MS/MS analysis of stambomycins 1 (represented by data from analysis of ATCC/OE484). The stambomycins A–F exhibit a subset of the fragments common to **4**–**7** (highlighted in grey, compare to **Supplementary Fig. 15**), and additionally fragments of 192.1 (corresponding to the sugar moiety), and 174.1 (highlighted in yellow), which are not present in the linear, truncated forms **4**–**7** (**Supplementary Fig. 15**).

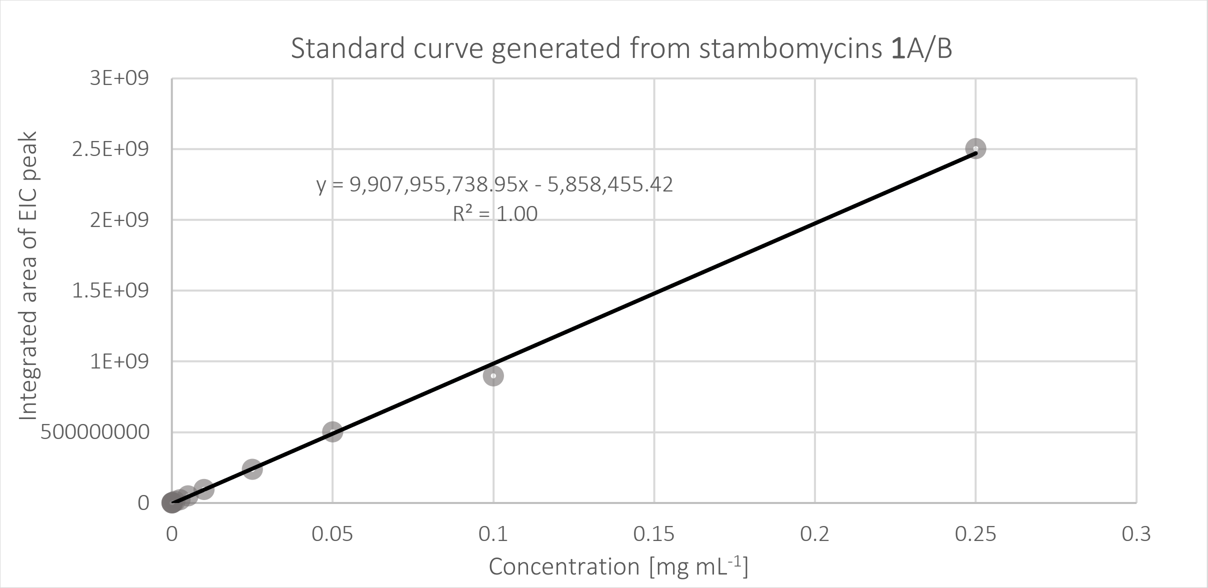

Supplementary Fig. 9. Standard curve for quantification of novel metabolites based on stambomycins 1A/B. Accurate quantification of the full-length stambomycins was achieved by generation of a standard curve based on purified stambomycins **1**A/B[8]. Peak areas corresponding to stambomycins **1**A/B were calculated from the extracted ion chromatograms (using $[M+H]^+$ = 1376.9392 and $[M+2H]^{2+}$ = 688.9736). A dilution series with a concentration range of 0.00001–0.5 mg mL^{-1} was analyzed. The integrated area 3160735573 of the 0.5 mg mL^{-1} dilution sample was out of the linear range, and so was not included in the final analysis. The standards with concentrations of 0.25, 0.1, 0.025, 0.005, and 0.001 mg mL^{-1} were measured twice, and thus the areas shown in the table represent average values. This analysis yielded an essentially linear standard curve (R^2 =1.0) and equation: y = 9907955738.95x – 5858455.42. Source data are provided as a Source Data file.

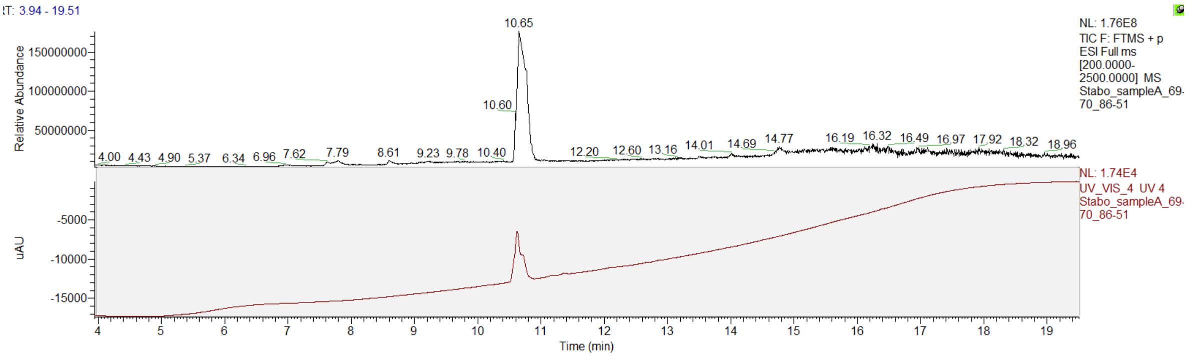

MS spectra of 50-deoxystambomycins **2**A–D

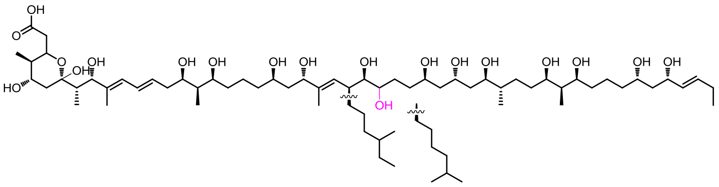

50-deoxystambomycins A/ B

$C_{65}H_{121}O_{19}^{+}$

calc'd: 1205.8497

Mass error: –0.58 ppm

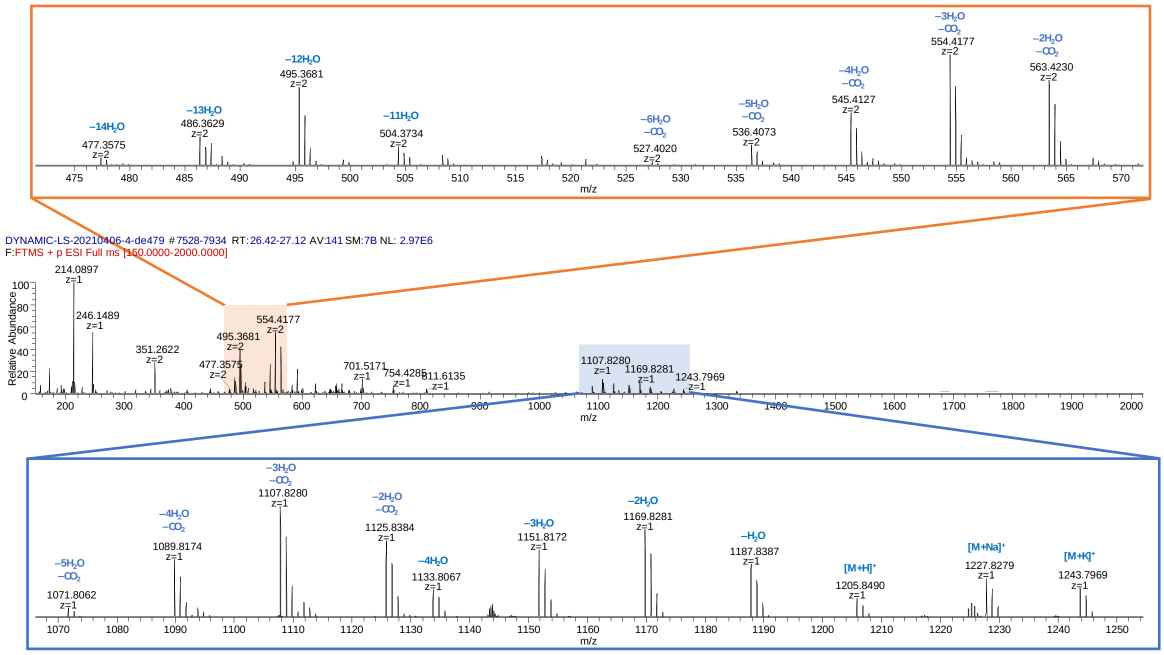

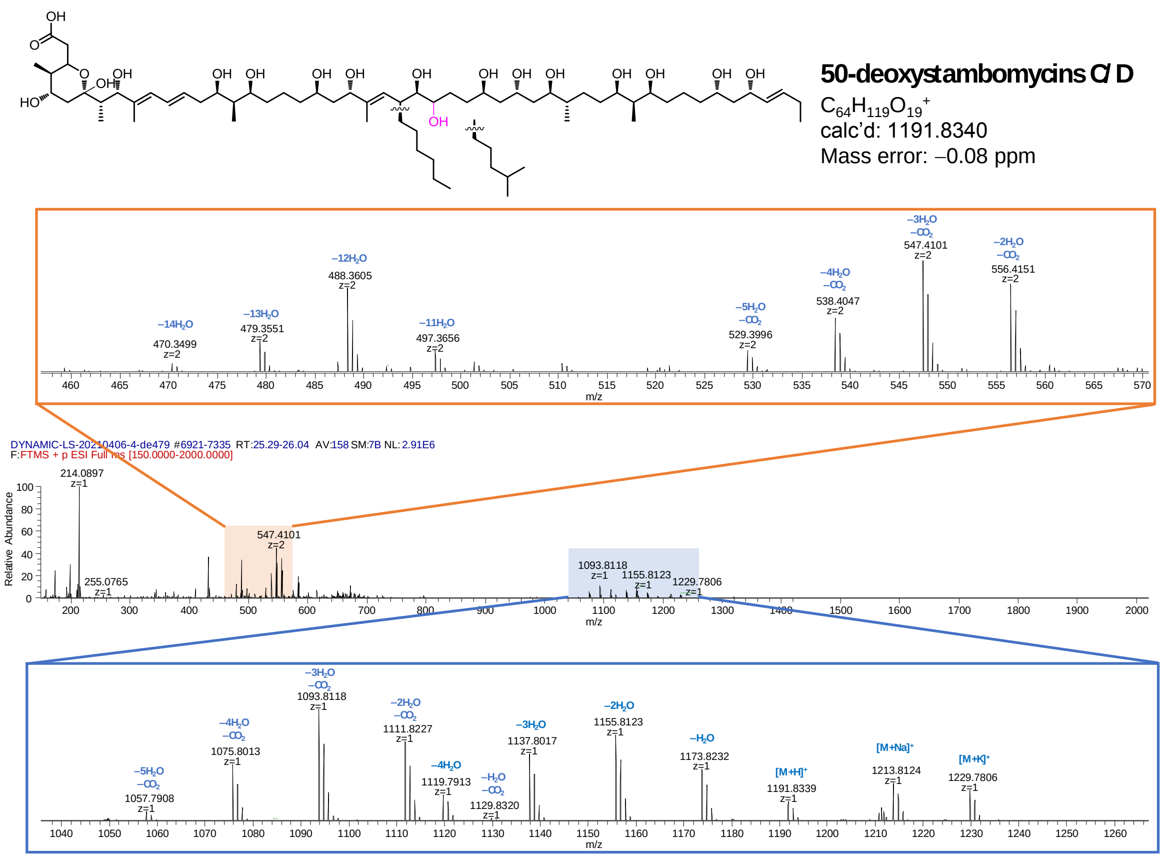

MS² spectra of 50-deoxystambomycins **2**A–D

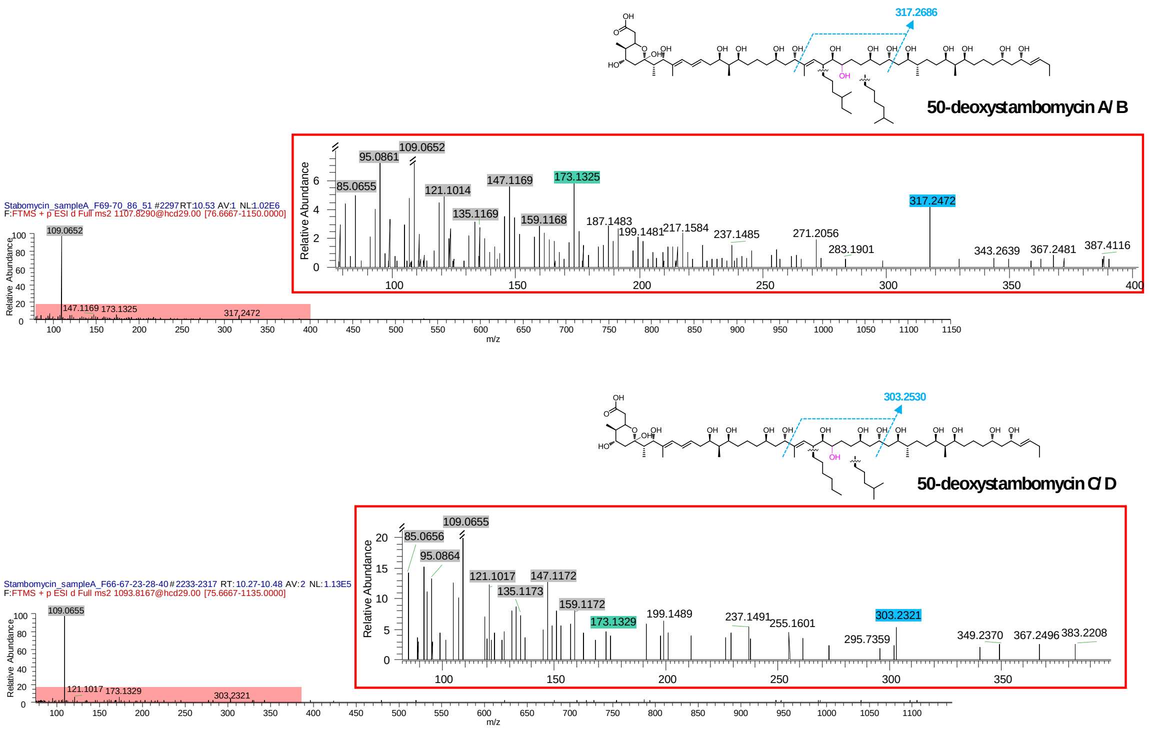

Supplementary Fig. 10. Purification of 50-deoxystambomycins 2, and structure verification based on MS/MS analysis.

a

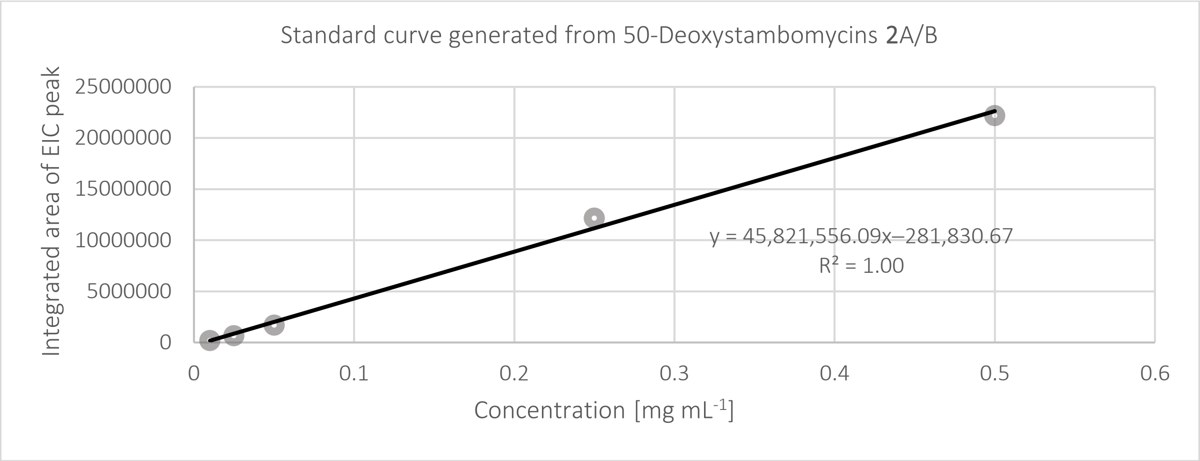

b

Sample injected (0.25 mg mL^{-1})	Average area (MS)	Average area (UV 254 nm)	Average area (UV 238 nm)
Stambomycins **1**A/B	2503398424	3669590	7481724
50-Deoxystambomycins **2**A/B	12169511	91661	226952

Supplementary Fig. 11. Standard curve for quantification of novel metabolites based on 50-deoxystambomycins 2**A/B. a** To aid in quantifying the novel metabolites, we generated a standard curve based on mixtures of 50-deoxystambomycins **2**A/B[9]. For this, the compounds of interest were purified from a previously-described strain in which the C-50 hydroxylase SamR0479 had been inactivated (*S. ambofaciens* ATCC/OE484/Δ479)[9]. Purification from 1 L of medium yielded 0.85 mg of 50-deoxystambomycins A/B, substantially lower than the previous reported yields of 3.2 mg L^{-1} [9]. Shown are the MS/MS data acquired on the compounds which we used to compare with the newly-generated derivatives. Analysis of solutions of purified 50-deoxystambomycins **2**A/B at a range of concentrations (0.00001–0.5 mg mL^{-1}) revealed that the UV and MS detection limits of these metabolites are substantially lower than those of the wild type stambomycins **1**A–D (compare to **Supplementary Fig. 9**) Source data are provided as a Source Data file. **b** To confirm the result in **a**, we directly compared the detection sensitivity of the same concentrations of stambomycins A/B **1** and their 50-deoxy counterparts **2**A/B (0.25 mg mL^{-1}) by MS (based on peak areas corresponding to the predominant ionic forms, i.e. $[M+H]^+$ and $[M+2H]^{2+}$ for the stambomycins **1**A/B, and $[M–3H_2O–CO_2+H]^+$ and $[M–3H_2O–CO_2+2H]^{2+}$ for the 50-deoxystambomycins **2**A/B), and UV (254 and 238 nm), with each sample analyzed successively in triplicate, followed by calculation of average peak areas. This experiment showed that detection of the wild type stambomycins **1** was 206-fold more sensitive by MS and ca. 36-fold more sensitive by UV-Vis than the linear derivatives **2**. Given this result, we opted to use the MS standard curve generated from the wild stambomycins as it was produced using a range of concentrations beyond that accessible with the 50-deoxystambomycins **2**A/B standard, and to introduce a correction factor for the engineered derivatives in order to be able to estimate a range of yields. Nonetheless, calculation of the upper yield limits directly from the standard curve generated with the 50-deoxystambomycins **2**A/B standard gave essentially the same results (**Supplementary Table 3**).

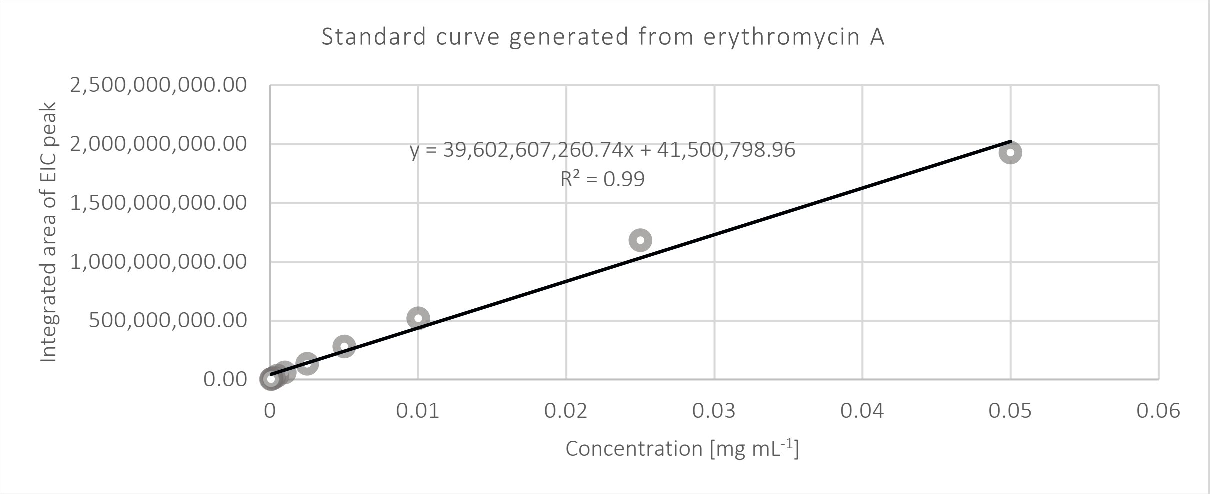

Supplementary Fig. 12. Standard curve for quantification of novel metabolites based on erythromycin A. We also analyzed erythromycin A **3**, which incorporates the amino sugar β-D-desosamine. Peak areas corresponding to erythromycin A were calculated from the extracted ion chromatograms (using $[M+H]^+$ = 734.4683). This analysis yielded an essentially linear standard curve (R^2 =0.99) and equation: y = 39,602,607,260.74x – 41,500,798.96. Detection of erythromycin A **3** occurred with approximately 4-fold greater sensitivity than for stambomycins **1**A/B, confirming that the presence of nitrogen substantially boosts detection sensitivity under our conditions. Source data are provided as a Source Data file.

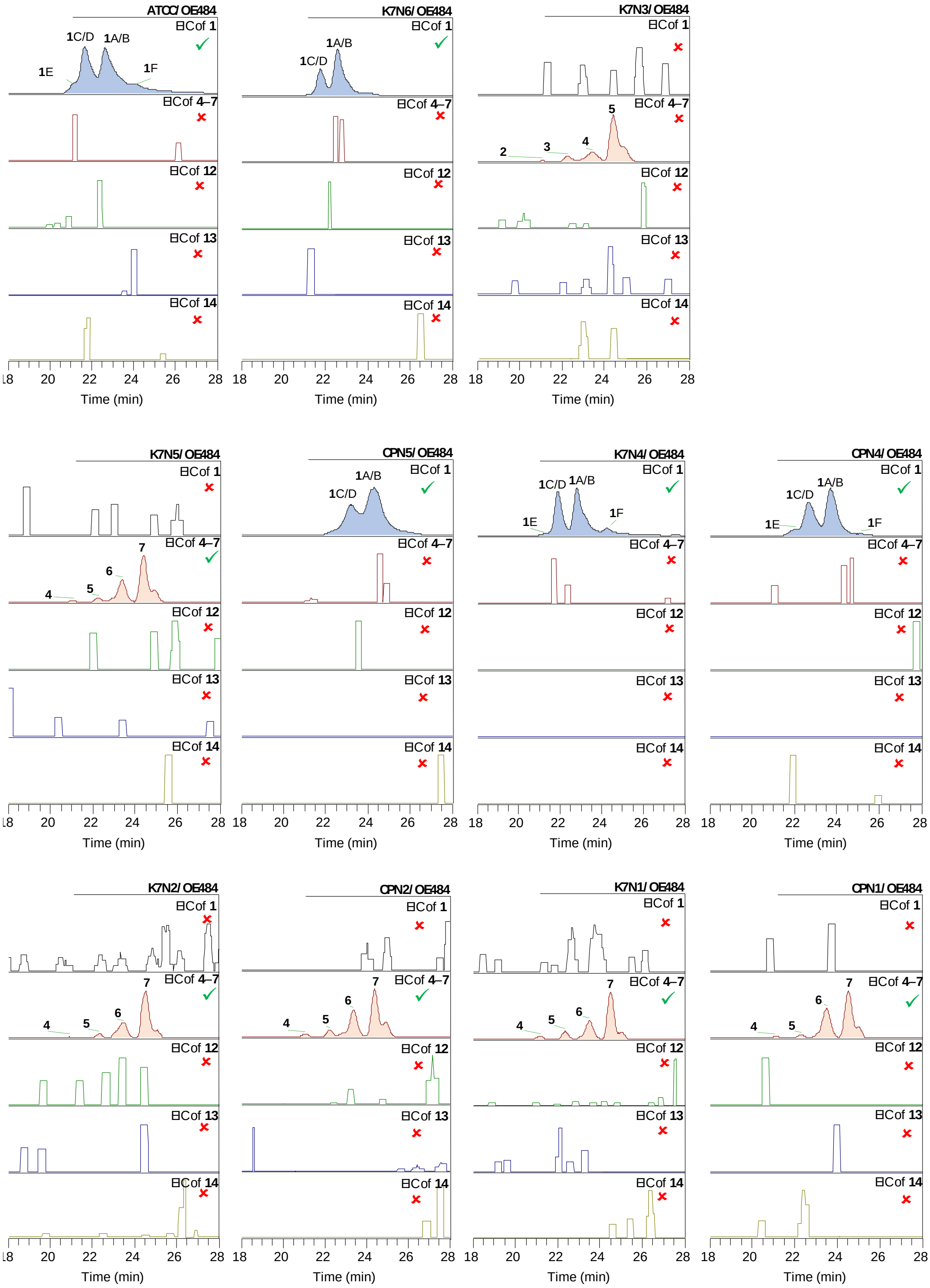

Supplementary Fig. 13. HPLC-MS analysis of all docking domain engineering mutants. EIC (extracted ion chromatogram) analysis for the presence of stambomycins **1**, shunt products **4**–**7** and mini-stambomycins **12**–**14**, in extracts of all constructs, by comparison to wild type. The EICs representing all possible masses for each compound family are listed in Supplementary Table 3.

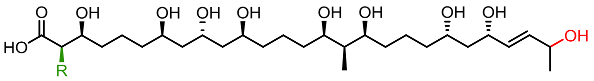

Shunt product (M12) **4**

Chemical Formula: $C_{35}H_{68}O_{11}$ R = CH_2-CH_2-CH_2-$CH(CH_3)$-CH_3
CH_2-CH_2-CH_2-CH_2-CH_2-CH_3

$[M+H]^+$: $C_{35}H_{69}O_{11}^+$, Calc'd: 665.4834, Mass error = –0.45 ppm

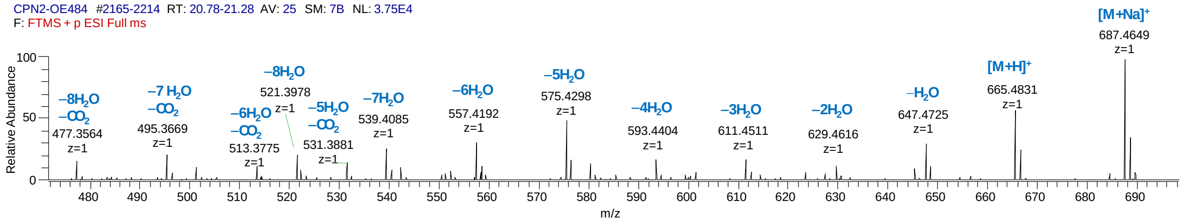

Shunt product (M12) **5**

Chemical Formula: $C_{36}H_{70}O_{11}$ R = CH_2-CH_2-CH_2-$CH(CH_3)$ -CH_2-CH_3
CH_2-CH_2-CH_2-CH_2-CH (CH_3) -CH_3

$[M+H]^+$: $C_{36}H_{71}O_{11}^+$, Calc'd: 679.4991, Mass error = –0.74 ppm

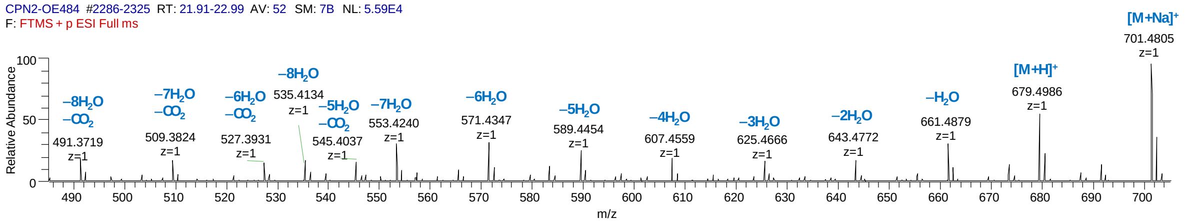

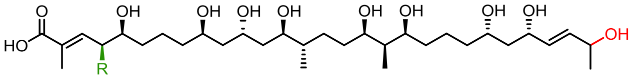

Shunt product (M13) **6**

Chemical Formula: $C_{38}H_{72}O_{11}$ R = CH_2-CH_2-CH_2-$CH(CH_3)$-CH_3
CH_2-CH_2-CH_2-CH_2-CH_2-CH_3

$M+H]^+$: $C_{38}H_{73}O_{11}^+$, Calc'd: 705.5147, Mass error = –0.43 ppm

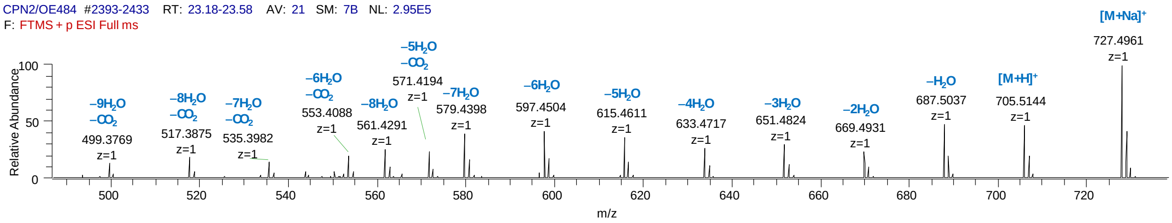

Shunt product (M13) **7**

Chemical Formula: $C_{39}H_{74}O_{11}$ R = CH_2-CH_2-CH_2-$CH(CH_3)$ -CH_2-CH_3
CH_2-CH_2-CH_2-CH_2-CH (CH_3) -CH_3

$[M+H]^+$: $C_{39}H_{75}O_{11}^+$, Calc'd: 719.5304, Mass error = –0.69 ppm

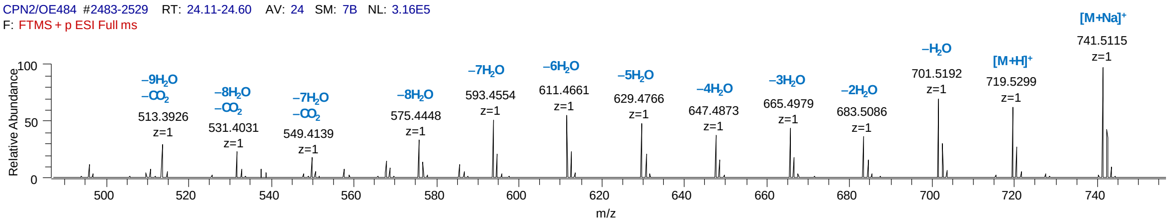

Supplementary Fig. 14. MS analysis of shunt products 4–7 **(represented by data from analysis of CPN2/OE484).** This analysis provides multiple lines of evidence supporting our structural assignments: i) the presence of both C/D (**4**, **6**) and A/B (**5**, **7**) analogues released from modules 12 and 13, respectively; ii) peaks in the MS spectra corresponding to loss of all 9 hydroxyl groups in the molecules as water; and iii) multiple peaks corresponding to loss of the terminal CO_2 group, consistent with linear structures.

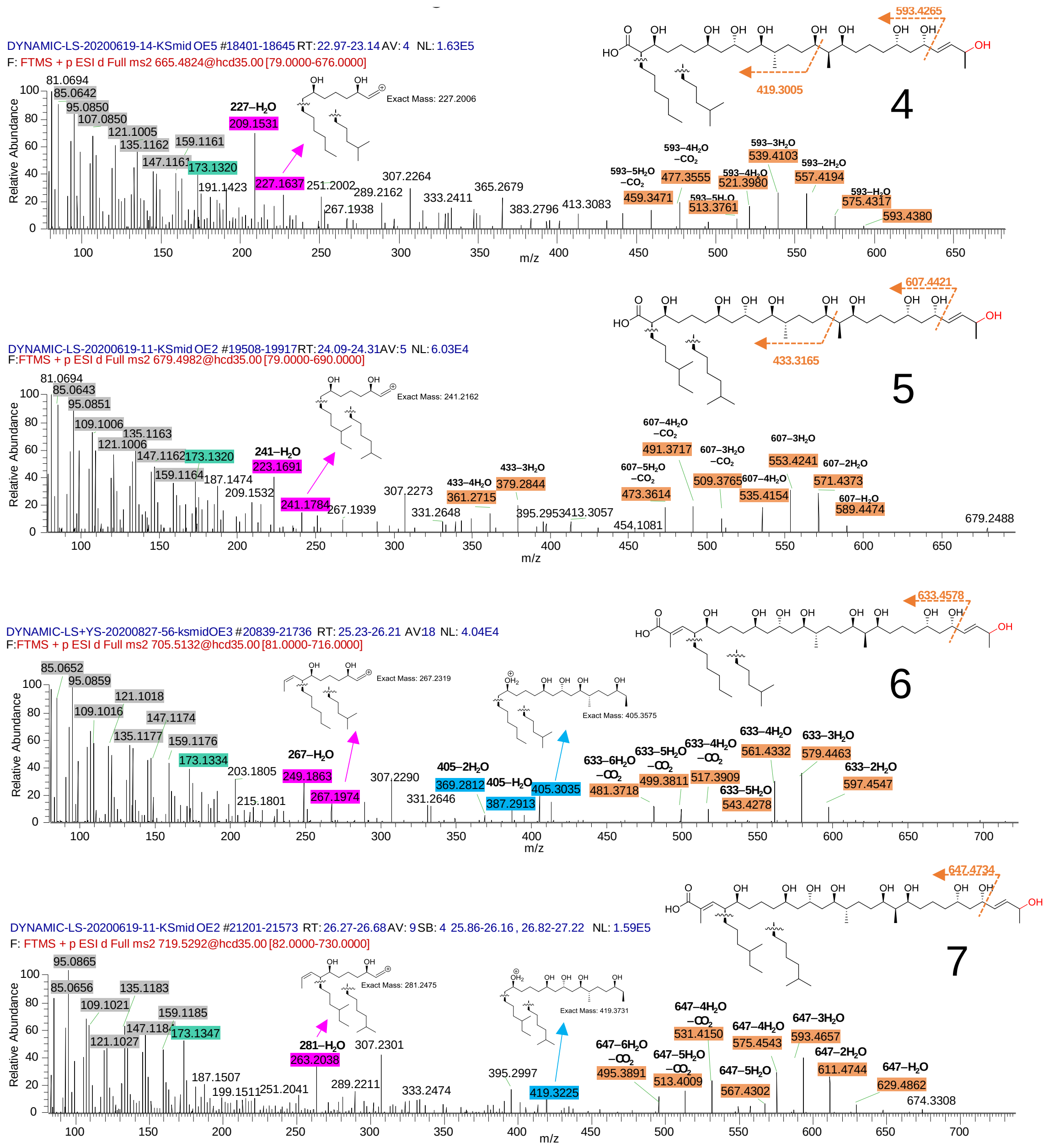

Supplementary Fig. 15. MS/MS analysis of shunt products 4–7 (represented by data from analysis of ATCC/OE484/hy59_S2). The data obtained on **4**–**7** are fully consistent with the predicted structures of intermediates released from modules 12 (**4**, **5**) and 13 (**6**, **7**). This analysis notably reveals the presence of multiple fragments common to **4**–**7** (173.13 highlighted in mint, and 159.1, 147.1, 135.1, 121.1, 109.1, 95.08 and 85.06, highlighted in grey) supporting the presence of shared structure, as well as fragment series diagnostic for the variable side chains (i.e. which differ by 14 Da between **4** and **5** and between **6** and **7**; highlighted in orange, light blue and pink). The fragments highlighted in orange also confirm the losses of water and CO_2 observed in the MS spectra.

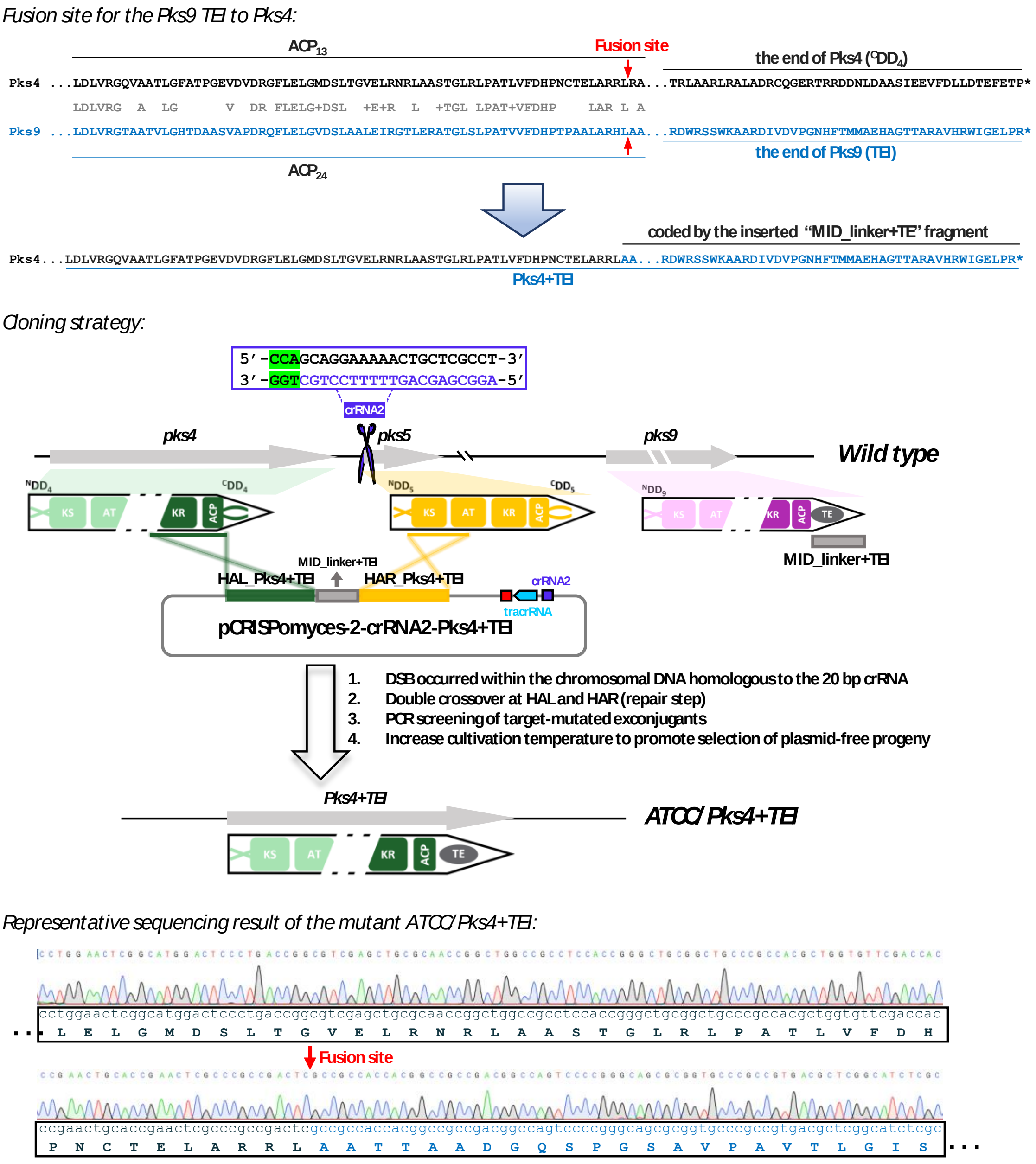

Supplementary Fig. 16. Illustration of the strategy for TE inactivation and TEI fusion. Schematic representation of the covalent fusion of the Pks9 TEI domain to Pks4 (the selected fusion site is highlighted by a red arrow). The sequence of Pks4 is in black and that of Pks9 in blue. Abbreviations: KS, ketosynthase; AT, acyl transferase; ACP, acyl carrier protein; KR, ketoreductase; TE, thioesterase; ^{C}DD, C-terminal docking domain; ^{N}DD, N-terminal docking domain.

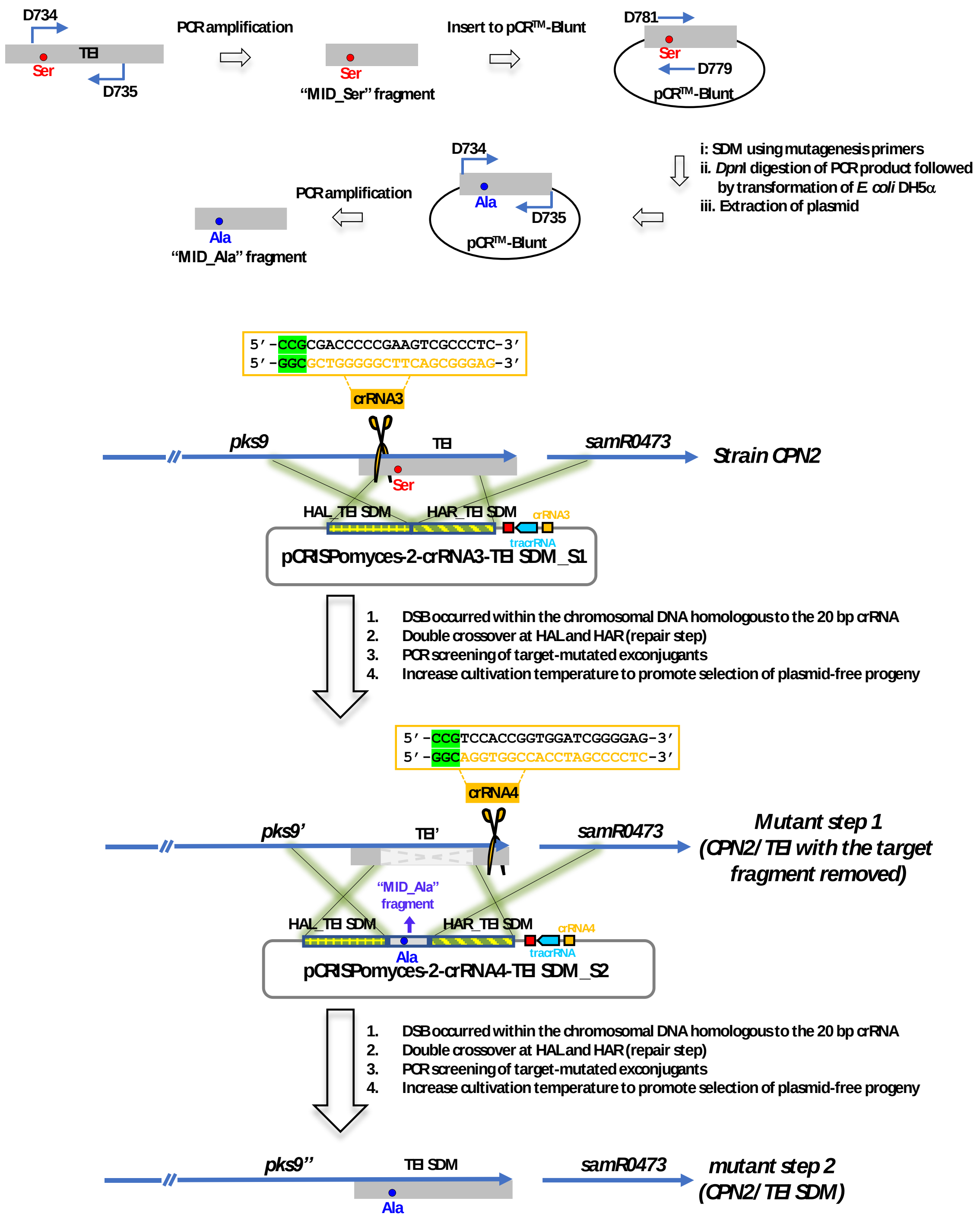
D734
TEI
Ser
D735
PCR amplification
Ser
"MID_Ser" fragment
Insert to pCR™-Blunt
D781
Ser
D779
pCR™-Blunt
i: SDM using mutagenesis primers
ii. DpnI digestion of PCR product followed by transformation of E. coli DH5α
iii. Extraction of plasmid
D734
Ala
D735
pCR™-Blunt
PCR amplification
Ala
"MID_Ala" fragment
5′-CCGCGACCCCCGAAGTCGCCCTC-3′
5′-GGCGCTGGGGGCTTCAGCGGGAG-3′
crRNA3
pks9
TEI
samR0473
Strain CPN2
Ser
HAL_TEI SDM
HAR_TEI SDM
crRNA3
tracrRNA
pCRISPomyces-2-crRNA3-TEI SDM_S1
1. DSB occurred within the chromosomal DNA homologous to the 20 bp crRNA
2. Double crossover at HAL and HAR (repair step)
3. PCR screening of target-mutated exconjugants
4. Increase cultivation temperature to promote selection of plasmid-free progeny
5′-CCGTCCACCGGTGGATCGGGGAG-3′
5′-GGCAGGTGGCCACCTAGCCCCTC-3′
crRNA4
pks9'
TEI'
samR0473
Mutant step 1
(CPN2/TEI with the target fragment removed)
"MID_Ala" fragment
HAL_TEI SDM
HAR_TEI SDM
crRNA4
Ala
tracrRNA
pCRISPomyces-2-crRNA4-TEI SDM_S2
1. DSB occurred within the chromosomal DNA homologous to the 20 bp crRNA
2. Double crossover at HAL and HAR (repair step)
3. PCR screening of target-mutated exconjugants
4. Increase cultivation temperature to promote selection of plasmid-free progeny
pks9"
TEI SDM
samR0473
mutant step 2
(CPN2/TEI SDM)
Ala

Representative sequencing result of the mutant CPN2/TEI SDM

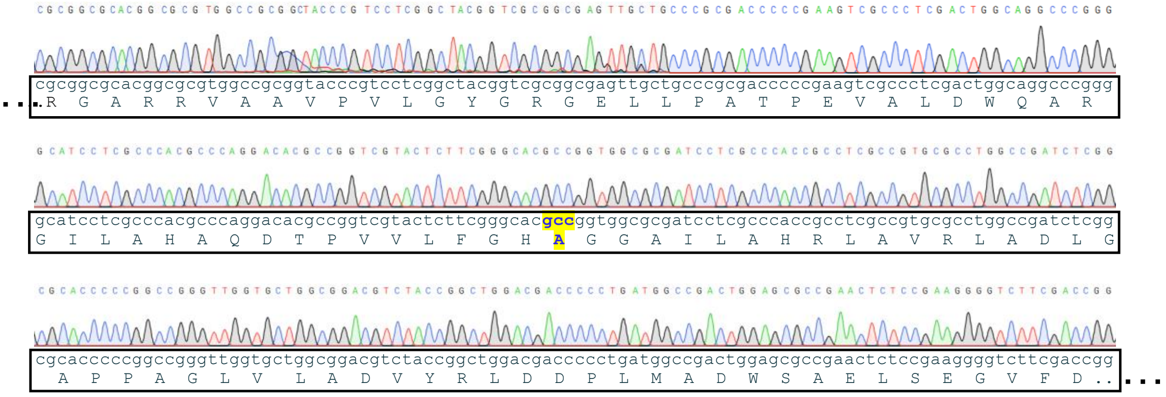

Representative sequencing result of the mutant CPN2/TEII SDM

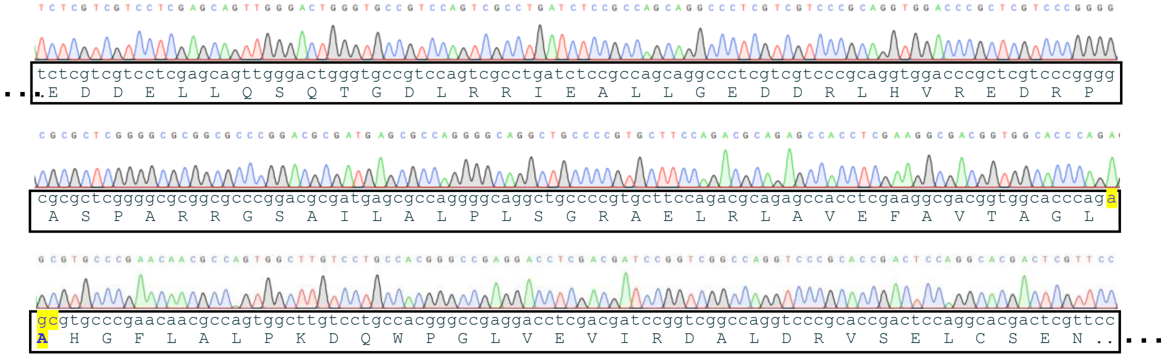

Supplementary Fig. 17. Schematic representation of TEI (and TEII) domain inactivation in strain CPN2 (represented by TEI inactivation). The catalytic Ser residue was successfully mutated to Ala (mutation verification is highlighted in yellow in the sequencing). Abbreviations: TE, thioesterase; SDM, site-directed mutagenesis.

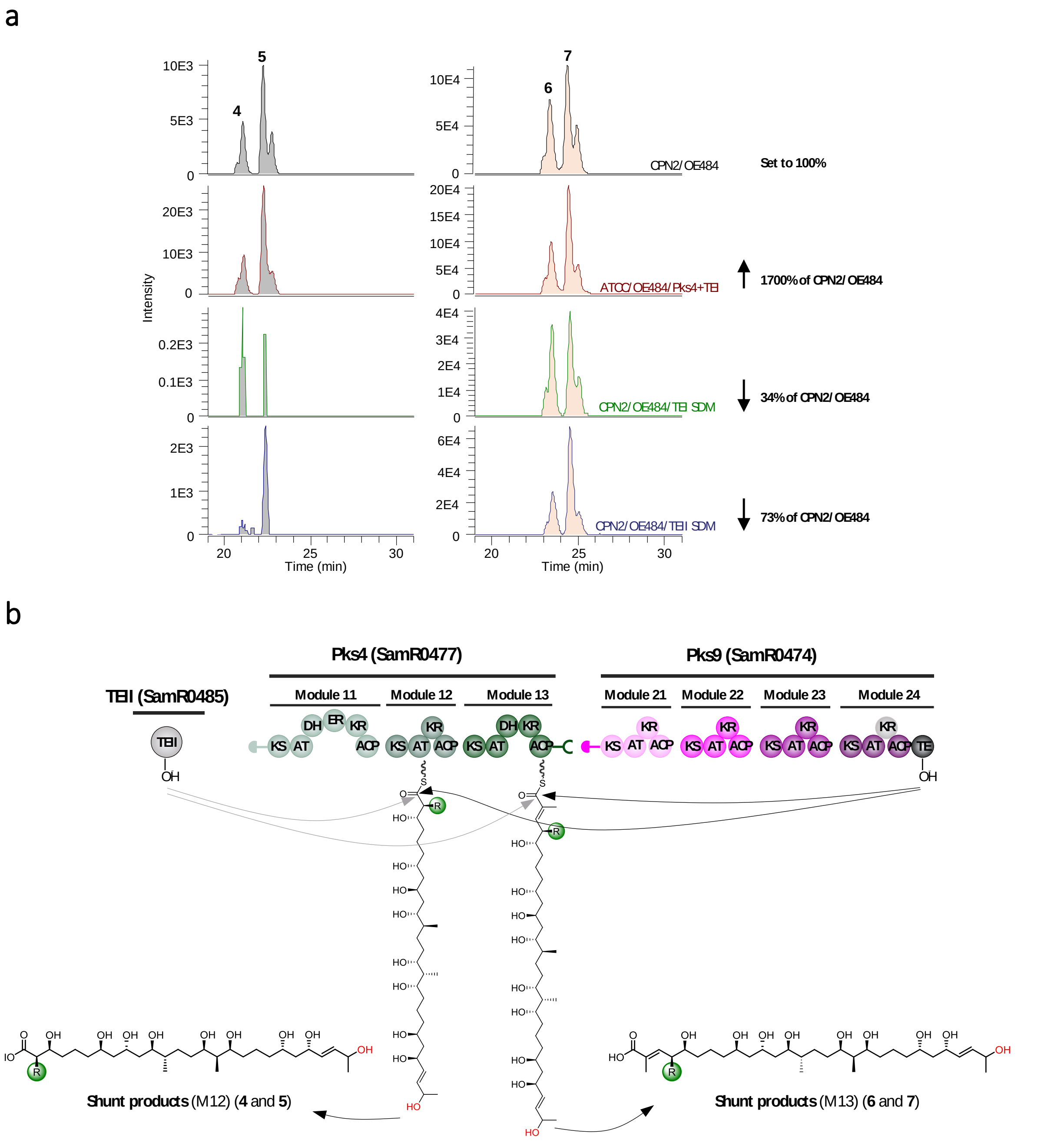

Supplementary Fig. 18. Results of the TE fusion and inactivation experiments. a HPLC-MS analysis of mutants ATCC/OE484/Pks4+TEI, CPN2/OE484/TEI SDM and CPN2/OE484/TEII SDM. Based on integrated EIC peak area (see **Supplementary Table 3**), the yields of shunt products (**4**–**7**) in mutant ATCC/Pks4+TEI were increased up to 17-fold relative to CPN2/OE484. Conversely, the inactivation of the TEI domain or TEII reduced the yield of shunt products (**4**–**7**) to 34% and 73%, respectively, relative to that of CPN2/OE484 (set to 100%). **b** Illustration of the model for the off-loading of stambomycin intermediates. A proposed interaction between TEI of Pks9 and Pks4 allows the TEI to directly off-load the immature polyketide from modules 12 and 13 to generate the shunt products (**4**–**7**). The *trans*-acting type II thioesterase (TEII), homologs of which have been shown to exhibit proof-reading activity towards inappropriately decarboxylated extender units and stalled intermediates[10], can also release the shunt products from the ACP domains. Abbreviations: KS, ketosynthase; AT, acyl transferase; ACP, acyl carrier protein; KR, ketoreductase; TE, thioesterase; ^{C}DD, C-terminal docking domain; ^{N}DD, N-terminal docking domain.

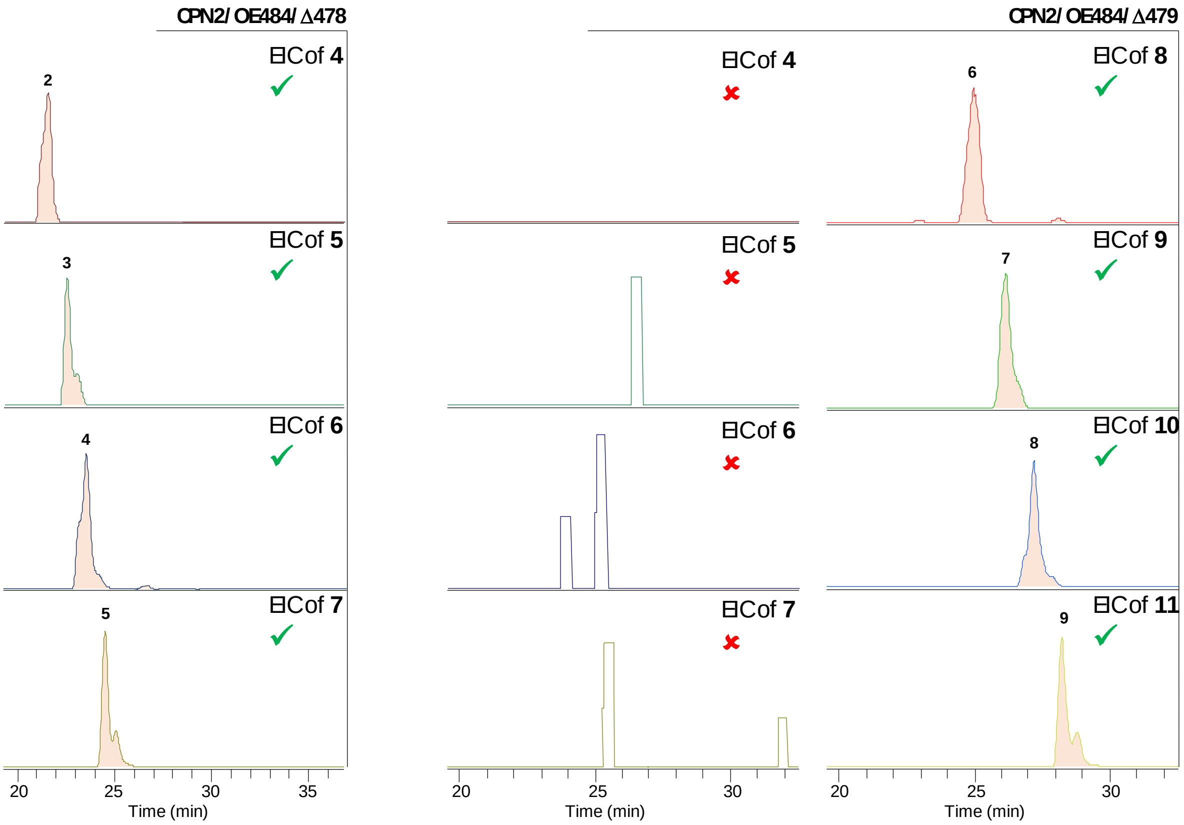

Supplementary Fig. 19. HPLC-MS analysis of inactivation of SamR0478 and SamR0479 in strain CPN2. Inactivation of SamR0478 did not change the metabolic profile compared to mutant CPN2/OE484. Inactivation of SamR0479 in mutant CPN2/OE484 disabled the production of **4**–**7** and led to deoxy shunt products **8–11**.

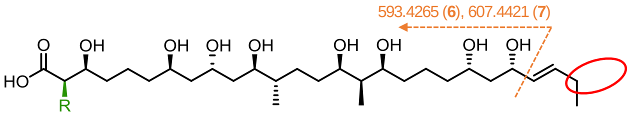

Deoxy shunt (M12) **8**

Chemical Formula: $C_{35}H_{68}O_{10}$ R = CH_2-CH_2-CH_2-$CH(CH_3)$-CH_3
CH_2-CH_2-CH_2-CH_2-CH_2-CH_3

$[M+H]^+$: $C_{35}H_{69}O_{10}^+$, Calc'd: 649.4889, Mass error = –4.8 ppm

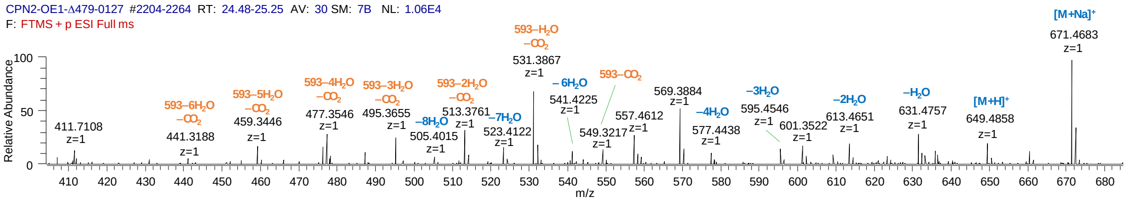

Deoxy shunt (M12) **9**

Chemical Formula: $C_{36}H_{70}O_{10}$ R = CH_2-CH_2-CH_2-$CH(CH_3)$ -CH_2-CH_3
CH_2-CH_2-CH_2-CH_2-CH (CH_3) -CH_3

$[M+H]^+$: $C_{36}H_{71}O_{10}^+$, Calc'd: 663.5051, Mass error = –5.0 ppm

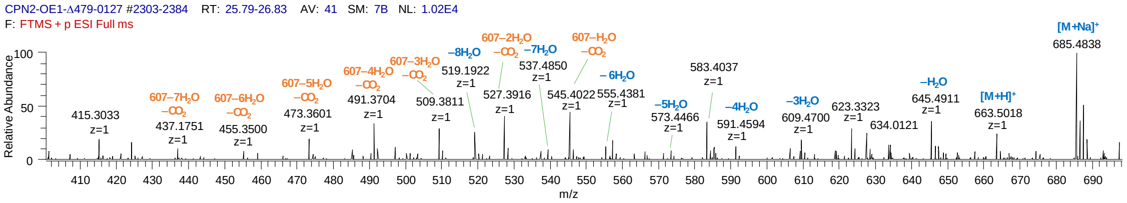

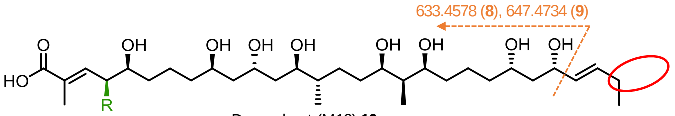

Deoxy shunt (M13) **10**

Chemical Formula: $C_{38}H_{72}O_{10}$ R = CH_2-CH_2-CH_2-$CH(CH_3)$-CH_3
CH_2-CH_2-CH_2-CH_2-CH_2-CH_3

$[M+H]^+$: $C_{38}H_{73}O_{10}^+$, Calc'd: 689.5198, Mass error = –3.0 ppm

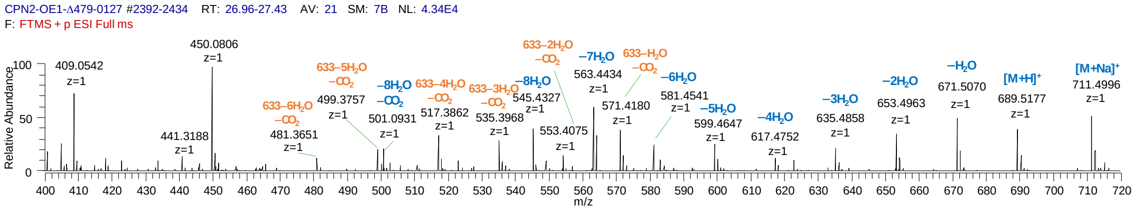

Deoxy shunt (M13) **11**

Chemical Formula: $C_{39}H_{74}O_{10}$ R = CH_2-CH_2-CH_2-$CH(CH_3)$ -CH_2-CH_3
CH_2-CH_2-CH_2-CH_2-CH (CH_3) -CH_3

$[M+H]^+$: $C_{39}H_{75}O_{10}^+$, Calc'd: 703.5355, Mass error = –3.0 ppm

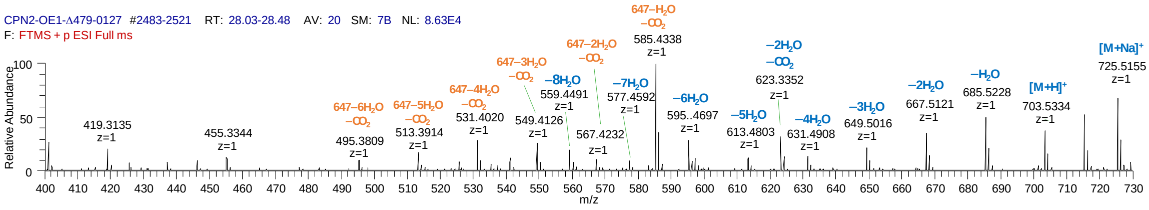

Supplementary Fig. 20. MS analysis of shunt products 8–11**.** Mass spectra of the deoxy shunt products. The red circle on the structures indicates the missing terminal hydroxyl group. This analysis provides multiple lines of evidence supporting our structural assignments: i) the presence of both C/D (**8**, **10**) and A/B (**9**, **11**) deoxy analogues released from modules 12 and 13, respectively; ii) peaks in the MS spectra corresponding to loss of 8 hydroxyl groups in the molecules as water (as opposed to 9 from metabolites **4**–**7**); and iii) multiple peaks corresponding to loss of the terminal CO_2 group, consistent with linear structures.

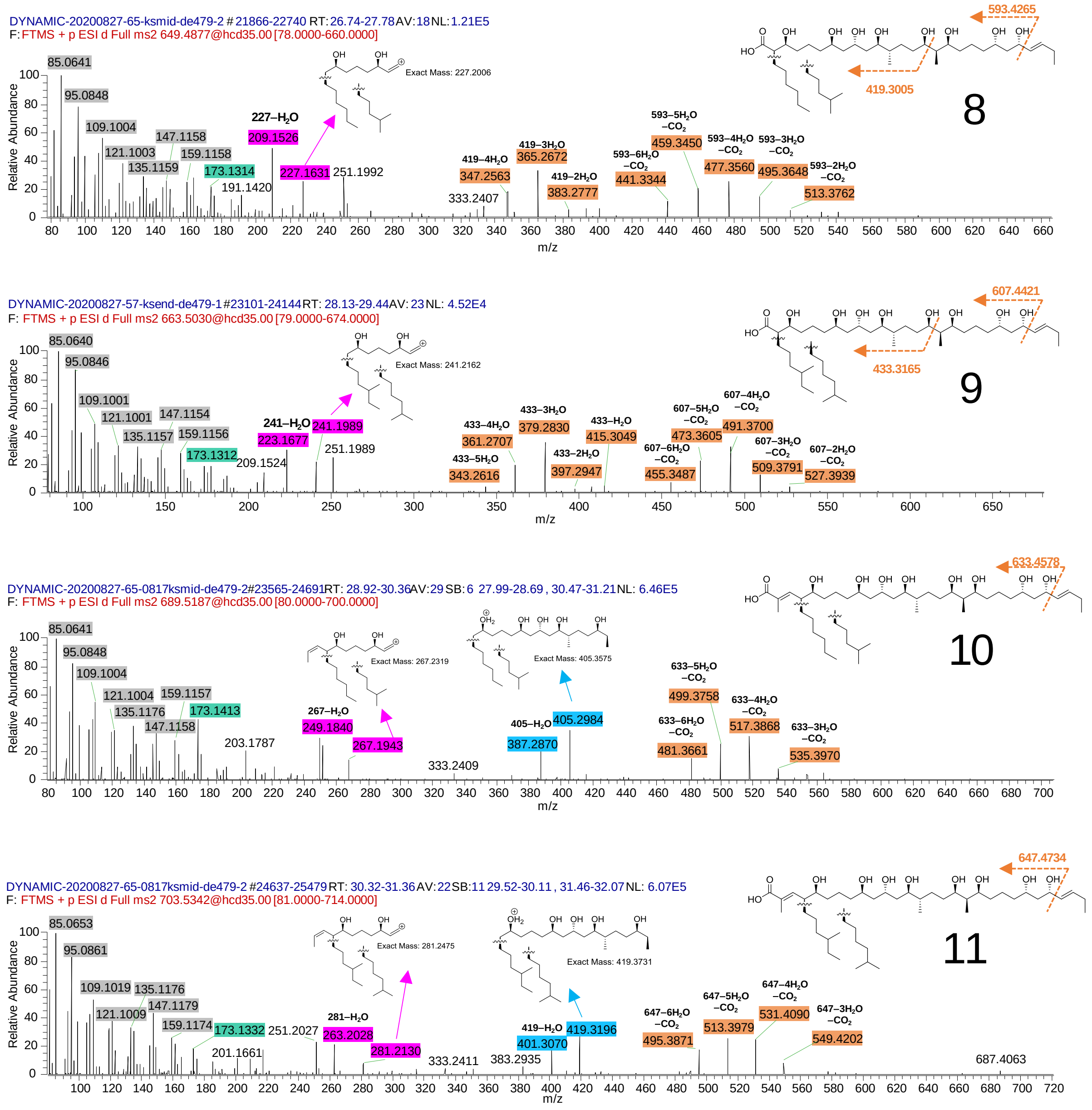

Supplementary Fig. 21. MS/MS analysis of shunt products 8–11. MS[2] spectra of the shunt products **8**–**11** (represented by data from analysis of ATCC/OE484/hy59_S2/Δ479). The data obtained on **8**–**11** are fully consistent with the predicted structures of deoxy versions of **4**–**7** released from modules 12 (**8**, **9**) and 13 (**10**, **11**). This analysis notably reveals the presence of multiple fragments common to **8**–**11** (173.13 highlighted in mint, and 159.1, 147.1, 135.1, 121.1, 109.1, 95.08 and 85.06 highlighted in grey) and which they share with metabolites **4**–**7**, as well as fragment series diagnostic for the variable side chains (i.e. which differ by 14 Da between **8** and **9** and between **10** and **11**; highlighted in orange, light blue and pink). The fragments highlighted in orange also confirm the losses of water and CO_2 observed in the MS spectra.

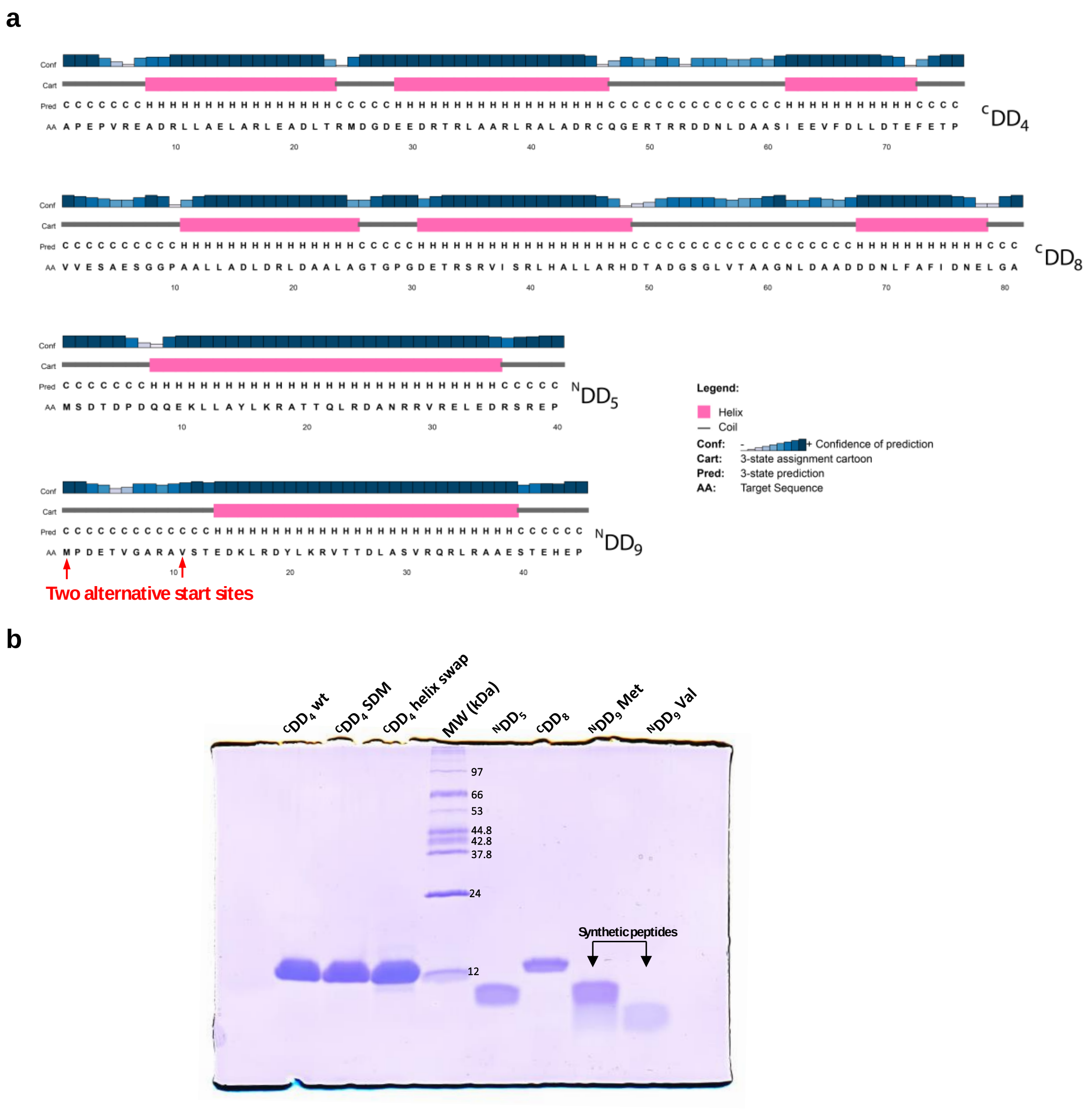

Supplementary Fig. 22. Secondary structure prediction and expression/purification of the docking domains. a PSIPRED graphical output of secondary structure prediction on docking domains. Based on this analysis, we identified an extended random coil region in $^{N}DD_9$ upstream of the predicted coiled-coil structure, which prompted us to speculate that the Val (Val-Ser-Thr…) is likely to be the true start site. **b** SDS-PAGE analysis of docking domains (both recombinant and commercially-synthesized) used in this work. The purifications were carried out once each, as sufficient material was obtained. Abbreviations: ^{C}DD, C-terminal docking domain; ^{N}DD, N-terminal docking domain. Source data are provided as a Source Data file.

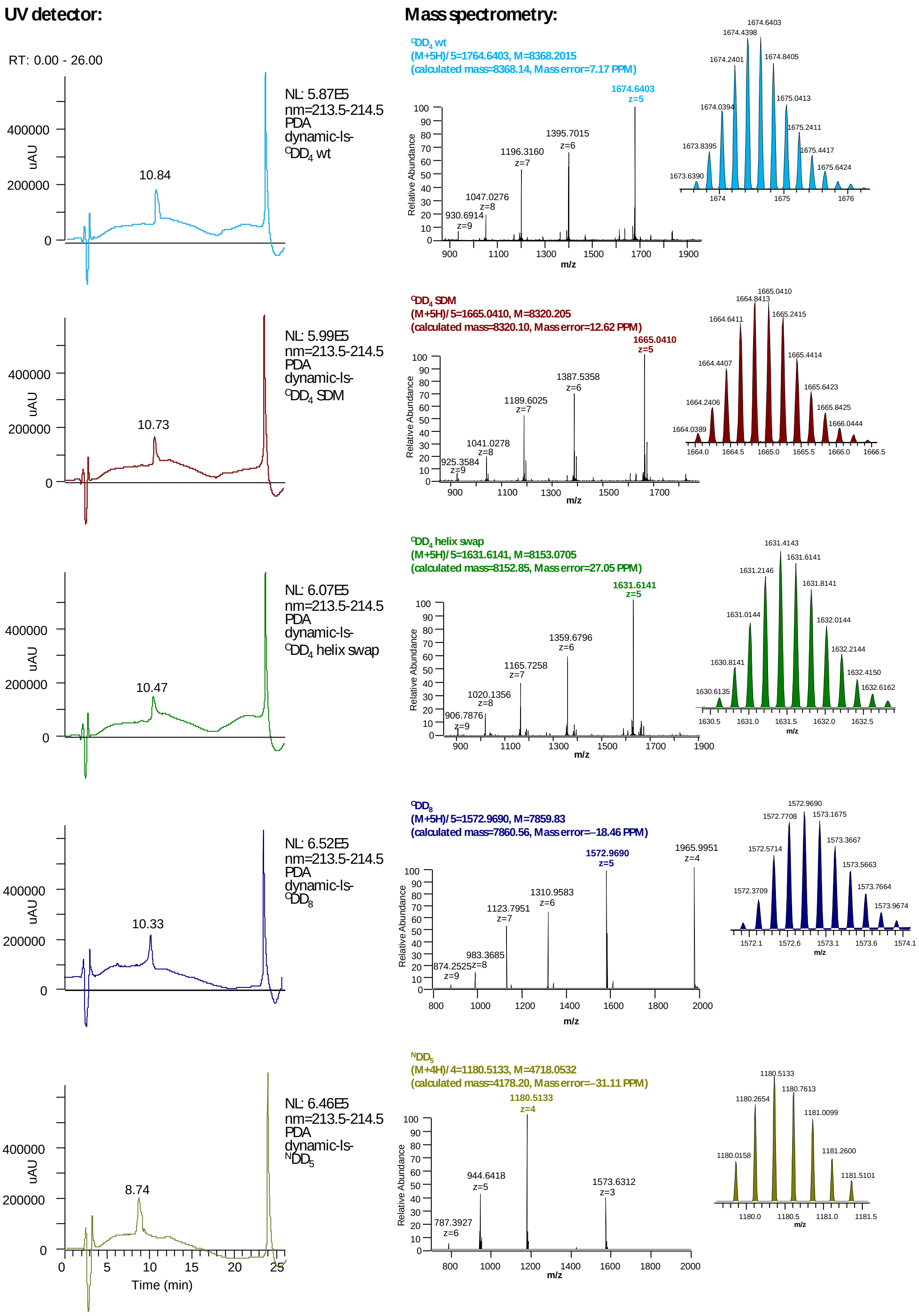

Supplementary Fig. 23. HPLC and mass spectrometry analysis of recombinant docking domains ($^{C}DD_4$ wt, $^{C}DD_4$ SDM, $^{C}DD_4$ helix swap, $^{N}DD_5$, and $^{C}DD_8$). Abbreviations: ^{C}DD, C-terminal docking domain; ^{N}DD, N-terminal docking domain.

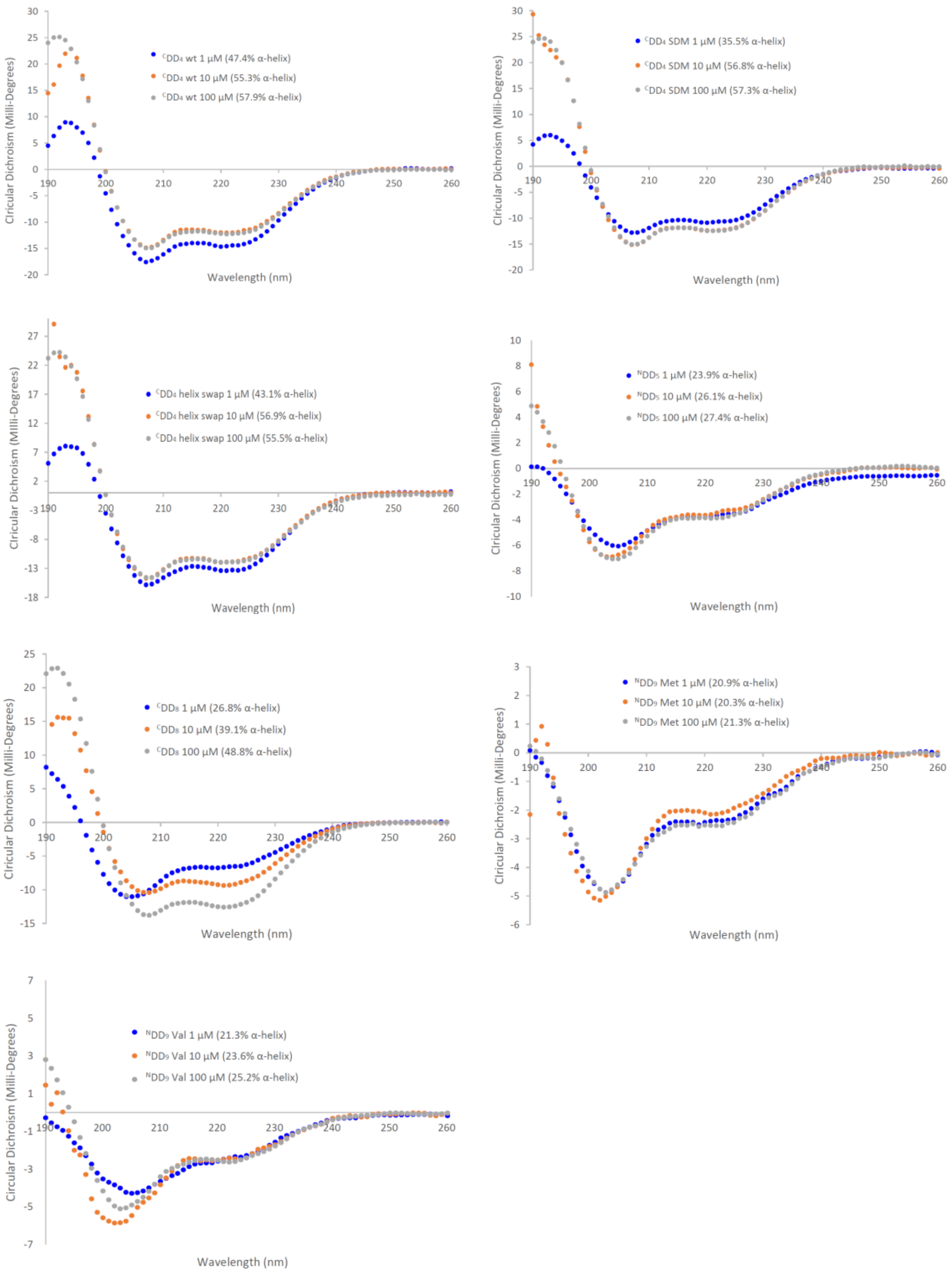

Supplementary Fig. 24. Circular dichroism analysis of all docking domains. The analysis was performed at concentrations of 100, 10 and 1 μM. The percentage α-helices in each case was calculated using deconvolution software CDNN2.1[11]. Abbreviations: ^{C}DD, C-terminal docking domain; ^{N}DD, N-terminal docking domain. Source data are provided as a Source Data file.

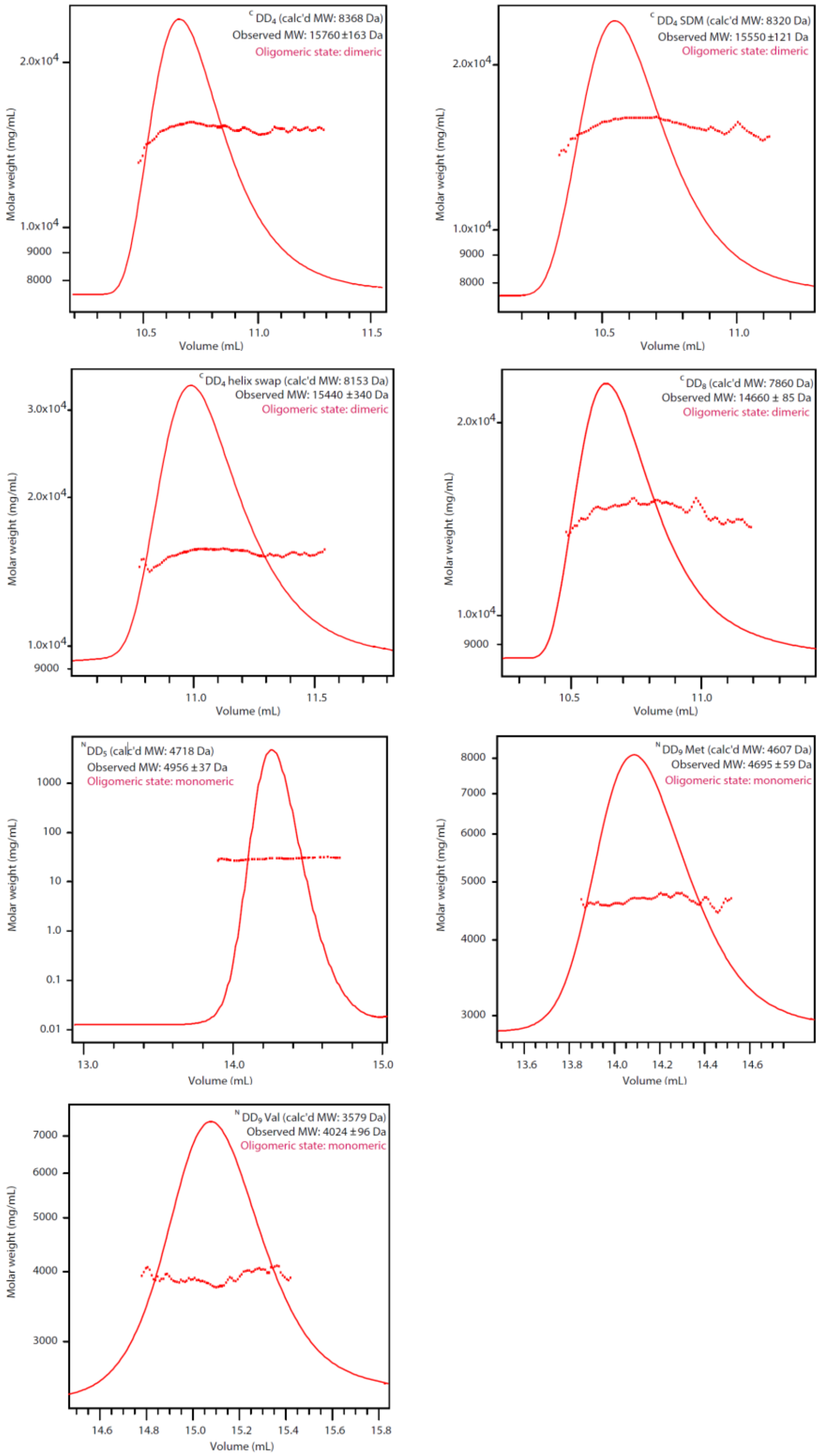

Supplementary Fig. 25. SEC-MALS analysis of the docking domains. Comparison of the observed molecular weights (MW) and the calculated molecular weights (in brackets) shows that all ^{C}DDs are in dimeric in solution and the ^{N}DDs are uniformly monomer.ric. Abbreviations: ^{C}DD, C-terminal docking domain; ^{N}DD, N-terminal docking domain.

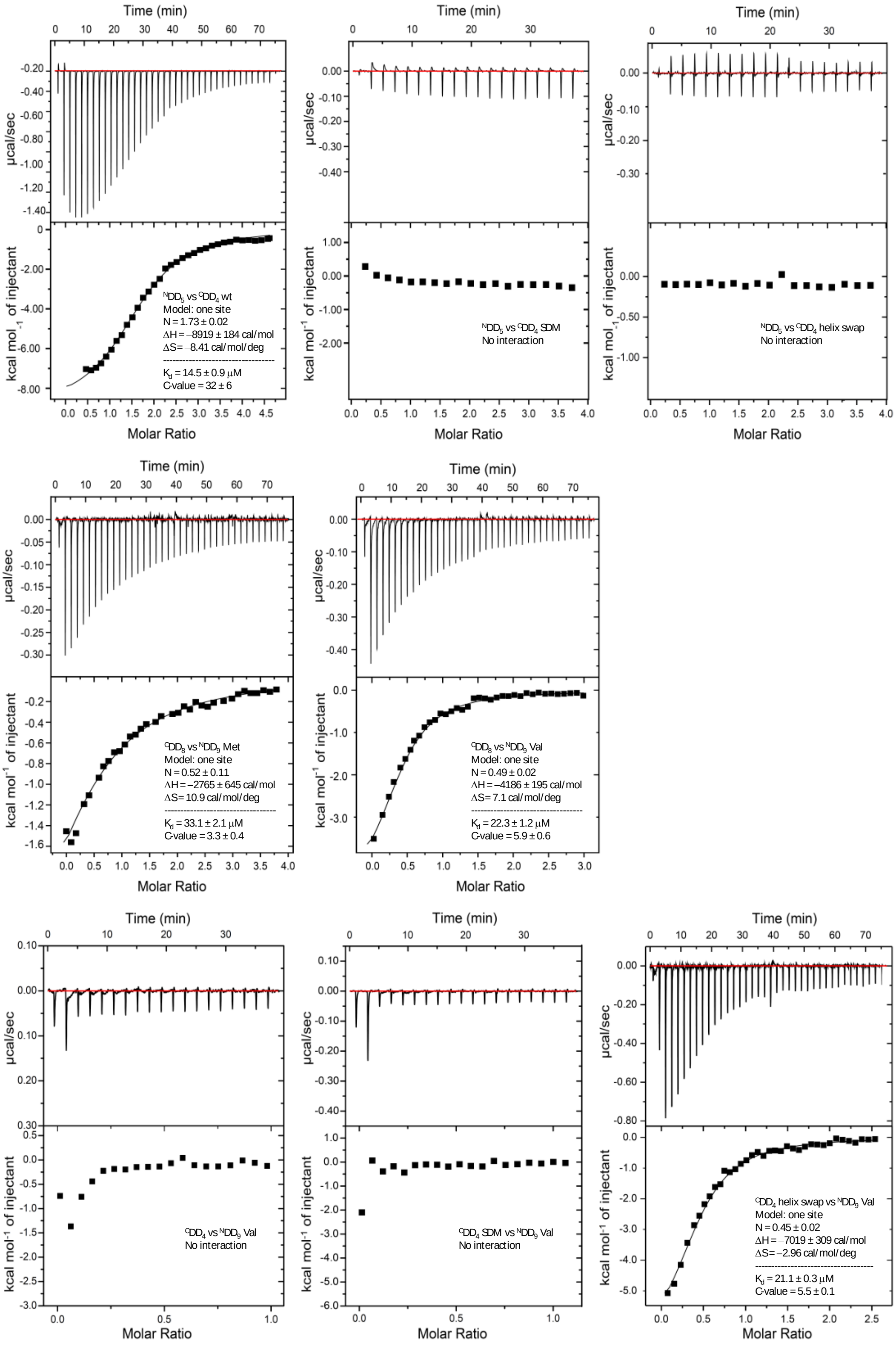

Supplementary Fig. 26. ITC analysis of binding between pairs of ^{C}DDs and ^{N}DDs. Shown are the ITC thermograms and the calculated binding affinities. One set of representative data (from two replicate experiments) is shown, but the K_d and C-values represent the average of the two measurements. Abbreviations: ^{C}DD, C-terminal docking domain; ^{N}DD, N-terminal docking domain. Source data are provided as a Source Data file.

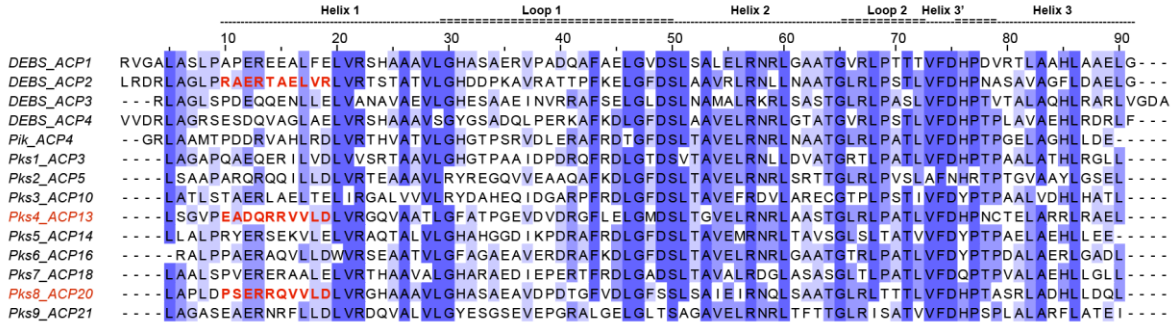

Supplementary Fig. 27. Multiple sequence alignment of stambomycin ACP domains to identify the residues/motifs putatively mediating interaction with the down- and upstream KS domains. Assignment of secondary structure and residue numbering is based on the solved NMR structure of DEBS ACP_2 (PDB: 2JU2[12]). Serine 50 is the conserved residue that serves as the attachment point for the phosphopantetheine prosthetic group. Residues 10−19 on helix α1 (H1) (in red) were identified as involved in the ACP/KS intermodular chain translocation[13], while residues 31−54 located on loop 1 and helix α2 (L1+H2 region) (in yellow) were found to contribute to ACP/KS communication during intramodular chain elongation[13]. The bold italic RR on loop 1 of DEBS ACP_3 represents two positions that were identified previously to be most critical to the interaction[14]; the corresponding residues on ACP_{14} and ACP_{21} are DR and GR, respectively. Abbreviations: DEBS, 6-deoxyerythronolide B synthase; Pik, pikromycin synthase.

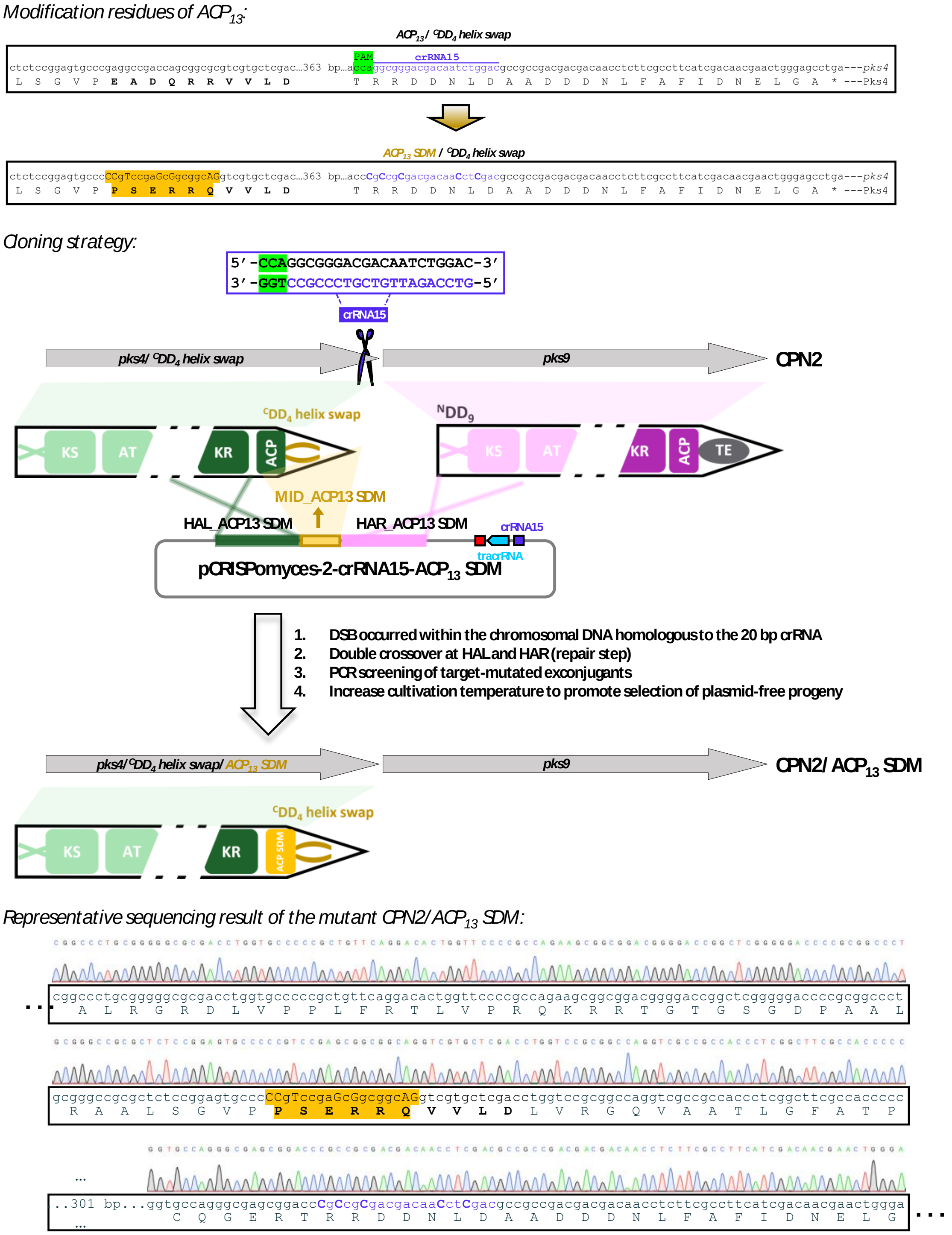

Supplementary Fig. 28. Illustration of the strategy to modify ACP_{13} in mutant CPN2. The first 10 residues of helix α1 on ACP_{13} were mutated from EADQRRVVLD to PSERRQVVLD (highlighted in orange). In addition, the DNA sequence corresponding to the selected crRNA15 was also mutated (as shown in blue capital letters) to avoid unwanted cleavage by Cas9 within the genome of the newly-obtained mutant.

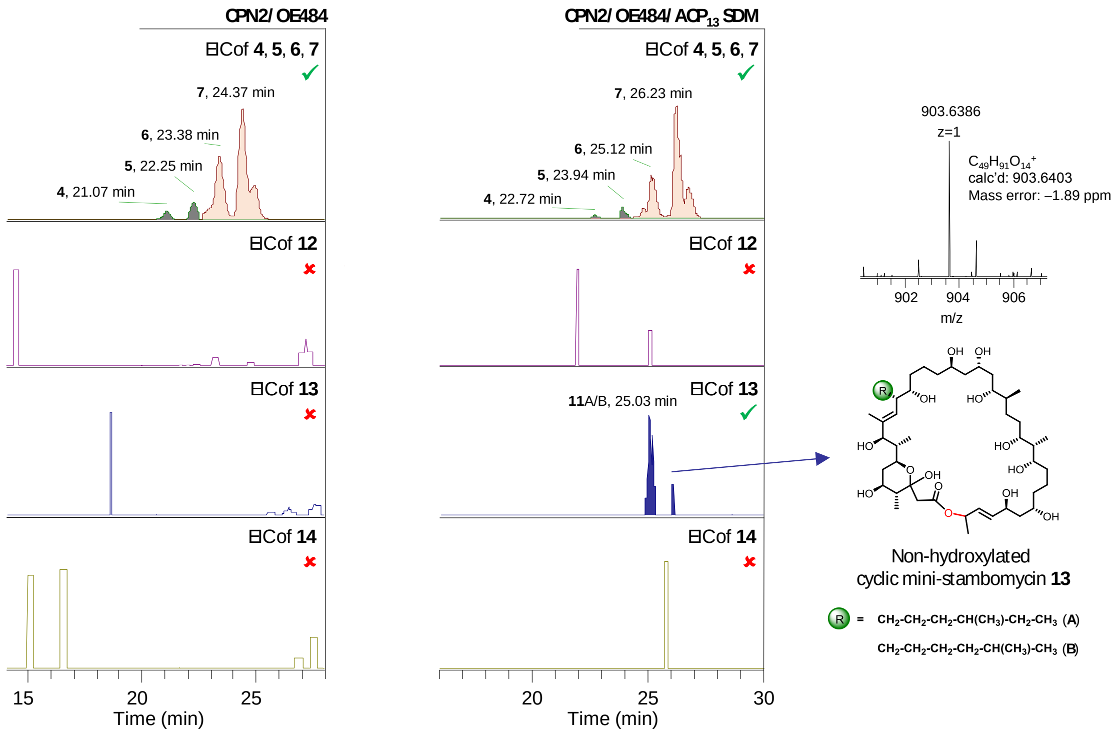

Supplementary Fig. 29. HPLC-MS analysis of the mutant CPN2/ACP$_{13}$ SDM. An EIC peak with the mass of 903.6386 ([M+H]$^+$), corresponding to the cyclic mini-stambomycins A/B **13** lacking the hydroxyl introduced by SamR0478, was detected successfully (see **Supplementary Figs. 33** and **34** for all data supporting the structure assignment). Note: the discrepancy in retention times for shunt metabolites **4**–**7** between the mutants CPN2/OE484 and CPN2/OE484/ACP$_{13}$ SDM reflects the fact that the analyses were carried out on two different machines, a UHPLC-LTQ Orbitrap and a UHPLC-Orbitrap ID-X Tribrid (indeed, reinjection of the extract of CPN2/OE484 on the UHPLC-Orbitrap ID-X Tribrid yielded identical retention times for the set of compounds). The same explanation applies to **Supplementary Figs. 32** and **40**. Abbreviations: KS, ketosynthase; AT, acyl transferase; ACP, acyl carrier protein; KR, ketoreductase; TE, thioesterase; ^{C}DD, C-terminal docking domain; ^{N}DD, N-terminal docking domain.

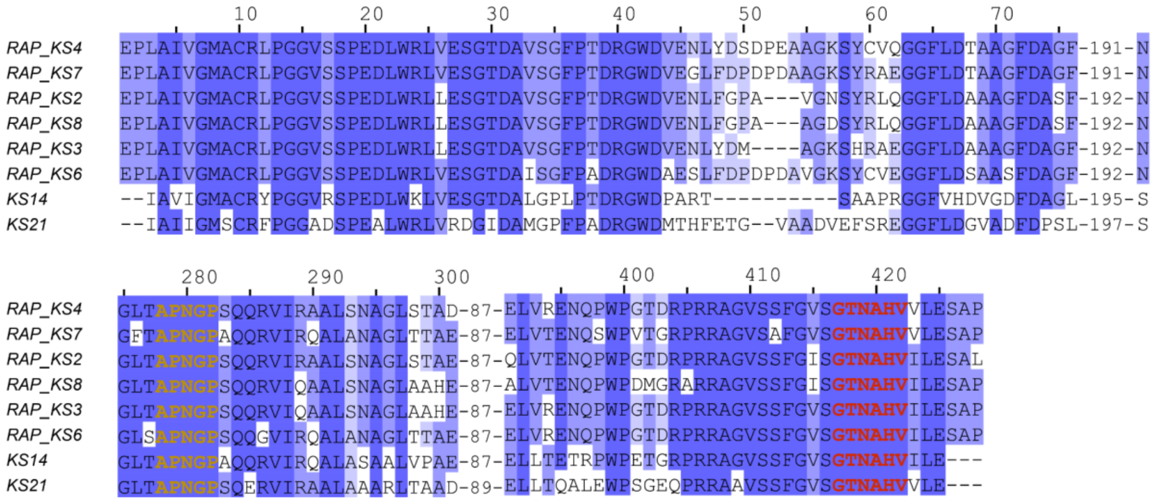

Supplementary Fig. 30. Sequence alignment of KS_{14} and KS_{21} with the KS domains from the rapamycin PKS, highlighting the two alternative conserved sequence motifs that were exploited in our experiments. Motif APNGP (in orange) is located near the middle of the KS domains and corresponds to a recombination hot spot identified during induced evolution of the rapamycin PKS[15]. Motif GTNAHV (in red), at the C-terminal end of the KS domains, is a site identified empirically by genetic engineering that allows for efficient AT swapping[16]. Abbreviations: KS, ketosynthase; RAP, rapamycin synthase.

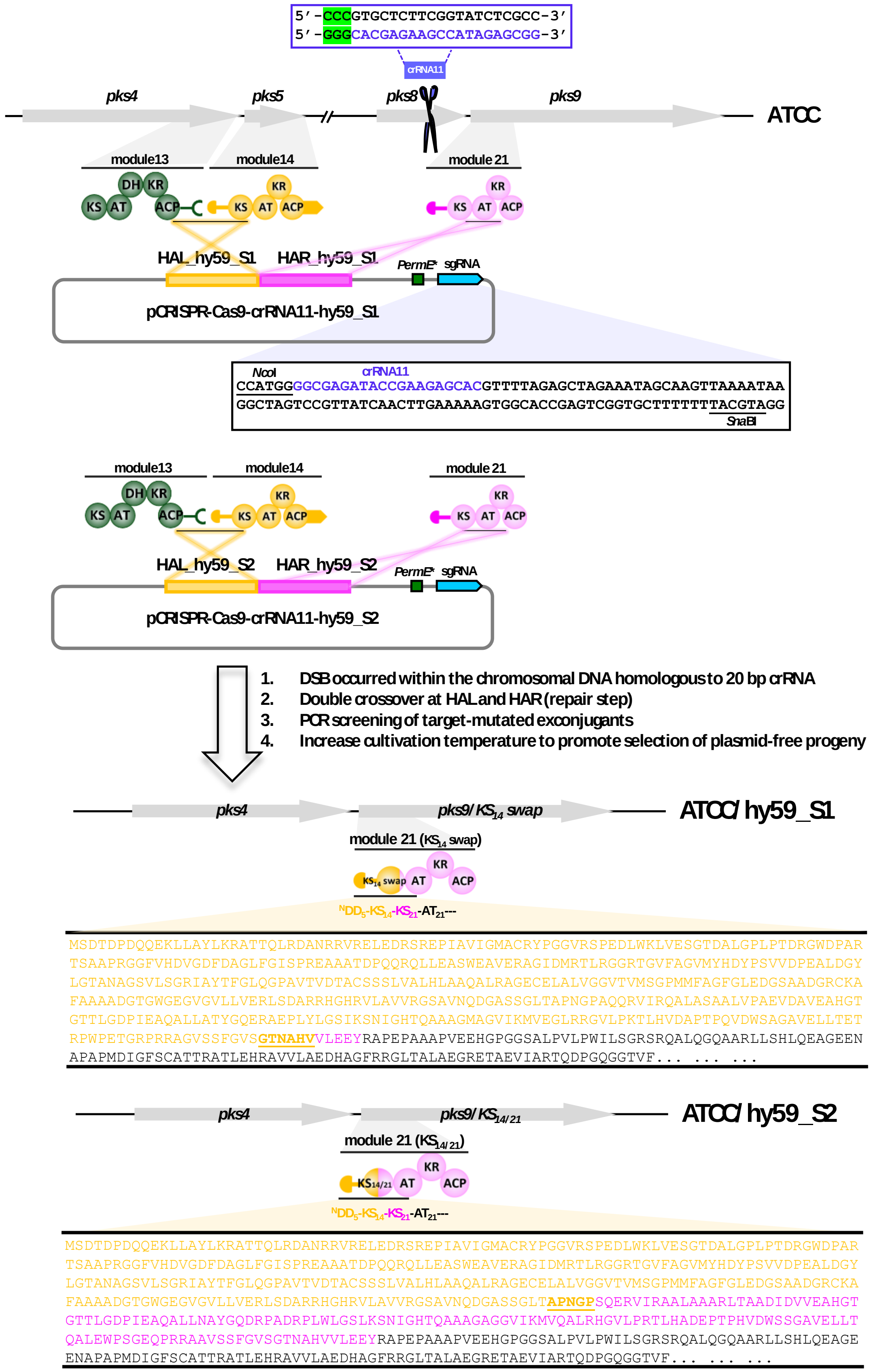

5′-CCCGTGCTCTTCGGTATCTCGCC-3′
5′-GGGCACGAGAAGCCATAGAGCGG-3′
crRNA11
pks4
pks5
pks8
pks9
ATCC
module13
module14
module 21
DH KR
KS AT ACP
KR
KS AT ACP
KR
KS AT ACP
HAL_hy59_S1
HAR_hy59_S1
PermE*
sgRNA
pCRISPR-Cas9-crRNA11-hy59_S1
NcoI
crRNA11
CCATGGGGCGAGATACCGAAGAGCACGTTTTAGAGCTAGAAATAGCAAGTTAAAATAA
GGCTAGTCCGTTATCAACTTGAAAAAGTGGCACCGAGTCGGTGCTTTTTTTACGTAGG
SnaBI
module13
module14
module 21
HAL_hy59_S2
HAR_hy59_S2
PermE*
sgRNA
pCRISPR-Cas9-crRNA11-hy59_S2
1. DSB occurred within the chromosomal DNA homologous to 20 bp crRNA
2. Double crossover at HAL and HAR (repair step)
3. PCR screening of target-mutated exconjugants
4. Increase cultivation temperature to promote selection of plasmid-free progeny
pks4
pks9/KS14 swap
ATCC/hy59_S1
module 21 (KS14 swap)
KS14 swap AT KR ACP
NDD5-KS14-KS21-AT21---
MSDTDPDQQEKLLAYLKRATTQLRDANRRVRELEDRSREPIAVIGMACRYPGGVRSPEDLWKLVESGTDALGPLPTDRGWDPAR
TSAAPRGGFVHDVGDFDAGLFGISPREAAATDPQQRQLLEASWEAVERAGIDMRTLRGGRTGVFAGVMYHDYPSVVDPEALDGY
LGTANAGSVLSGRIAYTFGLQGPAVTVDTACSSSLVALHLAAQALRAGECELALVGGVTVMSGPMMFAGFGLEDGSAADGRCKA
FAAAADGTGWGEGVGVLLVERLSDARRHGHRVLAVVRGSAVNQDGASSGLTAPNGPAQQRVIRQALASAALVPAEVDAVEAHGT
GTTLGDPIEAQALLATYGQERAEPLYLGSIKSNIGHTQAAAGMAGVIKMVEGLRRGVLPKTLHVDAPTPQVDWSAGAVELLTET
RPWPETGRPRRAGVSSFGVSGTNAHVVLEEYRAPEPAAAPVEEHGPGGSALPVLPWILSGRSRQALQGQAARLLSHLQEAGEEN
APAPMDIGFSCATTRATLEHRAVVLAEDHAGFRRGLTALAEGRETAEVIARTQDPGQGGTVF... ... ...
pks4
pks9/KS14/21
ATCC/hy59_S2
module 21 (KS14/21)
KS14/21 AT KR ACP
NDD5-KS14-KS21-AT21---
MSDTDPDQQEKLLAYLKRATTQLRDANRRVRELEDRSREPIAVIGMACRYPGGVRSPEDLWKLVESGTDALGPLPTDRGWDPAR
TSAAPRGGFVHDVGDFDAGLFGISPREAAATDPQQRQLLEASWEAVERAGIDMRTLRGGRTGVFAGVMYHDYPSVVDPEALDGY
LGTANAGSVLSGRIAYTFGLQGPAVTVDTACSSSLVALHLAAQALRAGECELALVGGVTVMSGPMMFAGFGLEDGSAADGRCKA
FAAAADGTGWGEGVGVLLVERLSDARRHGHRVLAVVRGSAVNQDGASSGLTAPNGPSQERVIRAALAAARLTAADIDVVEAHGT
GTTLGDPIEAQALLNAYGQDRPADRPLWLGSLKSNIGHTQAAAGAGGVIKMVQALRHGVLPRTLHADEPTPHVDWSSGAVELLT
QALEWPSGEQPRRAAVSSFGVSGTNAHVVLEEYRAPEPAAAPVEEHGPGGSALPVLPWILSGRSRQALQGQAARLLSHLQEAGE
ENAPAPMDIGFSCATTRATLEHRAVVLAEDHAGFRRGLTALAEGRETAEVIARTQDPGQGGTVF... ... ...

Representative sequencing result of the mutant ATCC/hy59_S1 (KS$_{14}$ swap):

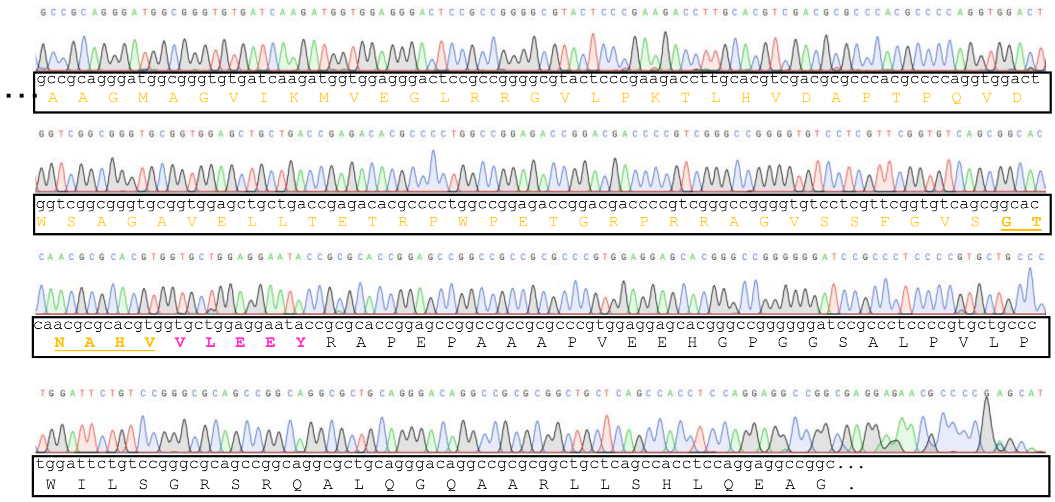

Representative sequencing result of the mutant ATCC/hy59_S2 (KS$_{14/21}$):

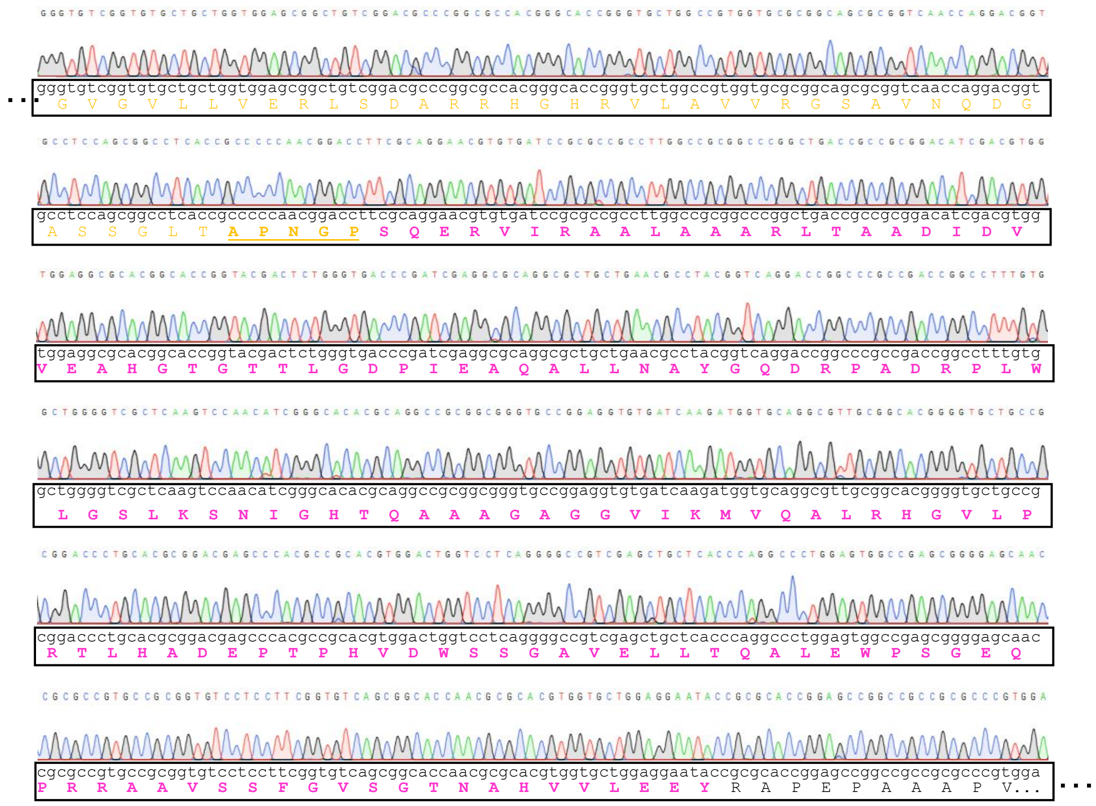

Supplementary Fig. 31. Illustration of the strategies to swap KS$_{21}$ with KS$_{14}$, and to generate a hybrid KS$_{14/21}$. In the case of mutant ATCC/hy59_S1, only five residues of KS$_{21}$ were retained, while for mutant ATCC/hy59_S2, the N-terminal docking domain of subunit 9 (^{N}DD$_{9}$) and half of KS$_{21}$ were replaced by corresponding regions of Pks5. Also shown are representative sequencing data used to verify the accuracy of the two constructs. Amino acids in orange derive from Pks5, while those in pink and black (KS-AT linker), derive from Pks9. Abbreviations: KS, ketosynthase; AT, acyl transferase; ACP, acyl carrier protein; DH, dehydratase; ER, enoyl reductase; KR, ketoreductase; ^{C}DD, C-terminal docking domain; ^{N}DD, N-terminal docking domain.

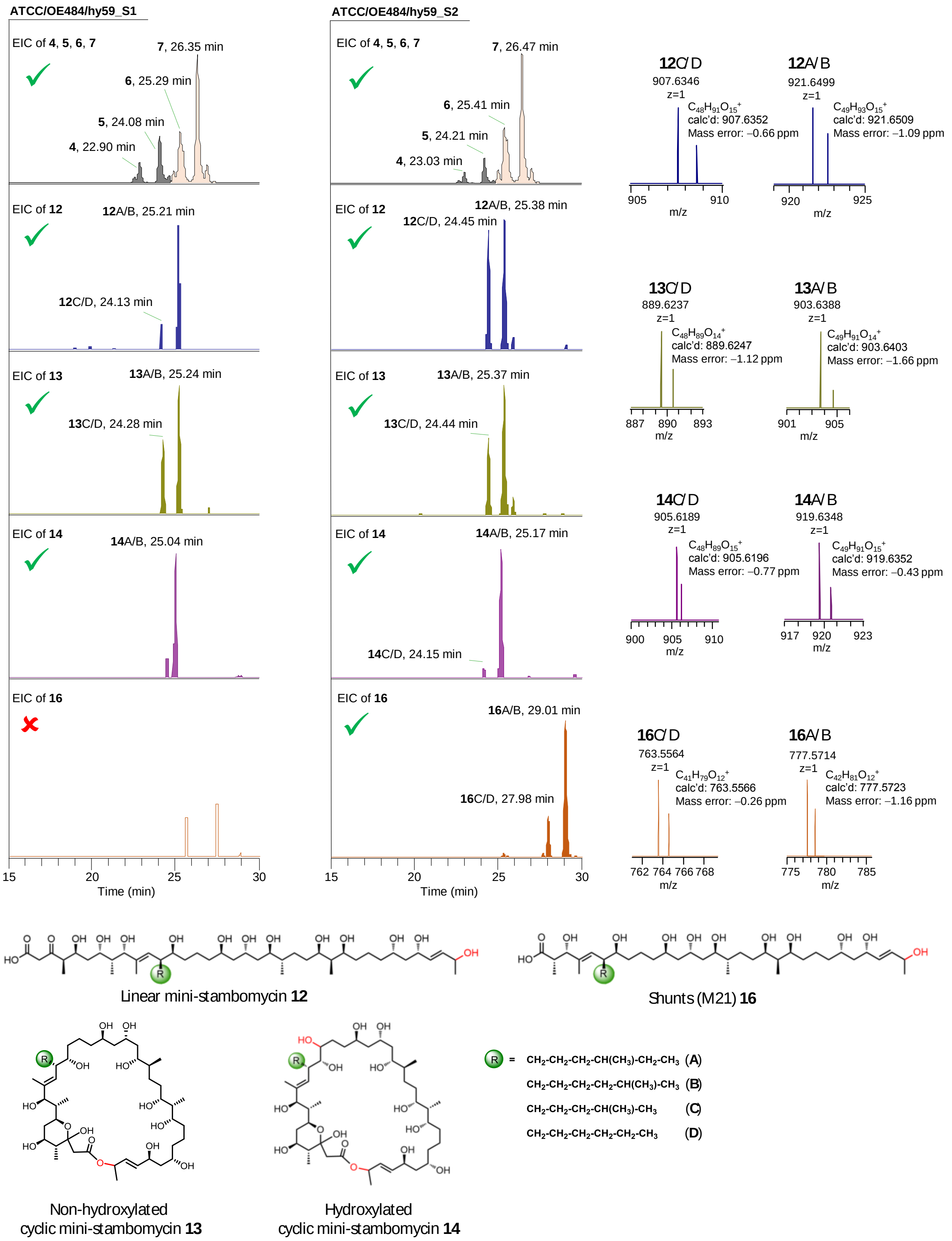

Supplementary Fig. 32. Comparative HPLC-MS analysis of mutants ATCC/OE484/hy59_S1 and ATCC/OE484/hy59_S2. The structures of the obtained metabolites are shown (which vary in the nature of the R group), with the two cytochrome P450-catalyzed hydroxylations indicated in red. M21 refers to the module from which the intermediate was released.

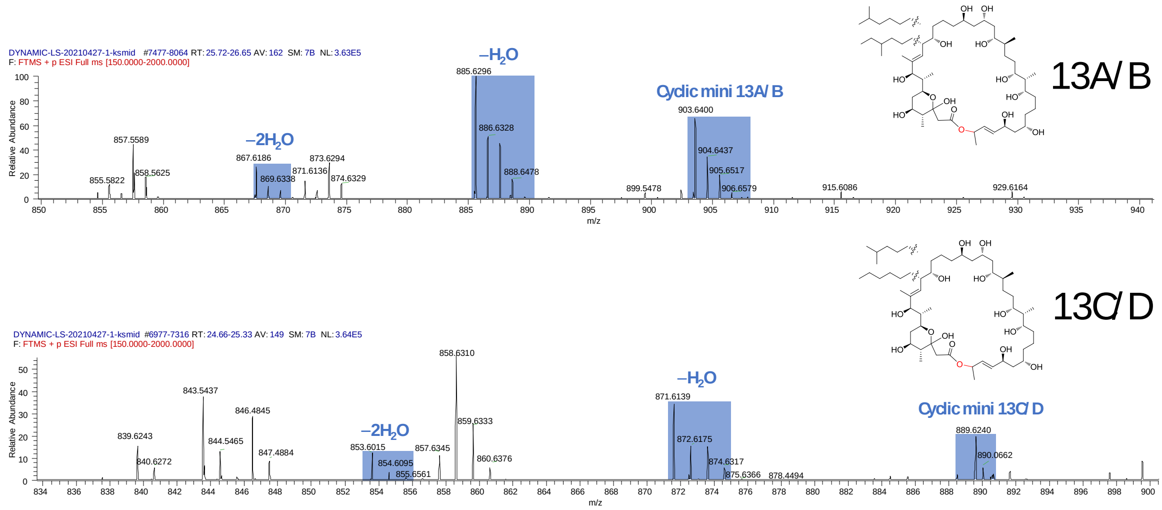

Supplementary Fig. 33. MS analysis of non-hydroxylated cyclic mini-stambomycins 13.

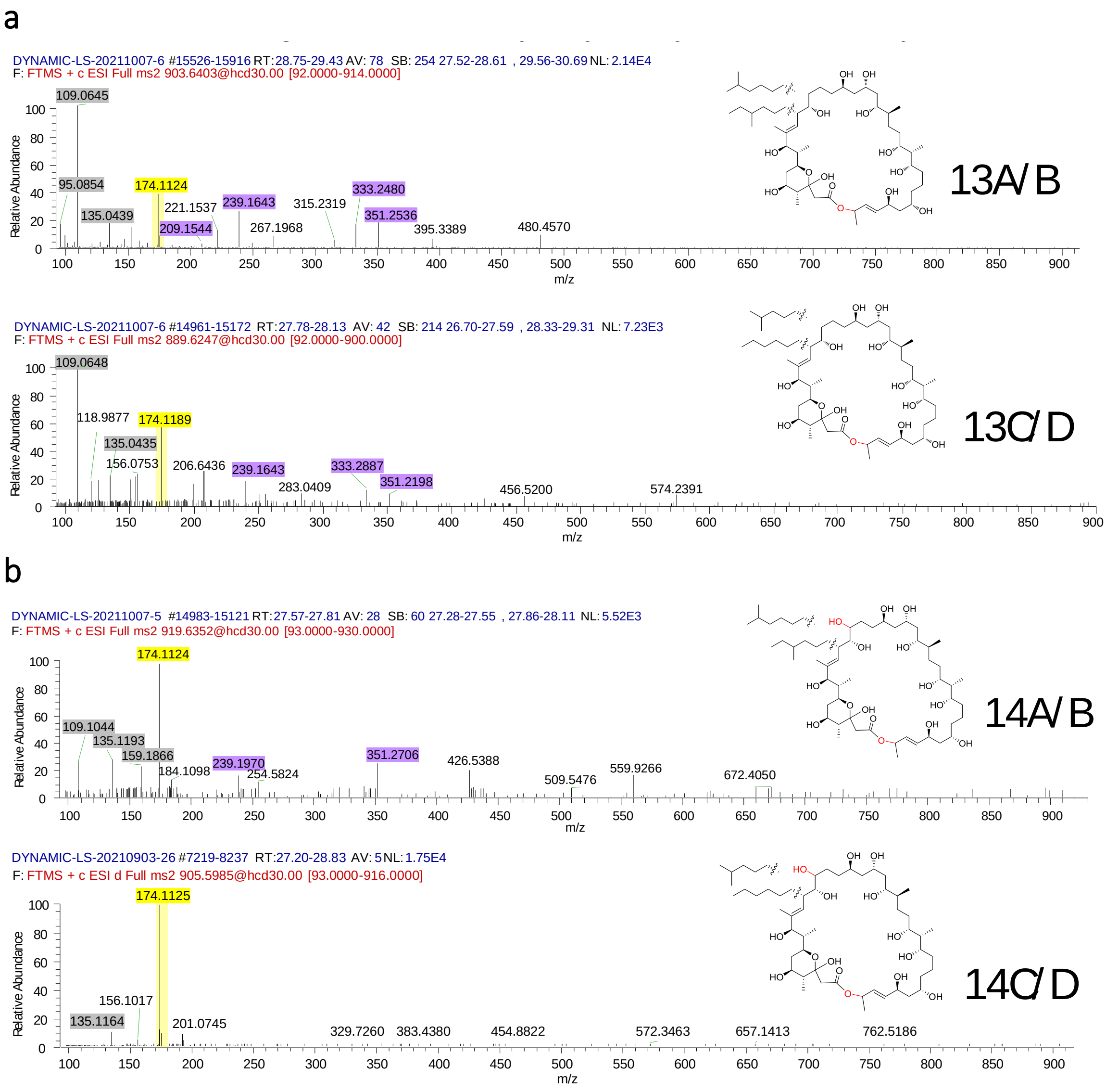

Supplementary Fig. 34. MS/MS analysis of the mini-stambomycins. a MS^2 spectra of non-hydroxylated cyclic mini-stambomycins **13**. **b** MS^2 spectra of hydroxylated cyclic mini-stambomycins **14.** Fragments common to **13** and **14** are indicated in purple, and that characteristic of macrocyclic products in yellow. For full interpretation of these data, and in particular how they support the proposed structures of **13** and **14**, see the **Supplementary Note 1**.

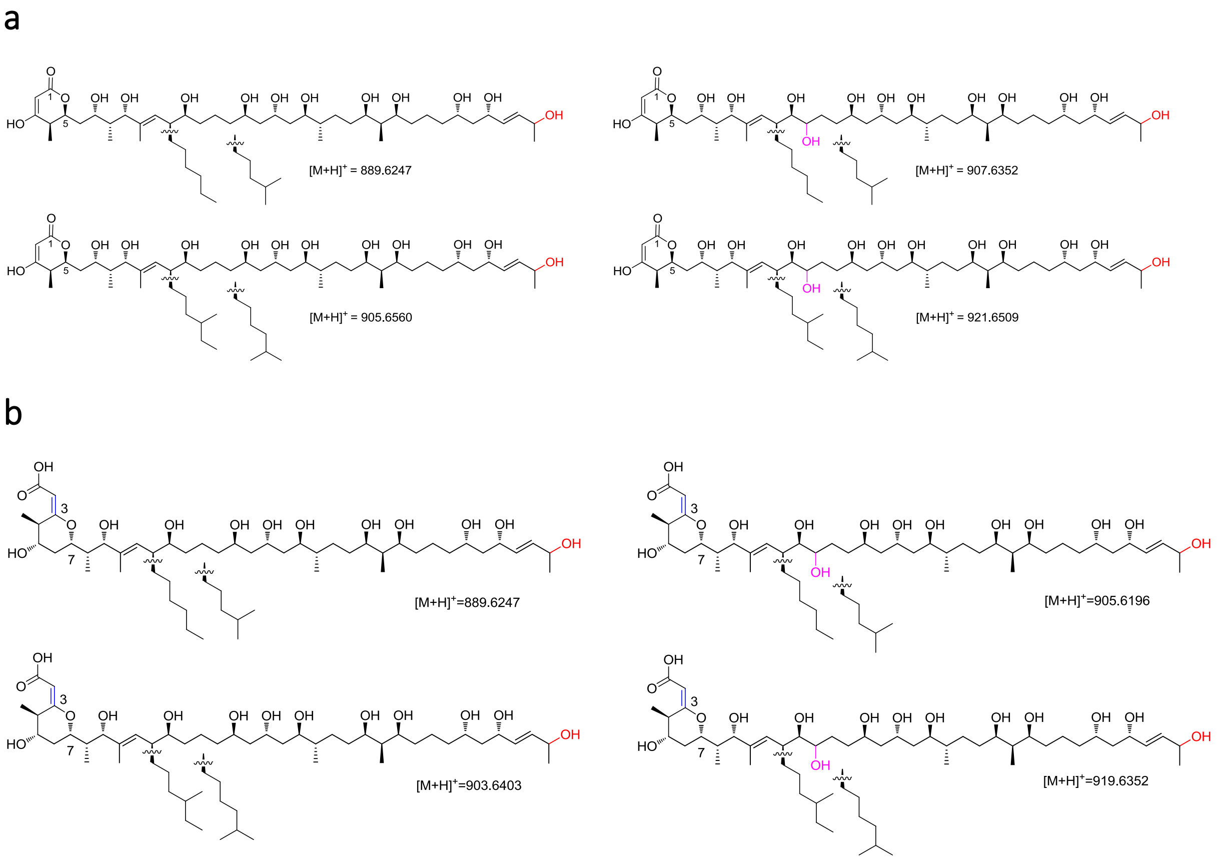

Supplementary Fig. 35. Theoretical structures produced by alternative modes of release of the mini-stambomycin chains from the PKS. a Products arising from dihydropyrone formation (attack on the thioester by the C5-OH). **b** Products arising from hydrolysis and tetrahydropyran formation and dehydration to form a double bond (shown in blue) (as described in [17]).

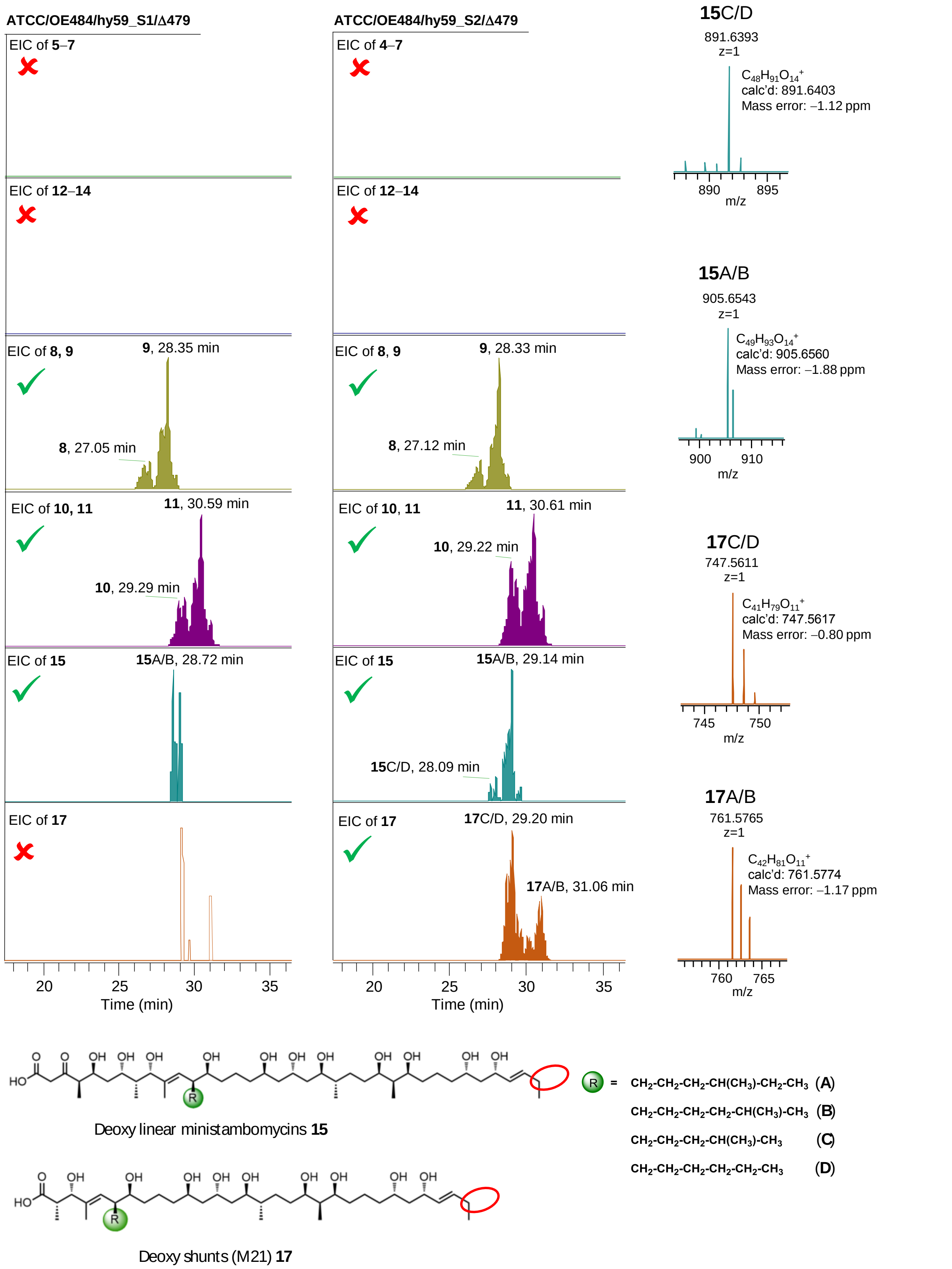

Supplementary Fig. 36. HPLC-MS analysis of inactivation of the P450 hydroxylase SamR0479 in strain ATCC/OE484/hy59_S1 and ATCC/OE484/hy59_S2. As anticipated, the loss of the terminal hydroxyl group prohibited cyclization of the mini-stambomycins, resulting exclusively in production of linear, deoxy **15**. Compound **17**, the deoxy product released from the module 21 ACP, was also detected in the SamR0479 mutant of ATCC/OE484/hy59_S2.

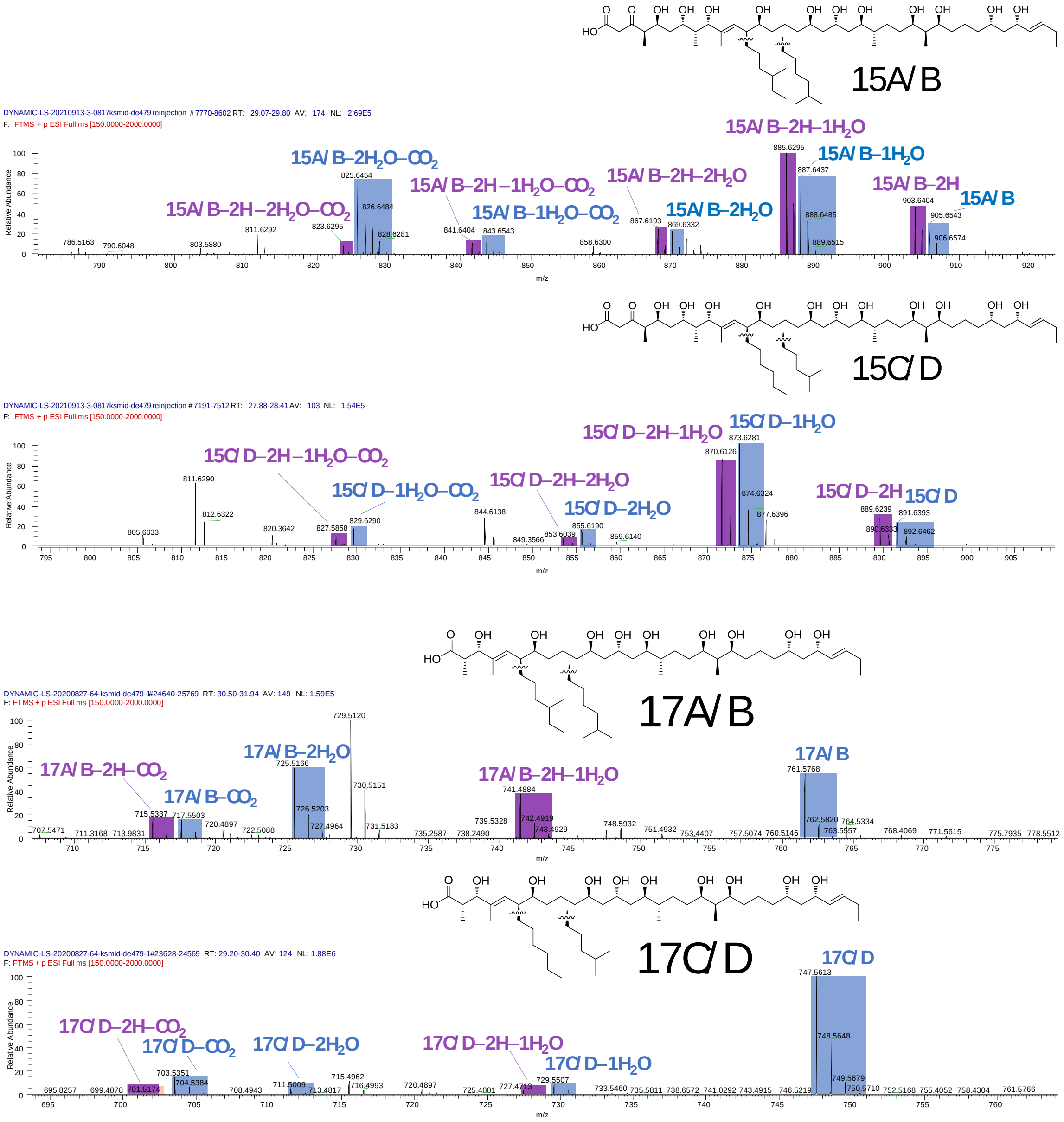

Supplementary Fig. 37. MS analysis of metabolites 15 **and** 17**.** MS spectra of the shunt products **15** and **17**, which are fully consistent with the predicted structures of deoxy versions of the mini-stambomycins and intermediates released from module 21, respectively. Peaks were observed in both cases corresponding to the loss of one or two hydroxyl groups, as well as the terminal CO_2. Notably, for the majority of the fragment peaks (and in the case of **15**, for the parental ion), a second peak was observed at –2 Da (highlighted in purple). We attribute this observation to the absence of the terminal hydroxyl in both **15** and **17**, which may prompt the dysfunction of one of the KRs of Pks9 (modules 21–23), resulting in a ketone group (–2 Da) instead of a hydroxyl at the corresponding position.

a

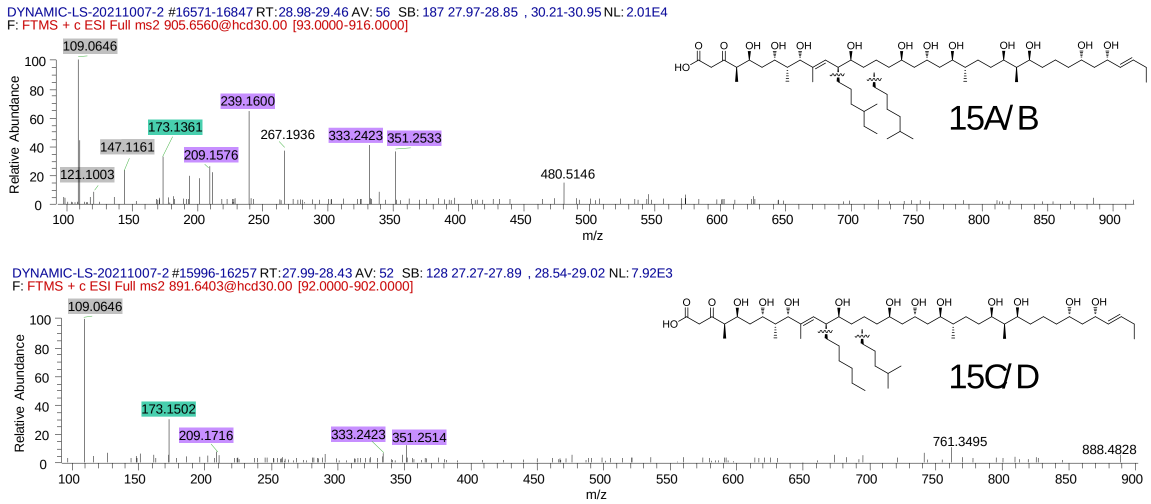

b

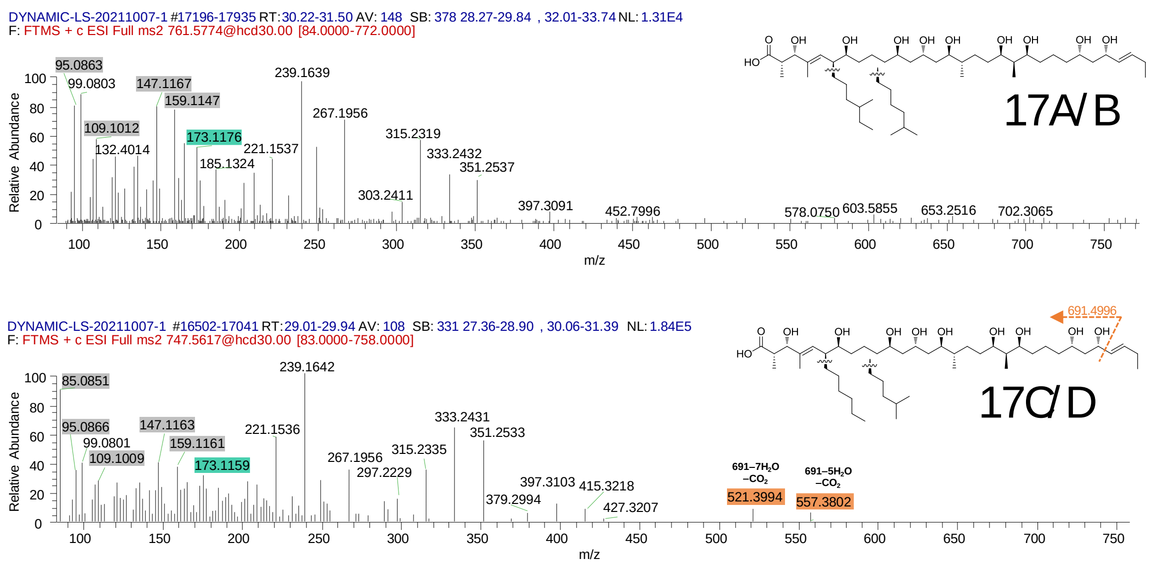

Supplementary Fig. 38. MS/MS analysis of metabolites 15 **and** 17. **a** The MS[2] spectra of **15** notably reveal that the deoxy linear mini-stambomycins **15** exhibit a fragment of 173.1 (colored in mint), which is also observed in the MS[2] pattern of all the linear metabolites (**4–11**, **16** and the 50-deoxystambomycins **2**), but is absent from all cyclic forms (i.e. mini-stambomycins **13** (see **Supplementary Fig. 34**) and parental stambomycins **1** (see **Supplementary Fig. 8**)). Furthermore, as mentioned previously, fragments 351.2, 333.2, 239.2, and 209.2 (highlighted in purple) and 109.1 (highlighted in grey) are common to **15** and the cyclic mini-stambomycins **13** (see **Supplementary Fig. 34**), consistent with their otherwise closely-related structures. **b** In terms of **17**, the MS[2] analysis notably shows the presence of multiple fragments common to all linear metabolites **4–11**, **16** and the 50-deoxystambomycins **2** (173.13 highlighted in mint, and 159.11, 147.11, 121.10, 95.1 and 85.1 highlighted in grey). Analysis of **17**C/D additionally exhibits two 691–XH_2O–CO_2 fragments (shown in orange), which provide direct evidence for the CO_2 terminus.

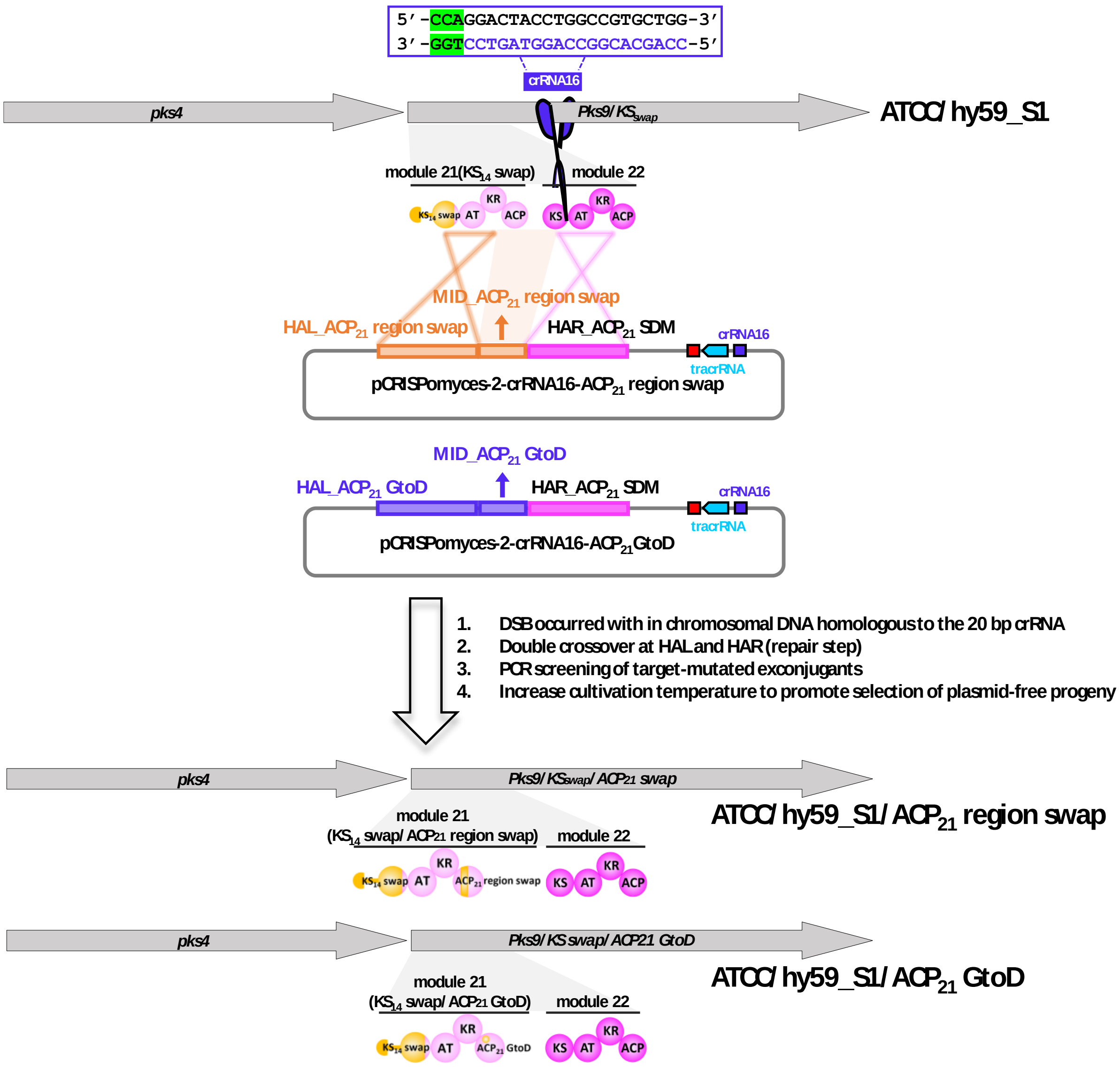
5′-CCAGGACTACCTGGCCGTGCTGG-3′
3′-GGTCCTGATGGACCGGCACGACC-5′
crRNA16
pks4
Pks9/KSswap
ATCC/hy59_S1
module 21(KS14 swap)
module 22
KS14 swap AT KR ACP
KS AT KR ACP
MID_ACP21 region swap
HAL_ACP21 region swap
HAR_ACP21 SDM
crRNA16
tracrRNA
pCRISPomyces-2-crRNA16-ACP21 region swap
MID_ACP21 GtoD
HAL_ACP21 GtoD
HAR_ACP21 SDM
crRNA16
tracrRNA
pCRISPomyces-2-crRNA16-ACP21 GtoD
1. DSB occurred with in chromosomal DNA homologous to the 20 bp crRNA
2. Double crossover at HAL and HAR (repair step)
3. PCR screening of target-mutated exconjugants
4. Increase cultivation temperature to promote selection of plasmid-free progeny
pks4
Pks9/KSswap/ACP21 swap
ATCC/hy59_S1/ACP21 region swap
module 21
(KS14 swap/ACP21 region swap)
module 22
KS14 swap AT KR ACP21 region swap
KS AT KR ACP
pks4
Pks9/KS swap/ACP21 GtoD
ATCC/hy59_S1/ACP21 GtoD
module 21
(KS14 swap/ACP21 GtoD)
module 22
KS14 swap AT KR ACP21 GtoD
KS AT KR ACP

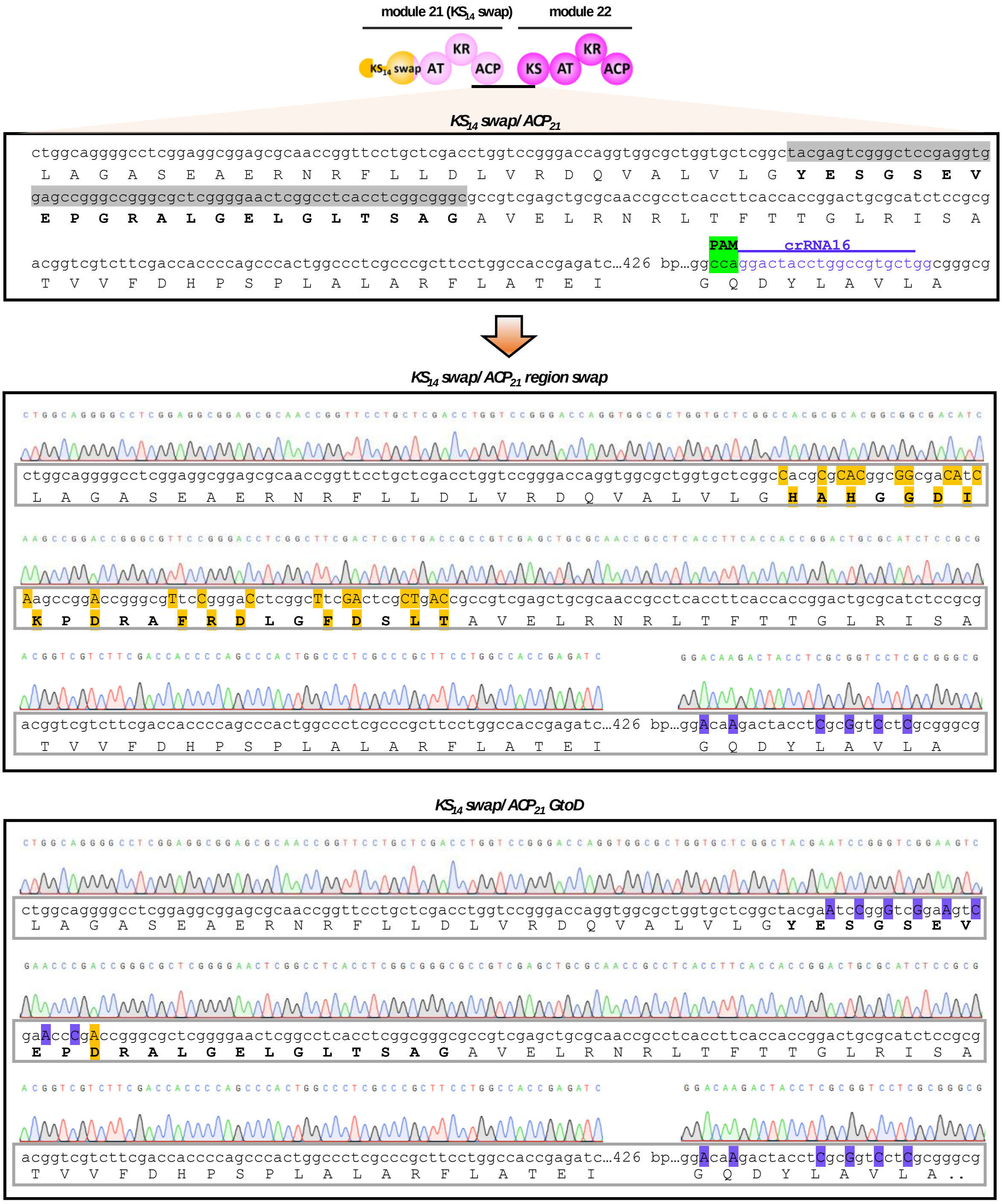

Supplementary Fig. 39. Illustration of the strategy to modify ACP_{21} in strain ATCC/hy59_S1. Residues 31–54 within loop 1 and a portion of helix $\alpha 2$ (L1+H2 region, highlighted in grey) (**Supplementary Fig. 27**) constituting the putative elongation interface on ACP_{21}[13] with its KS partner, were targeted to match those of ACP_{14} (either by exchange of the whole region (highlighted in orange) or by mutation of a single residue within a key two-amino acid recognition motif (GR → DR, as R is common for ACP_{14} and ACP_{21}); the D is highlighted in orange, while additional nucleotides in blue were also modified in order to facilitate screening for correct mutants). Successful generation of the ACP_{21} mutants was verified by DNA sequencing, as was mutation of DNA sequence corresponding to the selected crRNA16 (highlighted in blue), allowing avoidance of Cas9-catalyzed cleavage within the genome of the two resulting mutant strains. Abbreviations: KS, ketosynthase; AT, acyl transferase; ACP, acyl carrier protein; KR, ketoreductase.

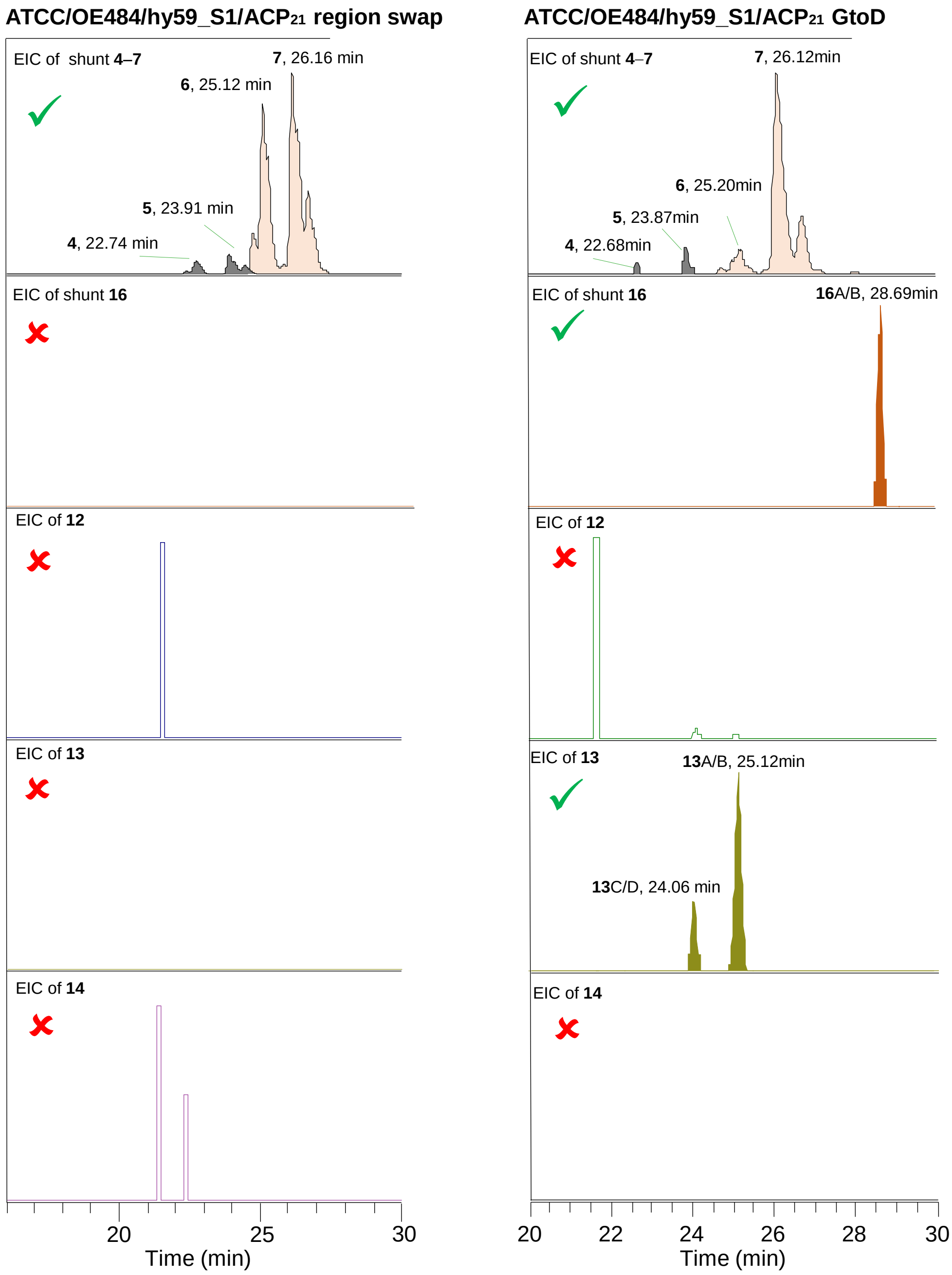

Supplementary Fig. 40. HPLC-MS analysis of mutants ATCC/OE484/hy59_S1/ACP$_{21}$ region swap and ATCC/OE484/hy59_S1/ACP$_{21}$ GtoD. Only the mini-stambomycins **13** were detected in extracts of ATCC/OE484/hy59_S1/ACP$_{21}$ GtoD, along with shunt metabolite **16**, corresponding to chain extension intermediate released from module 21.

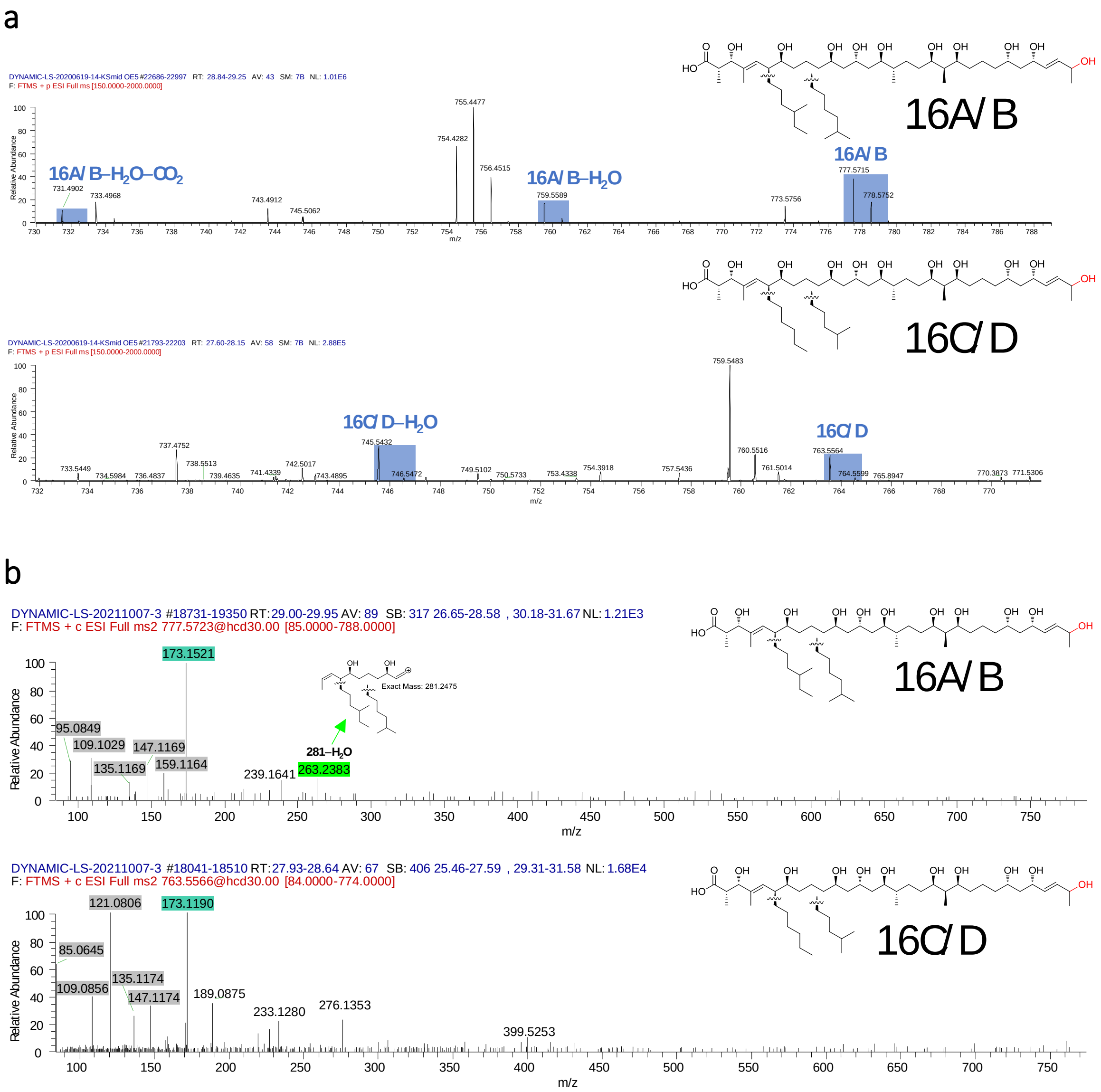

Supplementary Fig. 41. MS/MS analysis of the shunt products 16 **released from module 21. a** MS spectra of **16**. **b** MS^2 spectra of **16**. These data are fully consistent with the predicted structures of intermediates released from module 21. Notably, the MS^2 analysis reveals the presence of multiple fragments common to metabolites **4**–**11** (173.13 highlighted in mint, and 159.1, 147.1, 135.1, 121.1, 109.1, 95.08 and 85.06 highlighted in grey).

Supplementary Table 1. List of amino acid sequences of all docking domain used in this work.

Docking domain (DD)	Amino Acid sequence
$^{C}DD_4$	GPGSYADRLLAELARLEADLTRMDGDEEDRTRLAARLRALADRCQGERTRRDDNL DAASIEEVFDLLDTEFETP
$^{C}DD_4$ SDM	GPGSYADRLLAELARLEADLTRMDGDEEDRTRLAARLRALADRCQGERTRRDDNL DAASIDEVFDLIDTELETP
$^{C}DD_4$ helix swap	GPGSYADRLLAELARLEADLTRMDGDEEDRTRLAARLRALADRCQGERTRRDDNL DAADDDNLFAFIDNELGA
$^{N}DD_5$	GPGSMSDTDPDQQEKLLAYLKRATTQLRDANRRVRELEDRS
$^{C}DD_8$	GPGSYGPAALLADLDRLDAALAGTGPGDETRSRVISRLHALLARHDTADGSGLVTA AGNLDAADDDNLFAFIDNELGA
$^{N}DD_9$ Met	MPDETVGARAVSTEDKLRDYLKRVTTDLASVRQRLRAAEST
$^{N}DD_9$ Val	VSTEDKLRDYLKRVTTDLASVRQRLRAAEST

Note: Residues highlighted in blue indicate mutated and swapped amino acids, red residues were introduced via the cloning vector pBG102, while the tyrosines indicated in green were added to facilitate monitoring during purification and measurement of protein concentration.

Supplementary Table 2. Calculated and observed masses of all metabolites in this study.

Compound	Chemical formula	Retention time (min)	Calculated mass $[M+2H]^{2+}$	Observed mass $[M+2H]^{2+}$	Calculated mass $[M+H]^{+}$	Observed mass $[M+H]^{+}$	Calculated mass $[M+Na]^{+}$	Observed mass $[M+Na]^{+}$
Stambomycins **1**A/B[a]	$C_{73}H_{133}NO_{22}$	22.44	688.9736	688.9697 (−5.66 ppm)[f]	1376.9392	1376.9358 (−2.47 ppm)	1398.9217	n.d.
Stambomycins **1**C/D[a]	$C_{72}H_{131}NO_{22}$	21.44	681.9659	681.9620 (−5.72 ppm)	1362.9236	1362.9207 (−2.13 ppm)	1384.9061	n.d.
Stambomycin **1**E[a]	$C_{71}H_{129}NO_{22}$	21.02	674.9539	674.9531 (−1.19 ppm)	1348.9079	1348.9058 (−1.56 ppm)	1370.8904	n.d.
Stambomycin **1**F[a]	$C_{74}H_{135}NO_{22}$	23.91	694.9815	n.d.	1390.9549	1390.9515 (−2.44 ppm)	1412.9373	n.d.
50-Deoxystambomcyins **2**A/B	$C_{65}H_{120}O_{19}$	26.58	603.4329	n.d.	1205.8497	1205.8490 (−0.58 ppm)	1227.8322	1227.8279 (−3.50 ppm)
50-Deoxystambomcyins **2**C/D	$C_{64}H_{118}O_{19}$	25.69	596.4206	n.d.	1191.8340	1191.8339 (−0.08 ppm)	1213.8165	1213.8124 (−3.38 ppm)
50-Deoxystambomcyins **2**F	$C_{66}H_{122}O_{19}$	28.14	610.4363	n.d.	1219.865	1219.8652 (−0.08 ppm)	1241.8420	1241.8420 (0 ppm)
Erythromycin A **3**	$C_{37}H_{67}NO_{13}$	21.26	367.7379	n.d.	734.4685	734.4685 (0 ppm)	756.4510	n.d.
Shunt (M12) **4**[b]	$C_{35}H_{68}O_{11}$	21.07	333.2452	n.d.	665.4834	665.4831 (−0.45 ppm)	687.4650	687.4649 (−0.15 ppm)
Shunt (M12) 5[b]	$C_{36}H_{70}O_{11}$	22.25	340.2533	n.d.	679.4991	679.4986 (−0.74 ppm)	701.4812	701.4805 (−1.0 ppm)
Shunt (M13) **6**[b]	$C_{38}H_{72}O_{11}$	23.38	353.2607	n.d.	705.5147	705.5144 (−0.43 ppm)	727.4972	727.4961 (−1.51 ppm)
Shunt (M13) **7**[b]	$C_{39}H_{74}O_{11}$	24.37	360.1495	n.d.	719.5304	719.5299 (−0.69 ppm)	741.5129	741.5115 (−1.89 ppm)
Deoxy shunt (M12) **8**[c]	$C_{35}H_{68}O_{10}$	24.88	325.2485	n.d.	649.4889	649.4858 (−4.8 ppm)	671.4710	671.4683 (−4.02 ppm)
Deoxy shunt (M12) **9**[c]	$C_{36}H_{70}O_{10}$	26.11	332.2565	n.d.	663.5051	663.5018 (−5.0 ppm)	685.4867	685.4838 (−4.23 ppm)
Deoxy shunt (M13) **10**[c]	$C_{38}H_{72}O_{10}$	27.18	345.2639	n.d.	689.5198	689.5177 (−3.0 ppm)	711.5023	711.4996 (−3.79 ppm)
Deoxy shunt (M13) **11**[c]	$C_{39}H_{74}O_{10}$	28.27	352.2717	n.d.	703.5355	703.5334 (−3.0 ppm)	725.5180	725.5155 (−3.45 ppm)
Linear ministambomycin **12**A/B[d]	$C_{49}H_{92}O_{15}$	25.38	461.3294	n.d.	921.6509	921.6499 (−1.09 ppm)	943.6334	n.d.
Linear ministambomycin **12**C/D[d]	$C_{48}H_{90}O_{15}$	24.45	454.3216	n.d.	907.6352	907.6346 (−0.66 ppm)	929.6177	n.d.
Non-hydroxylated cyclic ministambomycin **13**A/B[d]	$C_{49}H_{90}O_{14}$	25.37	452.3241	n.d.	903.6403	903.6388 (−1.66 ppm)	925.4428	n.d.
Non-hydroxylated cyclic ministambomycin **13**C/D[d]	$C_{48}H_{88}O_{14}$	24.44	445.3159	n.d.	889.6247	889.6237 (−1.12 ppm)	911.6072	n.d.
Hydroxylated cyclic ministambomycin **14**A/B[d]	$C_{49}H_{90}O_{15}$	25.17	460.3216	n.d.	919.6352	919.6348 (−0.43 ppm)	941.6177	n.d.
Hydroxylated cyclic ministambomycin **14**C/D[d]	$C_{48}H_{88}O_{15}$	24.15	453.3138	n.d.	905.6196	905.6189 (−0.77 ppm)	927.6021	n.d.
Deoxy linear ministambomycin **15**A/B[e]	$C_{49}H_{92}O_{14}$	29.14	453.3320	n.d.	905.6560	905.6543 (−1.88 ppm)	927.6358	n.d.
Deoxy linear ministambomycin **15**C/D[e]	$C_{48}H_{90}O_{14}$	28.09	446.3241	n.d.	891.6403	891.6393 (−1.12 ppm)	913.6228	n.d.
Shunt (M21) **16**A/B[d]	$C_{42}H_{80}O_{12}$	27.01	389.2897	n.d.	777.5723	777.5714 (−1.16 ppm)	799.5547	n.d.
Shunt (M21) **16**C/D[d]	$C_{41}H_{78}O_{12}$	27.98	382.2819	n.d.	763.5566	763.5564 (−0.26 ppm)	785.5391	n.d.
Deoxy shunt (M21) **17**A/B[e]	$C_{42}H_{80}O_{11}$	31.06	381.2923	n.d.	761.5774	761.5765 (−1.17 ppm)	783.5598	n.d.
Deoxy shunt (M21) **17**C/D[e]	$C_{41}H_{78}O_{11}$	29.20	374.2845	n.d.	747.5617	747.5611 (−0.80 ppm)	769.5442	n.d.

n.d. : not detected.

[a]: Retention time and observed masses representive of ATCC/OE484, see **Fig. 3a**.

[b]: Retention time and observed masses representive of CPN2/OE484, see **Figs. 3b** and **c**. Delayed retention times for these compounds was observed during analysis of CPN2/OE484/ACP_{13} SDM (Supplementary Fig. 29) due to a change in the HPLC-MS.

[c]: Retention time and observed masses representive of CPN2/OE484/Δ479, see **Fig. 3c**.

[d]: Retention time and observed masses representive of ATCC/OE484/hy59_S2, see **Supplementary Fig. 32**.

[e]: Retention time and observed masses representive of ATCC/OE484/hy59_S2/Δ479, see **Supplementary Fig. 32**.

[f]: The values in parentheses indicate the mass errors between the theoretical (calculated) *m/z* and the experimentally observed *m/z*.

Supplementary Table 3. Demonstration of the strong agreement between using the correction factor of 206 (highlighted in yellow) and the 50-deoxystambomycins 2A/B standard curve (blue) to estimate metabolite yields.

ATCC/OE484/hy59_S1 (KS_{14} swap) and ATCC/OE484/hy59_S2 ($KS_{14/21}$)														
Strains (culture volume)	Integrated peak areas (and calculated yield mg L^{-1}) of shunt products						Linear ministambomycin **12**		Non-hydroxylated cyclic ministambomycin **13**		Hydroxylated cyclic ministambomycin **14**		Mass of cells (g)	Volume of final extract in MeOH (µL)
	**4** (665.4825 [M+H]) (M12)	**5** (679.4987 [M+H]) (M12)	**6** (705.5134 [M+H], 727.4972 [M+Na]) (M13)	**7** (719.5291 [M+H], 741.5129 [M+Na]) (M13)	**16**C/D (763.5566 [M+H]) (M21)	**16**A/B (777.5713 [M+H]) (M21)	**12**C/D (907.6352 [M+H])	**12**A/B (921.6509 [M+H])	**13**C/D (889.6247 [M+H])	**13**A/B (903.6403 [M+H])	**14**C/D (905.6196 [M+H])	**14**A/B (919.6352 [M+H])		
20200619-KS_{14} swap /OE-1 (50ml)	6293693<br>(0.0052–1.1)<br>(0.0052–1.1)	5052020<br>(0.0043–0.89)<br>(0.0043–0.86)	34501397<br>(0.026–5.3)<br>(0.026–5.6)	25414414<br>(0.019–3.9)<br>(0.019–4.1)	n.d.	n.d.	n.d.	n.d.	n.d.	n.d.	n.d.	n.d.	5	370
20200619- KS_{14} swap/OE-2 (50ml) ✓	42879664<br>(0.029–5.9)<br>(0.029–6.3)	96663771<br>(0.066–14)<br>(0.066–14)	343133671<br>(0.23–48)<br>(0.23–50)	584008582<br>(0.39–81)<br>(0.39–85)	n.d.	n.d.	84543<br>(0.00053–0.11)<br>(0.00053–0.054)	420957<br>(0.00076–0.16)<br>(0.00076–0.10)	1206749<br>(0.0013–0.27)<br>(0.0013–0.22)	2074971<br>(0.0019–0.39)<br>(0.0019–0.34)	n.d.	725054<br>(0.00097–0.20)<br>(0.00097–0.15)	4.5	335
20200619-KS_{14} swap/OE-3 (50ml)	7089726<br>(0.06–1.3)<br>(0.06–1.3)	18341831<br>(0.015–3.1)<br>(0.015–3.2)	53007611<br>(0.042–8.7)<br>(0.042–9.1)	75394172<br>(0.060–12)<br>(0.060–13)	n.d.	n.d.	n.d.	n.d.	n.d.	n.d.	n.d.	n.d.	5.2	390
20200619-KS_{14} swap/OE-4 (50ml*3)	30757896<br>(0.015–3.1)<br>(0.015–3.2)	400063135<br>(0.19–38)<br>(0.19–41)	215411738<br>(0.10–21)<br>(0.10–22)	220952290<br>(0.10–21)<br>(0.10–23)	n.d.	n.d.	n.d.	n.d.	n.d.	n.d.	n.d.	n.d.	9.5	700
20200619-KS_{14} swap/OE-5 (50ml*3)	6247919<br>(0.0036–0.74)<br>(0.0036–0.73)	5054689<br>(0.003–0.62)<br>(0.003–0.59)	33375699<br>(0.018–3.7)<br>(0.018–3.7)	25597428<br>(0.014–2.9)<br>(0.014–2.9)	n.d.	n.d.	n.d.	n.d.	n.d.	n.d.	n.d.	n.d.	10.3	770

Supplementary Table 4. Quantification of stambomycins in wild type and DD engineering strains.

Strains (culture volume)	Integrated peak areas for stambomycins **1** A, B, C, D (EI = 1362.9236, 681.9659, 1376.9392, 688.9736)	Mass of cells (g)	Volume of final extract in MeOH (μL)	Calculated titer (mg L^{-1})	Average ± deviation (wild type level, %)
20190130-9-WTOE (50ml)	4672442552	3.8	300	28.2	30 ± 2 (100%)
20190913-13-WTOE (50ml)	6621933694	3.2	240	32	
20190206-4-k7n6 (50ml)	4911596030	2.9	200	19.8	22 ± 3 (73%)
20190206-6-k7n6 (50ml)	5596413718	3	210	23.7	
20180719-2-k7n4 (50ml)	1354939122	3.2	225	6.1	5 ± 1 (18%)
20190206-4-k7n4 (50ml)	1308555295	2.6	180	4.7	
20181211-8-cpn4 (50ml)	2173114763	2.7	190	8.3	7 ± 2 (23%)
20181211-9-cpn4 (50ml)	1730753566	2.3	160	5.6	
20181211-11-cpn5 (50ml)	848360698	2.8	195	3.3	4 ± 1 (14%)
20181211-12-cpn5 (50ml)	1389831827	2.7	190	5.3	

Supplementary Table 5. Quantification of shunt products in DD engineering strains.

Strains (culture volume)	Integrated areas of peaks corresponding to shunt products				Mass of cells (g)	Volume of final extract in MeOH (μL)	wild type level, %
	**4** (665.4825 [M+H])	**5** (679.4987 [M+H])	**6** (705.5134 [M+H], 727.4972 [M+Na])	**7** (719.5291 [M+H], 741.5129 [M+Na])			
20190226-9-k7n1/OE (50ml)	48227	116879	239428	1992226	3.2	220	
20190405-22-k7n1/OE (50ml)	30888	76662	140487	924533	2.2	170	
20190206-1-k7n1/OE (50ml)	198822	302791	234494	1493742	3	210	
**Average**	92646	165444	204803	1470167			
**(Calculated titers (mg L^{-1})**	(0.00066–0.14)	(0.00095–0.20)	(0.0011–0.23)	(0.0062–1.3)		200	0.030%–6.2%
20190226-5-k7n2/OE (50ml)	68169	206607	1365494	1831999	2.4	170	
20190405-23-k7n2/OE (50ml)	5585	13638	526592	1296303	2.8	200	
**Average**	36877	110123	946043	1564151			
**(Calculated titers (mg L^{-1})**	(0.00040–0.082)	(0.00067–0.14)	(0.0038–0.72)	(0.0061–1.3)		185	0.037%–7.6%
20190206-3-k7n3/OE (50ml)	42023	186837	1124713	2296192	2	140	
20190405-24-k7n3/OE (50ml)	n.d.	n.d.	235449	265668	3	210	
**Average**	42023	186837	680081	1280930			
**(Calculated titers (mg L^{-1})**	(0.00039–0.08)	(0.0009–0.19)	(0.0026–0.54)	(0.0048–0.99)		175	0.029–6.0%
20180719-3-k7n5/OE (50ml)	67382	81574	83076	1221210	2.5	180	
20190206-5-k7n5/OE (50ml)	34500	93184	692770	1962094	1.6	120	
20190405-25-k7n5/OE (50ml)	23563	45805	801063	1377565	3	210	
**Average**	41815	73521	525636	1520290			
**(Calculated titers (mg L^{-1})**	(0.00038–0.078)	(0.00049–0.10)	(0.002–0.41)	(0.0054–1.1)		170	0.028–5.8%
20190206-7-cpn1/OE (50ml)	51402	197611	1139676	1799995	2	140	
20190206-8-cpn1/OE (50ml)	58567	158339	979740	2142216	1.7	130	
20190405-26-cpn1/OE (50ml)	7333	21868	871163	1337523	3	210	
**Average**	39101	125939	996860	1759911			
**(Calculated titers (mg L^{-1})**	(0.00035–0.072)	(0.00063–0.13)	(0.0034–0.7)	(0.0059–1.2)		160	0.034–7.0%
20180719-8-cpn2/OE (50ml)	23569	322174	810205	1038139	2.8	200	
20190206-9-cpn2/OE (50ml) ✓	73450	169845	1062465	2364460	2.4	150	
20190206-10-cpn2/OE (50ml)	55026	132822	933593	2287611	2.5	150	
20190405-27-cpn2/OE (50ml)	3405	10586	780584	1087050	3	210	
**Average**	38863	158857	896712	1694315			
**(Calculated titers (mg L^{-1})**	(0.00039–0.08)	(0.00082–0.17)	(0.0035–0.72)	(0.0063–1.3)		178	0.037–7.6%
20190130-5-Pks4+TEI/OE (50ml)	622957	1920826	22589795	43419150	3.8	150	
20190130-6-Pks4+TEI/OE (50ml)	740404	1864042	20950108	41939099	4	150	
20190130-17-Pks4+TEI/OE (50ml)	345519	944340	16606807	30752675	2.7	200	
20190130-18-Pks4+TEI/OE (50ml)	216270	587675	11314880	20453416	2.6	200	
**Average**	481288	766008	17865398	34141085			
**(Calculated titers (mg L^{-1})**	(0.0019–0.39)	(0.0029–0.60)	(0.063–13)	(0.121–25)		175	0.63–130%
20190405-37-cpn2/OE/TEI SDM (50ml*2)	2659	3581	338990	451109			
**(Calculated titers (mg L^{-1})**	(0.00026–0.054)	(0.00026–0.054)	(0.0014–0.29)	(0.0018–0.37)	4.8	350	0.012–2.5%
20190529-7-cpn2/OE/TEII SDM (50ml)	3928	27230	479426	1070306	2.65	190	
20190529-8-cpn2/OE/TEII SDM (50ml)	4232	34540	620282	1353119	2.7	200	
**Average**	4080	30885	549854	1211713			
**(Calculated titers (mg L^{-1})**	(0.00029–0.060)	(0.00040–0.082)	(0.0024–0.49)	(0.005–1.0)		195	0.027–5.6%
20190405-6-cpn2/OE/Δ478 (50ml)	19451	67231	823021	1458241	3	210	
20190405-7-cpn2/OE/Δ478 (50ml)	19789	47188	792059	1385322	2.6	190	
**Average**	19620	57210	807540	1421782			
**(Calculated titers (mg L^{-1})**	(0.00036–0.074)	(0.00051–0.11)	(0.0035–0.72)	(0.0060–1.2)		200	0.035–7.2%
DD engineering strainCPN2/OE484 in which SamR0479 is inactivated							
Strains (culture volume)	Integrated areas of deoxy shunt products				Mass of cells (g)	Volume of final extract in MeOH (μL)	wild type level, %
	**8** (649.4889 [M+H])	**9** (663.5051 [M+H])	**10** (689.5198 [M+H], 711.5023 [M+Na])	**11** (703.5355 [M+H], 725.5180 [M+Na])			
20190130-23-cpn2/OE/Δ479 (50ml)	14429	43625	667345	1236812	2.8	200	
20190130-24-cpn2/OE/Δ479 (50ml)	15398	40334	335929	810120	3	210	
**Average**	14914	41980	501637	1023466			
**(Calculated titers (mg L^{-1})**	(0.00035–0.072)	(0.00046–0.095)	(0.0024–0.49)	(0.0045–0.93)		205	0.026–5.4%

Supplementary Table 6. Quantification of shunts and cyclic mini-stambomycins in interface engineering strains.

Strains<br>(culture volume)	Integrated peak areas (and calculated yield mg L^{-1}) of shunt products						Linear ministambomycin **12**		Non-hydroxylated cyclic ministambomycin **13**		Hydroxylated cyclic ministambomycin **14**		Mass of cells (g)	Volume of final extract in MeOH (μL)
	**4**<br>(665.4825 [M+H])<br>(M12)	**5**<br>(679.4987 [M+H])<br>(M12)	**6**<br>(705.5134 [M+H], 727.4972 [M+Na])<br>(M13)	**7**<br>(719.5291 [M+H], 741.5129 [M+Na])<br>(M13)	**16**C/D<br>(763.5566 [M+H])<br>(M21)	**16**A/B (777.5713 [M+H])<br>(M21)	**12**C/D<br>(907.6352 [M+H])	**12**A/B<br>(921.6509 [M+H])	**13**C/D<br>(889.6247 [M+H])	**13**A/B<br>(903.6403 [M+H])	**14**C/D<br>(905.6196 [M+H])	**14**A/B<br>(919.6352 [M+H])		
20200619-KS_{14} swap/OE-1 (50ml)	6293693<br>(0.0052–1.1)	5052020<br>(0.0043–0.89)	34501397<br>(0.026–5.3)	25414414<br>(0.019–3.9)	n.d.	n.d.	n.d.	n.d.	n.d.	n.d.	n.d.	n.d.	5	370
20200619- KS_{14} swap/OE-2 (50ml) ✓	42879664<br>(0.029–5.9)	96663771<br>(0.066–14)	343133671<br>(0.23–48)	584008582<br>(0.39–81)	n.d.	n.d.	84543<br>(0.00053–0.11)	420957<br>(0.00076–0.16)	1206749<br>(0.0013–0.27)	2074971<br>(0.0019–0.39)	n.d.	725054<br>(0.00097–0.20)	4.5	335
20200619-KS_{14} swap/OE-3 (50ml)	7089726<br>(0.06–1.3)	18341831<br>(0.015–3.1)	53007611<br>(0.042–8.7)	75394172<br>(0.060–12)	n.d.	n.d.	n.d.	n.d.	n.d.	n.d.	n.d.	n.d.	5.2	390
20200619-KS_{14} swap/OE-4 (50ml*3)	30757896<br>(0.015–3.1)	400063135<br>(0.19–38)	215411738<br>(0.10–21)	220952290<br>(0.10–21)	n.d.	n.d.	n.d.	n.d.	n.d.	n.d.	n.d.	n.d.	9.5	700
20200619-KS_{14} swap/OE-5 (50ml*3)	6247919<br>(0.0036–0.74)	5054689<br>(0.003–0.62)	33375699<br>(0.018–3.7)	25597428<br>(0.014–2.9)	n.d.	n.d.	n.d.	n.d.	n.d.	n.d.	n.d.	n.d.	10.3	770
20200619-$KS_{14/21}$/OE-1 (50ml)	47039781<br>(0.029–5.9)	85889429<br>(0.052–11)	315910063<br>(0.19–39)	406463091<br>(0.25–51)	n.d.	n.d.	n.d.	n.d.	n.d.	n.d.	n.d.	n.d.	4.1	300
20200619-$KS_{14/21}$/OE-2 (50ml)	18613021<br>(0.015–3.1)	40766899<br>(0.031–6.4)	140489502<br>(0.11–22)	198526766<br>(0.15–31)	n.d.	n.d.	n.d.	n.d.	n.d.	n.d.	n.d.	n.d.	5	375
20200619-$KS_{14/21}$/OE-3 (50ml) ✓	107314758<br>(0.065–13)	242618264<br>(0.15–30)	770881040<br>(0.47–96)	1120574541<br>(0.067–14)	n.d.	766578<br>(0.0089–0.18)	2018597<br>(0.0016–0.33)	2844624<br>(0.0021–0.43)	5031092<br>(0.0035–0.72)	9617988<br>(0.0062–1.3)	262071<br>(0.00058–0.12)	4187448<br>(0.0030–0.62)	4.1	300
20200619-$KS_{14/21}$/OE-4 (50ml*3)	91405522<br>(0.045–9.3)	123907364<br>(0.0061–13)	600891652<br>(0.29–60)	710632670<br>(0.34–71)	n.d.	n.d.	n.d.	n.d.	n.d.	n.d.	n.d.	n.d.	9.6	720
20200619-$KS_{14/21}$/OE-5 (50ml*2)	87894343<br>(0.044–9.1)	139795773<br>(0.069–14)	682188694<br>(0.34–69)	829685866<br>(0.41–84)	3641918<br>(0.0021–0.43)	13077739<br>(0.0068–1.4)	1570620<br>(0.0011–0.23)	1046799<br>(0.00086–0.18)	4674276<br>(0.0026–0.54)	4526109<br>(0.00026–0.54)	1605608<br>(0.0011–0.23)	2979577<br>(0.0018–0.37)	6.6	490
CPN2/OE484/ACP_{13} SDM														
20201120-CPN2/ACP_{13} SDM-OE1(50mL)	12640195<br>(0.0066–1.4)	32997824<br>(0.017–3.5)	308796712<br>(0.15–31)	809571533<br>(0.40–82)	n.d.	n.d.	n.d.	n.d.	n.d.	n.d.	n.d.	n.d.	3.5	245
20201120-CPN2/ACP_{13} SDM-OE2 (50mL) ✓	6362591<br>(0.0040–0.82)	17482021<br>(0.010–2.1)	235144379<br>(0.13–27)	670480570<br>(0.38–78)	n.d.	n.d.	n.d.	n.d.	n.d.	179421<br>(0.00050–0.10)	n.d.	n.d.	4	280
20201130-CPN2/ACP_{13} SDM-OE3 (50mL*3)	6704419<br>(0.0035–0.72)	18944889<br>(0.0092–1.9)	135610351<br>(0.064–13)	380365058<br>(0.18–37)	n.d.	n.d.	n.d.	n.d.	n.d.	n.d.	n.d.	n.d.	10	700
20201130-CPN2/ACP_{13} SDM-OE4 (50mL*3)	9101131<br>(0.0039–0.80)	26836822<br>(0.011–2.3)	127172374<br>(0.052–11)	358181384<br>(0.14–30)	n.d.	n.d.	n.d.	n.d.	n.d.	218373<br>(0.00037–0.076)	n.d.	n.d.	8.5	600
20201130-CPN2/ACP_{13} SDM-OE5 (50mL*3)	10548306<br>(0.0060–1.2)	25926832<br>(0.0014–2.9)	146224041<br>(0.078–16)	415504574<br>(0.22–46)	n.d.	n.d.	n.d.	n.d.	n.d.	n.d.	n.d.	n.d.	10.5	800
ATCC/hy59_S1 (KS_{14} swap) + ACP_{21} region swap														
20201120- KS_{14} swap/ACP21 swap-OE1 (50mL) ✓	13839888<br>(0.092–1.9)	22759248<br>(0.015–3.1)	430220249<br>(0.27–56)	555230058<br>(0.35–73)	n.d.	n.d.	n.d.	n.d.	n.d.	n.d.	n.d.	n.d.	4.5	315
20201120- KS_{14} swap/ACP21 swap-OE2 (50mL)	10647439<br>(0.0064–1.3)	10943471<br>(0.066–1.4)	292119102<br>(0.17–34)	241546700<br>(0.14–28)	n.d.	n.d.	n.d.	n.d.	n.d.	n.d.	n.d.	n.d.	4	280
20201130- KS_{14} swap/ACP21 swap-OE3 (50mL*3)	11696575<br>(0.0075–1.5)	21720871<br>(0.014–2.9)	264715440<br>(0.16–33)	392408919<br>(0.24–49)	n.d.	n.d.	n.d.	n.d.	n.d.	n.d.	n.d.	n.d.	13	900
20201130- KS_{14} swap /ACP21 swap-OE4 (50mL*3)	6352427<br>(0.0040–0.82)	8198604<br>(0.0051–1.1)	147643760<br>(0.085–17)	199699615<br>(0.11–23)	n.d.	n.d.	n.d.	n.d.	n.d.	n.d.	n.d.	n.d.	12.5	850
ATCC/hy59_S1 (KS_{14} swap) /ACP_{21} GtoD														
20201218-KS_{14} swap/ACP21 GtoD-OE1 (50mL)	5790815<br>(0.0026–0.5)	9526123<br>(0.0041–0.82)	51985855<br>(0.021–4.3)	258802864<br>(0.10–21)	n.d.	n.d.	n.d.	n.d.	205370<br>(0.00037–0.076)	673895<br>(0.00055–0.11)	n.d.	n.d.	2.8	200
20201218-KS_{14} swap/ACP21 GtoD-OE2 (50mL) ✓	1873317<br>(0.0010–0.21)	6897912<br>(0.0031–0.64)	37197787<br>(0.015–3.1)	274426649<br>(0.11–23)	n.d.	285110<br>(0.00040–0.082)	n.d.	n.d.	154018<br>(0.00034–0.070)	958112<br>(0.00067–0.14)	n.d.	n.d.	2.6	200
20201218-KS_{14} swap/ACP21 GtoD-OE3 (50mL)	3979784<br>(0.0019–0.39)	14314951<br>(0.0060–1.2)	67053419<br>(0.027–5.6)	215967586<br>(0.087–18)	n.d.	n.d.	n.d.	n.d.	n.d.	492423<br>(0.00048–0.099)	n.d.	n.d.	2.2	200
20201218-KS_{14} swap/ACP21 GtoD-OE4 (50mL)	5173540<br>(0.0024–0.49)	17037931<br>(0.0071–1.5)	64276652<br>(0.026–5.3)	189687875<br>(0.077–16)	n.d.	n.d.	n.d.	n.d.	161170<br>(0.00035–0.072)	433372<br>(0.00045–0.093)	n.d.	n.d.	3.0	200
20201218-KS_{14} swap/ACP21 GtoD-OE5 (50mL)	2120956<br>(0.0011–0.23)	8289793<br>(0.0036~0.74)	46422798<br>(0.019~3.9)	191692136<br>(0.077 – 16)	n.d.	248873<br>(0.00038–0.078)	n.d.	n.d.	n.d.	771030<br>(0.00059–0.12)	n.d.	n.d.	2.4	200
20201218-KS_{14} swap/ACP21 GtoD-OE6 (50mL)	5742932<br>(0.0026–0.54)	10447012<br>(0.0045–0.93)	82037304<br>(0.033–6.8)	244427835<br>(0.099–20)	n.d.	n.d.	n.d.	n.d.	n.d.	n.d.	n.d.	n.d.	2.4	200

Supplementary Table 7. Quantification of shunts and linear deoxy mini-stambomycins in interface engineering strains.

	Integrated peak areas of deoxy shunt products						Linear deoxy ministambomycin 15		Mass of cells (g)	Volume of final extract in MeOH (μL)
	**8** (649.4889 [M+H]) (M12)	**9** (663.5051 [M+H]) (M12)	**10** (689.5198 [M+H], 711.5023 [M+Na]) (M13)	**11** (703.5355 [M+H], 725.5180 [M+Na]) (M13)	**17**C/D (747.5617 [M+H]) (M21)	**17**A/B (761.5774 [M+H]) (M21)	**15**C/D (891.6403 [M+H])	**15**A/B (905.6560 [M+H])		
20200827-57-KS_{14} swap/OE/Δ479-1 (50ml*2)	33248315 (0.0089–1.8)	90059352 (0.024–4.9)	304965795 (0.080–16)	585412754 (0.15–32)	n.d.	n.d.	n.d.	121298 (0.00021–0.043)	8	260
20200827-58-KS_{14} swap/OE/Δ479-2 (50ml*2) ✓	27792311 (0.0075–1.5)	101651512 (0.029–6.0)	245985991 (0.065–13)	615185973 (0.16–33)	n.d.	n.d.	n.d.	278506 (0.00026–0.053)	7.5	260
20200827-64-$KS_{14/21}$/OE/Δ479-1 (50ml*2)	57473515 (0.015–3.1)	215089188 (0.057–12)	694181285 (0.18–37)	513896986 (0.14–28)	15066416 (0.0041–0.84)	794478 (0.0004–0.082)	n.d.	619726 (0.00035–0.072)	7.5	260
20200827-65-$KS_{14/21}$/OE/Δ479-2 (50ml*2) ✓	133697567 (0.035–7.2)	649046324 (0.17–35)	1295140013 (0.34–70)	2231045625 (0.58–120)	3694680 (0.0012–0.25)	276172 (0.00026–0.054)	n.d.	5188594 (0.0015–0.31)	8	260

For clarity, in certain instances, average calculated yields are indicated in bold. ✓ The marked entries refer to the highest yields of metabolites produced by the corresponding mutants. MS analysis was performed on Thermo Fisher LTQ Orbitrap mass spectrometer prior to 2020, whereas subsequently, the MS analysis and the standardization were carried out on a Thermo Fisher Orbitrap ID-X Tribrid mass spectrometer. As the later mass spectrometer is more sensitive, all of the data acquired with the first instrument were re-scaled by multiplying by 10 (a factor of 10 was determined by analysis of identical samples on the two machines) prior to yield calculation. In addition, as there was substantial biological variability with fermentation of certain strains (with compounds present above or below the detection limits), we chose not to calculate an average yield, but rather to present the individual data.

Supplementary Table 8. List of new metabolites present in various engineered mutants containing the overexpressed regulator, by comparison to the corresponding mutants containing empty plasmid pIB139 and WT/OE484.

K7N1/OE484	No stambomycins<br>532.3440, 444.2592, 458.2838(+2H), 651.3896, 576.4211, 419.2992, 474.3443(+2H), 425.3361, 480.8504(+2H), 386.7922(+2H), 705.5137, 719.5290, 660.4785
K7N2/OE484	No stambomycins<br>1348.429, 1371.437, 705.5142, 719.5297, 1364.929, 1370.433, 1518.038
K7N4/OE484	Stambomycins<br>661.9570 (+2H), 695.4724 (+2H), 697.4833 (+2H), 669.9518 (+2H), 695.4736 (+2H), 699.9836 (+2H), 664.4550 (+2H), 683.9503 (+2H), 658.9611 (+2H), 698.9536 (+2H), 660.4896 (+2H), 672.4711 (+2H), 678.9785 (+2H), 796.5922, 671.4810, 670.9792, 547.4097 (+2H), 556.4149 (+2H)
K7N5/OE484	NO stambomycins<br>546.8807 (+2H), 553.8887 (+2H), 705.5132, 719.5291
K7N6/OE484	Same with WT/pOE484, no new metabolites
CPN1/OE484	No stambomycins<br>705.5130, 719.5289, 858.6297
CPN2/OE484	No stambomycins<br>705.5134, 719.5291, 524.3039 (+2H), 543.4360, 783.5574

All masses are presented in the order of retention time, while the masses highlighted in red are consistent with the predicted shunt products.

Supplementary Note 1

The following outlines in detail all of the evidence we obtained for the proposed structures of cyclic mini-stambomycins A–D **13**, and their C-14 hydroxylated derivatives, **14**. Unfortunately, the low and variable yields of the presumed linear mini-stambomycins **12** precluded their characterization beyond measurement of the expected masses at high-resolution (see **Supplementary Fig. 32**).

Experimental support for the structural assignments:

1. **Relatedness to the stambomycins.** Exact masses and retention times consistent with both the A/B and C/D variants of **12**−**14** are observed in all cases, as expected for derivatives of stambomycins A–D. MS analysis of **13** A/B and C/D shows several, conserved water losses (see below), again consistent with the proposed structures.

2. **Chemical composition.** The exact masses for **12**−**14** are in excellent agreement with structural prediction (see **Supplementary Fig. 32** and **Supplementary Table 2**). This observation intrinsically limits the structural possibilities to isomers of the same molecular formula, which could only arise either by aberrant action of the PKS or by spontaneous chemistry (see further discussion of these points below). In this context, the simplest explanation for the observed molecular weights is that all of the modules in Pks9 have functioned correctly, including module 24 which notably lacks an active KR domain, as opposed to alternative possibilities in which certain modules iterate while others are skipped.

3. **Precise molecular structure.**

a. *Shared substructures with metabolites **4**–**11** and **15**.* MS analysis of **13** A/B and C/D shows several, conserved water losses (compare **Supplementary Figs. 14**, **20** and **33**). (Note: as the MS signals for **13** overlap with those from other metabolites (unassigned peaks), it was not possible to analyze a larger mass range in order to detect additional water losses.)

Most critically, MS^2 analysis reveals members of the same series of low MW fragments (highlighted in grey) common to all novel metabolites **4**–**11** (159.1, 135.1, 109.1 and 95.08) (compare **Supplementary Figs. 15**, **21** and **34**). In addition to these low MW fragments, metabolites **13** and **14** share several additional fragments (351.2 and 239.2), while **13** also has multiple additional fragments in common with its deoxy analogue **15** (351.2, 333.2, 239.2 and 209.2 (shown in purple)) (see **Supplementary Fig. 34**). These shared fragmentation patterns confirm the relatedness of compounds **13**−**15**.

b. *Presence of a macrocycle (mode of chain release).*

To address this question, it is useful to consider alternative modes of chain release either by the TE or spontaneous chemistry that would give rise to the same molecular weight (I−III listed below, and see **Supplementary Fig. 35** for the possible structures, using **13** as an example). We also indicate in each case evidence that we've obtained which refutes the mechanism.

i) Dihydropyrone formation (attack on the thioester by the C5-OH).

Evidence against:

The presence of a peak at 174.1 in the MS^2 spectra of both **13** and **14** (highlighted in yellow above) shown previously (**Supplementary Fig. 34**) to be present in the macrocyclic parental structures and not in any of the linear metabolites (50-deoxystambomycins **2**A/B (see **Supplementary Fig. 10** (173.1 highlighted in mint)), or compounds **4**–**11**, **15** and **16** (see **Supplementary Fig. 15**, **21**, **36** and **39**)).

ii) Hydrolysis and tetrahydropyran formation and dehydration to form a double bond (shown in blue) (as described in [17]).

Evidence against: (In addition to point 1a above) There is no evidence in the MS spectra of **13** and **14** for loss of CO_2, while such fragments are observed for all of the linear derivatives incorporating this functionality (**4**–**11** (**Supplementary Figs. 14**, **20**), **15**–**17** (**Supplementary Fig. 38**), and 50-deoxystambomycins **2**A/B (**Supplementary Fig. 10**)). There is also no evidence that such dehydration occurs with the parental stambomycins which incorporate the tetrahydropyran[8].

iii) Cyclization with any of the other OH groups in the molecule (besides the terminal one shown in red) to give a smaller macrocycle.

Evidence against:
Genetically inactivating the hydroxylase SamR0479 responsible for introducing the terminal hydroxylation used for cyclization, supports both that macrocyclization occurs and its regiochemistry. Specifically, the product profile shifts from three compounds (**12**–**14**) to one compound (**15**, see **Supplementary Fig. 37**), whose exact mass and fragmentation pattern (see **Supplementary Figs. 38** and **39**) are fully consistent with a non-hydroxylated, linear product. Thus, macrocyclization was specifically disrupted by this mutation, which would not be the case if an alternative hydroxyl group were used in the cyclization, or in fact for *either of the other two chain-release mechanisms*.

Thus, while we cannot absolutely rule out alternative structures due to the lack of NMR data, the full weight of evidence clearly favors the structures proposed for **12**–**14**.

Supplementary references

1. Broadhurst, R. W., Nietlispach, D., Wheatcroft, M. P., Leadlay, P. F. & Weissman, K. J. The structure of docking domains in modular polyketide synthases. *Chem. Biol.* **10**, 723−731 (2003).
2. Buchholz, T. J., Geders, T. W., Bartley, F. E., Reynolds, K. A., Smith, J. L. & Sherman, D. H. Structural basis for binding specificity between subclasses of modular polyketide synthase docking domains. *ACS Chem. Biol.* **4**, 41−52 (2009).
3. Tang, Y., Chen, A. Y., Kim, C. Y., Cane, D. E. & Khosla, C. Structural and mechanistic analysis of protein interactions in module 3 of the 6-deoxyerythronolide B synthase. *Chem. Biol.* **14**, 931−943 (2007).
4. Gust, B., Challis, G. L., Fowler, K., Kieser, T. & Chater, K. F. PCR-targeted *Streptomyces* gene replacement identifies a protein domain needed for biosynthesis of the sesquiterpene soil odor geosmin. *Proc. Natl. Acad. Sci. U. S. A.* **100**, 1541−1546 (2003).
5. Raynal, A., Karray, F., Tuphile, K., Darbon-Rongère, E. & Pernodet, J.-L. Excisable cassettes: new tools for functional analysis of *Streptomyces* genomes. *Appl. Environ. Microbiol.* **72**, 4839−4844 (2006).
6. Cobb, R. E., Wang, Y. J. & Zhao, H. M. High-efficiency multiplex genome editing of *Streptomyces* species using an engineered CRISPR/Cas system. *ACS Synth. Biol.* **4**, 723−728 (2015).
7. Tong, Y., Charusanti, P., Zhang, L., Weber, T. & Lee, S. Y. CRISPR-Cas9 based engineering of actinomycetal genomes. *ACS Synth. Biol.* **4**, 1020−1029 (2015).
8. Laureti, L.*, et al.* Identification of a bioactive 51-membered macrolide complex by activation of a silent polyketide synthase in *Streptomyces ambofaciens*. *Proc. Natl. Acad. Sci. U. S. A.* **108**, 6258−6263 (2011).
9. Song, L., Laureti, L., Corre, C., Leblond, P., Aigle, B. & Challis, G. L. Cytochrome P450-mediated hydroxylation is required for polyketide macrolactonization in stambomycin biosynthesis. *J. Antibiot.* **67**, 71−76 (2014).
10. Heathcote, M. L., Staunton, J. & Leadlay, P. F. Role of type II thioesterases: evidence for removal of short acyl chains produced by aberrant decarboxylation of chain extender units. *Chem. Biol.* **8**, 207−220 (2001).
11. Böhm, G., Muhr, R. & Jaenicke, R. Quantitative analysis of protein far UV circular dichroism spectra by neural networks. *Protein Eng.* **5**, 191−195 (1992).
12. Alekseyev, V. Y., Liu, C. W., Cane, D. E., Puglisi, J.D. & Khosla, C. Solution structure and proposed domain domain recognition interface of an acyl carrier protein domain from a modular polyketide synthase. *Protein Sci.* **16**, 2093−2107 (2007).
13. Kapur, S.*, et al.* Reprogramming a module of the 6-deoxyerythronolide B synthase for iterative chain elongation. *Proc. Natl. Acad. Sci. U. S. A.* **109**, 4110−4115 (2012).
14. Kapur, S., Chen, A. Y., Cane, D. E. & Khosla, C. Molecular recognition between ketosynthase and acyl carrier protein domains of the 6-deoxyerythronolide B synthase. *Proc. Natl. Acad. Sci. U. S. A.* **107**, 22066−22071 (2010).
15. Wlodek, A.*, et al.* Diversity oriented biosynthesis via accelerated evolution of modular gene clusters. *Nat. Comm.* **8**, 1206 (2017).
16. Yuzawa, S.*, et al.* Comprehensive *in vitro* analysis of acyltransferase domain exchanges in modular polyketide synthases and its application for short-chain ketone production. *ACS Synth. Biol.* **6**, 139−147 (2017).
17. Koch, A. A. *et al.* Probing selectivity and creating structural diversity through hybrid polyketide synthases. *Angew. Chem. Int. Ed Engl.* **59**, 13575–13580 (2020).
