# Supplementary material for: Engineering the stambomycin modular polyketide synthase yields 37-membered mini-stambomycins
Source: Nat Commun. 2022 Jan 26;13:515. doi: 10.1038/s41467-022-27955-z (PMC8792006; doi:10.1038/s41467-022-27955-z)
Supplement: Supplementary file 3 — Description of Additional Supplementary Files [file 41467_2022_27955_MOESM3_ESM.pdf]

Description of Additional Supplementary Files

File Name: Supplementary Data 1

Description: Primers used in this work. This table lists the primer names, their sequences, how they were employed, and to which engineering strategy they are associated (PCR-based or CRISPR-Cas9-based).

File Name: Supplementary Data 2

Description: Plasmids and BACs used in this work. This table lists the names of plasmids used in this work, their principal characteristics and how they were employed.

File Name: Supplementary Data 3

Description: Strains used in this work. This table provides a description of the strains used in this work, and their antibiotic resistance.
