# Supplementary material for: Engineering the stambomycin modular polyketide synthase yields 37-membered mini-stambomycins
Source: Nat Commun. 2022 Jan 26;13:515. doi: 10.1038/s41467-022-27955-z (PMC8792006; doi:10.1038/s41467-022-27955-z)
Supplement: Supplementary file 7 — Reporting Summary [file 41467_2022_27955_MOESM7_ESM.pdf]

nature research

Corresponding author(s): Jacob, Christophe; Aigle, Bertrand; Weissman, Kira J

Last updated by author(s): Sep 17, 2021

Reporting Summary

Nature Research wishes to improve the reproducibility of the work that we publish. This form provides structure for consistency and transparency in reporting. For further information on Nature Research policies, see Authors & Referees and the Editorial Policy Checklist .

Statistics

For all statistical analyses, confirm that the following items are present in the figure legend, table legend, main text, or Methods section.

n/a	Confirmed	
☐	☒	The exact sample size (*n*) for each experimental group/condition, given as a discrete number and unit of measurement
☐	☒	A statement on whether measurements were taken from distinct samples or whether the same sample was measured repeatedly
☒	☐	The statistical test(s) used AND whether they are one- or two-sided<br>*Only common tests should be described solely by name; describe more complex techniques in the Methods section.*
☒	☐	A description of all covariates tested
☐	☒	A description of any assumptions or corrections, such as tests of normality and adjustment for multiple comparisons
☒	☐	A full description of the statistical parameters including central tendency (e.g. means) or other basic estimates (e.g. regression coefficient) AND variation (e.g. standard deviation) or associated estimates of uncertainty (e.g. confidence intervals)
☒	☐	For null hypothesis testing, the test statistic (e.g. *F*, *t*, *r*) with confidence intervals, effect sizes, degrees of freedom and *P* value noted<br>*Give P values as exact values whenever suitable.*
☒	☐	For Bayesian analysis, information on the choice of priors and Markov chain Monte Carlo settings
☒	☐	For hierarchical and complex designs, identification of the appropriate level for tests and full reporting of outcomes
☒	☐	Estimates of effect sizes (e.g. Cohen's *d*, Pearson's *r*), indicating how they were calculated

Our web collection on statistics for biologists contains articles on many of the points above.

Software and code

Policy information about availability of computer code

Data collection

MicroCal iTC200 (Malvern Instruments) for ITC measurements;
Chirascan CD (Applied Photophysics) for CD measurements;
MiniDAWN TREOS II (Wyatt Technology) and Optilab T-rEX (Wyatt Technology) for SEC-MALS analysis;
Thermo Scientific Orbitrap LTQXL or Orbitrap ID-X Tribrid, or Thermo ScientificTM Q ExactiveTM Hybrid Quadrupole-Orbitrap mass spectrometer for MS detection

Data analysis

Expasy ProtParma (https://web.expasy.org/protparam/) for in silico protein analysis (MW, theoretical pI, extinction coefficient, etc.)
Origin 7.0 (OriginLab) software for ITC data processing and presentation;
MicroCal Concat ITC software v. 1.00 for combining the data from two ITC files, and combining them into one;
CDNN2.1 software for CD data deconvolution;
ASTRA 6.1 (Wyatt Technology) for SEC-MALS data processing and presentation;
Thermo xcalibur qual browser 2.2 for MS analysis;
SIEVE 2.0 (Thermo Fischer Scientific) software for comparative metabolic analysis;
ChemBioDraw Ultra 14.0 for creation of chemical structures;
Microsoft PowerPoint 2016 for image processing;
Microsoft Excel 2016 for calculation and creation of CD spectra, standard curve and production titers;
Clustal Omega (https://www.ebi.ac.uk/Tools/msa/clustalo) for AA sequence alignment;
PSIPRED 4.0 (http://bioinf.cs.ucl.ac.uk/psipred/) for the secondary structure prediction of Docking domains;
PyMOL 2.4 educational-use-only for PDB file open and creation of high quality figures;
SnapGene viewer for Plasmid mapping and DNA sequence analysis

For manuscripts utilizing custom algorithms or software that are central to the research but not yet described in published literature, software must be made available to editors/reviewers. We strongly encourage code deposition in a community repository (e.g. GitHub). See the Nature Research guidelines for submitting code & software for further information.

Data

Policy information about availability of data

All manuscripts must include a data availability statement. This statement should provide the following information, where applicable:

- Accession codes, unique identifiers, or web links for publicly available datasets
- A list of figures that have associated raw data
- A description of any restrictions on data availability

All data supporting the findings of this study are available within the manuscript. The docking domain structures underpinning our DD analysis are available under the following accession codes (PDB ID): 1PZQ, 1PZR (type 1a) and 3F5H (type 1b). The raw HPLC-MS data have been deposited in the data repository DOREL (Données de la Recherche Lorraines) at https://doi.org/10.12763/PEYXHP. Source data are provided with this paper.

Field-specific reporting

Please select the one below that is the best fit for your research. If you are not sure, read the appropriate sections before making your selection.

[x] Life sciences [ ] Behavioural & social sciences [ ] Ecological, evolutionary & environmental sciences

For a reference copy of the document with all sections, see nature.com/documents/nr-reporting-summary-flat.pdf

Life sciences study design

All studies must disclose on these points even when the disclosure is negative.

Sample size	Metabolite production from every engineered strain was analyzed at least twice, but more typically three or more times (see Supplementary Table 5), to confirm the presence of specific metabolites and estimate their yields. This repetition was necessary as certain metabolites were only produced intermittently, and many present at the limits of detection using our methods. The standard curves for quantifying the metabolites were based on one or two measurements at each concentration. The reliability of this measurement was confirmed by the fact that essentially linear responses were obtained over the selected concentration ranges. Due to the fact that the methods provide cross-confirmatory data, the mass spectrometry and SEC-MALS analyses of the docking domains were carried out once each. The CD measurements of the docking domains were carried out in triplicate and at three distinct concentrations, providing internal confirmation for the reliability of the data. Due to the substantial quantities of protein required (especially in the case of the experiments requiring consecutive injections), each of the ITC measurements was carried out in duplicate only, and the average Kd values calculated.
Data exclusions	No data were excluded.
Replication	As shown in Supplementary Table 5, a clone of each strain was grown a minimum of twice, but more typically 3–5 times. In many cases, these independent fermentations were carried out on distinct dates over a period of months. The analyses were performed as the strains became available (the precise dates on which the analyses were carried out are indicated within the strain names (far left column)).
Randomization	Randomization is not relevant to our study, as each of the engineered strains had a specific control or controls, to which it was directly prepared.
Blinding	Blinding was not relevant to our study, as in order to interpret the data sensibly, we needed to know the identity of the particular strain which was being analyzed (i.e. which modifications had been introduced).

Reporting for specific materials, systems and methods

We require information from authors about some types of materials, experimental systems and methods used in many studies. Here, indicate whether each material, system or method listed is relevant to your study. If you are not sure if a list item applies to your research, read the appropriate section before selecting a response.

Materials & experimental systems

n/a	Involved in the study
[x]	[ ] Antibodies
[x]	[ ] Eukaryotic cell lines
[x]	[ ] Palaeontology
[x]	[ ] Animals and other organisms
[x]	[ ] Human research participants
[x]	[ ] Clinical data

Methods

n/a	Involved in the study
[x]	[ ] ChIP-seq
[x]	[ ] Flow cytometry
[x]	[ ] MRI-based neuroimaging
